# The relationship between central obesity and risk of breast cancer: a dose–response meta-analysis of 7,989,315 women

**DOI:** 10.3389/fnut.2023.1236393

**Published:** 2023-11-09

**Authors:** Hongyang Chen, Mengqi Yuan, Xiaomin Quan, Dongmei Chen, Jingshu Yang, Chenyang Zhang, Yunxin Nan, Fan Luo, Donggui Wan, Guowang Yang, Chao An

**Affiliations:** ^1^Graduate School, Beijing University of Chinese Medicine, Beijing, China; ^2^Department of Oncology, China-Japan Friendship Hospital, Beijing, China; ^3^Department of Oncology, Beijing Hospital of Traditional Chinese Medicine, Capital Medical University, Beijing, China; ^4^Capital Medical University, Beijing, China; ^5^Department of Oncology, Beijing of Chinese Medicine Second Affiliated Dong Fang Hospital, Beijing, China

**Keywords:** central obesity, breast cancer, meta-analysis, body mass index, hip circumference, waist circumference, waist-hip-ratio

## Abstract

**Purpose:**

Central obesity may contribute to breast cancer (BC); however, there is no dose–response relationship. This meta-analysis examined the effects of central obesity on BC and their potential dose–response relationship.

**Methods:**

In the present study, PubMed, Medline, Embase, and Web of Science were searched on 1 August 2022 for published articles. We included the prospective cohort and case–control studies that reported the relationship between central obesity and BC. Summary effect size estimates were expressed as risk ratios (RRs) or odds ratios (ORs) with 95% confidence intervals (95% CI) and were evaluated using random-effect models. The inconsistency index (*I*^2^) was used to quantify the heterogeneity magnitude derived from the random-effects Mantel–Haenszel model.

**Results:**

This meta-analysis included 57 studies (26 case–control and 31 prospective cohort) as of August 2022. Case–control studies indicated that waist circumference (WC) (adjusted OR = 1.18; 95% CI: 1.00–1.38; *P* = 0.051) and waist-to-hip ratio (WHR) (adjusted OR = 1.28; 95% CI: 1.07–1.53; *P* = 0.008) were significantly positively related to BC. Subgroup analysis showed that central obesity measured by WC increased the premenopausal (adjusted OR = 1.15; 95% CI: 0.99–1.34; *P* = 0.063) and postmenopausal (adjusted OR = 1.18; 95% CI: 1.03–1.36; *P* = 0.018) BC risk and the same relationship appeared in WHR between premenopausal (adjusted OR = 1.38; 95% CI: 1.19–1.59; *P* < 0.001) and postmenopausal (adjusted OR = 1.41; 95% CI: 1.22–1.64; *P* < 0.001). The same relationship was observed in hormone receptor-positive (HR+) (adjusted OR_WC_ = 1.26; 95% CI: 1.02–1.57; *P* = 0.035, adjusted OR_WHR_ = 1.41; 95% CI: 1.00–1.98; *P* = 0.051) and hormone receptor-negative (HR–) (adjusted OR_WC_ = 1.44; 95% CI: 1.13–1.83; *P* = 0.003, adjusted OR_WHR_ = 1.42; 95% CI: 0.95–2.13; *P* = 0.087) BCs. Prospective cohort studies indicated that high WC (adjusted RR = 1.12; 95% CI: 1.08–1.16; *P* < 0.001) and WHR (adjusted RR = 1.05; 95% CI: 1.018–1.09; *P* = 0.017) may increase BC risk. Subgroup analysis demonstrated a significant correlation during premenopausal (adjusted RR = 1.08; 95% CI: 1.02–1.14; *P* = 0.007) and postmenopausal (adjusted RR = 1.14; 95% CI: 1.10–1.19; *P* < 0.001) between BC and central obesity measured by WC, and WHR was significantly positively related to BC both premenopausal (adjusted RR_pre_ = 1.04; 95% CI: 0.98–1.11; *P* = 0.169) and postmenopausal (adjusted RR_post_ = 1.04; 95% CI: 1.02–1.07; *P* = 0.002). Regarding molecular subtype, central obesity was significantly associated with HR+ (adjusted OR_WC_ = 1.13; 95% CI: 1.07–1.19; *P* < 0.001, adjusted OR_WHR_ = 1.03; 95% CI: 0.98–1.07; *P* = 0.244) and HR– BCs (adjusted OR_WC_ =1.11; 95% CI: 0.99–1.24; *P* = 0.086, adjusted OR_WHR_ =1.01; 95% CI: 0.91–1.13; *P* = 0.808). Our dose–response analysis revealed a J-shaped trend in the relationship between central obesity and BC (measured by WC and WHR) in case–control studies and an inverted J-shaped trend between BMI (during premenopausal) and BC in the prospective cohort.

**Conclusion:**

Central obesity is a risk factor for premenopausal and postmenopausal BC, and WC and WHR may predict it. Regarding the BC subtype, central obesity is proven to be a risk of ER+ and ER- BCs. The dose–response analysis revealed that when BMI (during premenopausal) exceeded 23.40 kg/m^2^, the risk of BC began to decrease, and WC higher than 83.80 cm or WHR exceeded 0.78 could efficiently increase the BC risk.

**Systematic review registration:**

https://www.crd.york.ac.uk/PROSPERO/, identifier: CRD42022365788.

## Introduction

Breast cancer (BC) has surpassed lung cancer as the most commonly diagnosed cancer, with an estimated 2.3 million new cases accounting for ~11.7% of all cancers (1). BC has become a global public health concern accompanied by a large financial burden on healthcare systems (2). Given this disease burden, identifying potentially modifiable factors associated with BC development is of public health significance.

Studies have linked high BC risk to age, age of menarche and menopause, childbearing, nursing, family history, genetic risk, mammographic density, previous benign breast disease, radiation, obesity, oral contraceptives, hormonal replacement treatment, and diabetes mellitus (3, 4). Despite being beneficial in premenopausal women, high BMI may raise breast cancer risk in postmenopausal women (3). Obesity has become a global epidemic in recent decades. It can cause many ailments (5). It is widely accepted that central obesity is a serious risk factor for illnesses related to obesity, and the accumulation of visceral fat promotes the release of pro-oxidants, pro-inflammatory, and reactive oxygen species (ROS) (6).

The research found that the World Cancer Research Fund diagnosed women with a waist circumference (WC) > 85 cm or a waist–hip ratio (WHR) > 0.85 as central obesity (7). Central obesity increases BC risk (8). A 2003 systematic review found that WC and WHR increased postmenopausal BC but not premenopausal BC in cohort studies with the most adjusted data (without weight or BMI adjustment). The BMI adjustment of the limited cohort abolished this relationship but introduced premenopausal BC (9). Evidence from a 2016 dose–response meta-analysis of prospective studies reported that central obesity was measured by WC but not by WHR. Moreover, WC was related to premenopausal (RR_per_ 10-cm increase = 1.09, 95% CI: 1.02–1.16, *I*^2^ = 0%) and postmenopausal BC (RR_per_ 10-cm increase = 1.05, 95% CI: 1.02–1.08, *I*^2^ = 6.3%) when considering body mass index (BMI) adjusted RRs (10). However, a recent update study indicated that higher WC was not associated with premenopausal BC, while postmenopausal BC risk was significantly positively related to all adiposity measures evaluated (11). These studies are contradictory and lack the analysis of measurement metrics for BC analysis subtype studies.

Despite decades of studies, the association between central obesity and BC risk is contentious due to menstrual cycles, and the comprehensive meta-analysis and dose–response relationship is uncertain. This meta-analysis aimed to detect the relationship between central obesity and BC risk and to conduct a dose–response analysis to explore the linear relationship between them.

## Method

### Protocol and registration

Our study rigorously followed Preferred Reporting Items for Systematic Review and Meta-Analysis (PRISMA) guidelines. PRISMA checklist is shown in Supplementary Table 1. The study has been registered on the PROSPERO for systematic reviews (registration number CRD42022365788).

### Search strategy

In the current study, PubMed, Medline, Embase, and Web of Science were searched with medical terms to identify the potential eligible articles that reported the association of central obesity and the risk of BC until August 2022. Supplementary Table 2 displays the full details of our search strategy.

### Inclusion/exclusion criteria

Studies that met the following criteria simultaneously were included: (1) population: BC-diagnosed women; (2) exposure: body mass index (BMI), hip circumference (HC), waist circumference (WC), and waist–hip ratio (WHR) were the exposure of interest; (3) outcome: the study outcome was BC; (4) estimate effect: relative risks (RR)s or odd ratios (OR)s and 95% confidence intervals (CIs) of the association between obesity and BC were reported; (5) study design: prospective cohort or case–control studies, including retrospective and nested case–control cohorts; (6) only articles in the English language were chosen.

The exclusion criteria of the meta-analysis were as follows: (1) publications without original data, such as reviews, editorials, and comments; (2) used specific body fat content as the exposure, such as abdominal or leg fat mass; (3) unpublished articles. (4) To avoid the influence of different races on the categorization of central obesity, studies on race-specific studies were excluded.

The most complete one was included when multiple publications from the same study were available.

#### Study selection

Endnote version 20 was used for literature management to file the search records of the articles. The study was selected by two authors (MY and HC) following three steps: First, they screened all retrieved article titles and included them if at least one reviewer found them eligible. It was included in the abstract review stage if there was any doubt. Second, two independent reviewers reassessed abstract eligibility using inclusion and exclusion criteria. Finally, two independent reviewers assessed the full text using standardized eligibility criteria and included the final eligible articles. If the reviewers disagreed with any of the three processes, a third independent reviewer (X. Q.) from our group made a decision following a discussion.

#### Data extraction

Two investigators (HC and MQ) independently extracted data for the qualified studies, which included the following items: first author, publication year, country of study, sample size, study type, population age, number of cases, follow-up years, estimated effects for all categories, menstrual status, measurement index, and type and status of hormone receptors. When studies reported multiple RRs or ORs, we used the effect size that maximally adjusted for potentially confounding variables. Any disagreement was resolved by consensus.

The main outcome of our research was the impact of central obesity on the BC risk. The secondary outcome was the association of central obesity with the risk of developing BC in different BC subtypes [e.g., estrogen receptor-positive (ER+) BC and estrogen receptor-negative (ER–) BC]. All relevant data from eligible articles were adjusted for relevant confounders.

### Quality evaluation

The Newcastle–Ottawa Scale (NOS) tool (12) was used to assess the bias risk in selected studies, evaluated by two reviewers (HC and MQ) independently. The NOS tool contains eight items, which can be categorized into three dimensions for prospective cohort or case–control studies: selection (four items, one star each), comparability (one item, up to two stars), and outcome (three items, one star each). Research with scores of “0–3”, “4–6,” and “7–9” was regarded as “low,” “medium,” and “high” quality, respectively (13). Any disputes arising from the quality evaluation will be handed over to a third reviewer (MY) for adjudication.

### Data synthesis and statistical analysis

STATA software version 14.1 for Windows (StataCorp, College Station, TX, USA) was used to assess the data management and analysis. A two-tailed *p*-value < 0.05 was considered statistically significant. Our meta-analysis reported effect sizes as risk ratios (RRs) with 95% confidence intervals (CIs) in cohort studies and odds ratios (ORs) with 95% CIs in case–control studies. We transformed studies that reported effect estimates as hazard ratios (HRs) to risk ratios (RRs) using Zhang's technique (14). The random-effects model (DerSimonian and Laird method) was employed to generate summary RRs and 95% CIs.

Greenland and Longnecker's generalized least-squares trend (GLST) estimation approach was used to estimate study-specific slopes across the measurement index in linear dose–response analyses using STATA version 14.1. The restricted cubic splines of exposure distributions with three knots (25th, 50th, and 75th percentiles) were used to test non-linearity between central obesity and BC.

Statistical heterogeneity across the included studies was assessed using the Q statistic (15), and inconsistency was quantified by the *I*^2^ statistic (16). Sensitivity analyses were conducted by omitting one study each time to assess the impact of individual studies on combined ORs or RRs (17) (Supplementary Figures 1, 2). Potential publication bias was evaluated with Begg's (18) and Egger's tests (19). We used Duval and Tweedie's trim-and-fill methods to detect the effect of possible missing studies on the overall effect (20).

Since WC and HC were not measured by unified units in various included studies, heterogeneous units were converted to centimeters (cm) for analysis. If data were available, we performed subgroup analyses to study whether central obesity and BC risk differed by study design, geographical location, follow-up years, menopause status, BC subtype, and measurement index.

## Results

### Eligible studies

**Figure 1:**
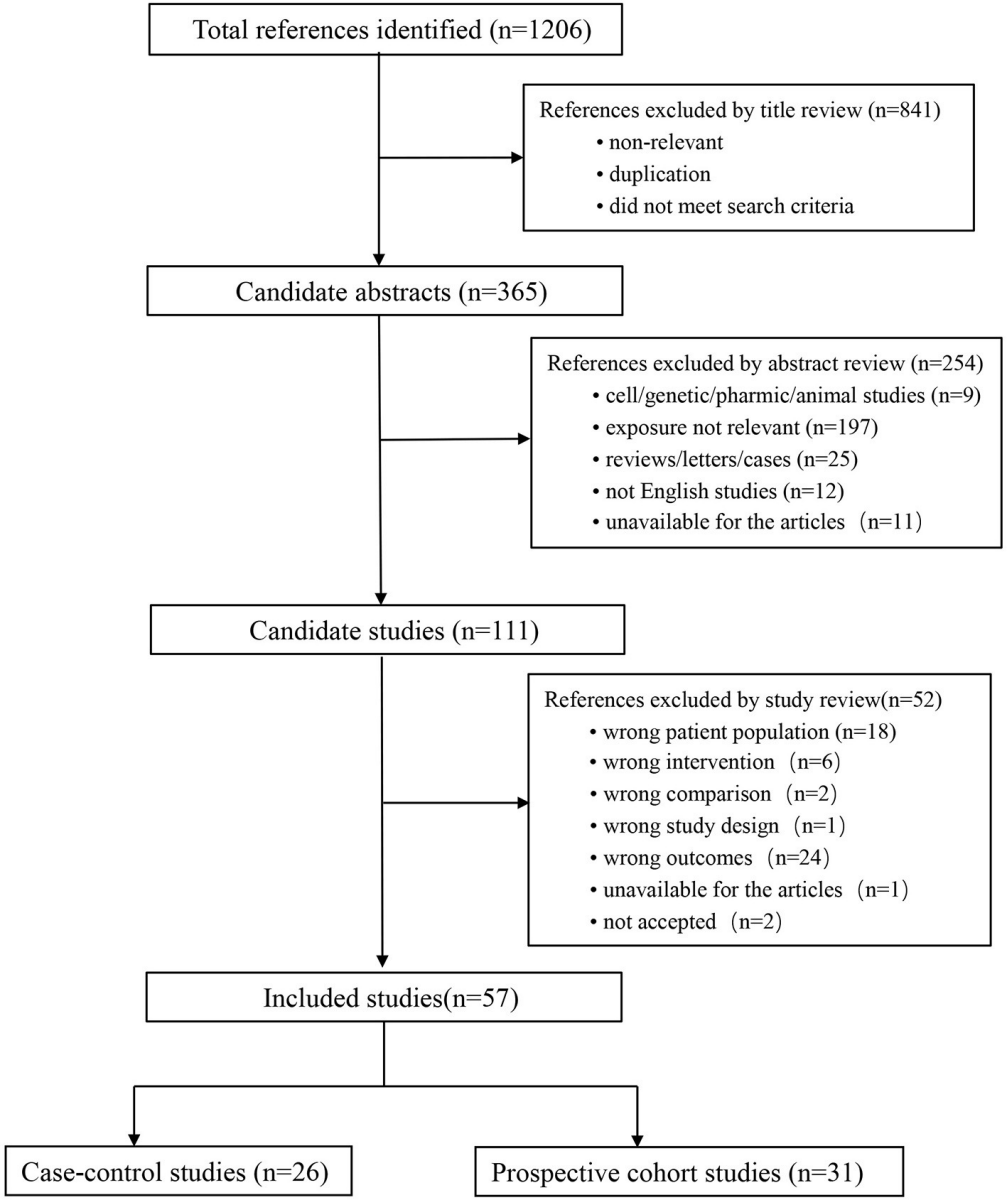
Flow chart of records retrieved, screened, and included in this meta-analysis.

The detailed selection process is schematized in Figure 1. Our initial search yielded 1,206 records. After excluding 841 irrelevant articles by title and abstract screening, 365 full texts were carefully reviewed. Finally, 57 publications with 7,979,624 participants were included in this dose–response meta-analysis, including 27 case–control and 30 prospective cohort studies. All included studies were original studies published between 1990 and 2022.

### Study characteristics

Supplementary Tables 3, 4 show the baseline characteristics of case–control and prospective cohort studies, respectively. Of the 57 eligible articles, 26 (21–46) studies were case–control studies and 31 (47–77) studies were prospective cohort studies. The studies were widely distributed in all regions of the world, of which 11 (24, 33–35, 42, 43, 45, 46, 63, 69, 77) were conducted in Asia, 18 (32, 39, 40, 47–49, 52, 53, 60, 62, 64, 66, 67, 71, 74, 75) in Europe, and 17 (22, 44, 50, 51, 55, 57–59, 61, 70, 72, 73, 76) in North America. South America, Africa, Oceania, and Australia each had six (26, 27, 30, 31, 41, 68), five (21, 29, 36–38), and two (56, 65) studies. In 26 (21–46) case–control studies, 107,691 participants and 24,241 cases were analyzed in the meta-analysis. In 31 (21–46) prospective cohort studies, 7,945,816 participants and 100,644 cases were analyzed.

#### Quality assessment

Supplementary Tables 5, 6 show the quality assessment of all eligible articles by the NOS tool for cohort studies. In case–control studies, only five showed a moderate NOS rating, while the remaining 21 were rated “high”. In prospective cohort studies, five studies had an NOS rating of “moderate”, and 22 had a high NOS rating. During the quality assessment, all the included studies that met the eligibility criteria were of moderate or high quality. Thus, no low-quality study was excluded from this study.

#### Sensitive analyses

Sensitivity analysis of included studies was performed by omitting one study each time. Our results were robust as sensitivity analysis indicated that a single study did not affect the overall impact size estimate (OR or RR).

#### Meta-analysis of BMI and BC

**Figure 2:**
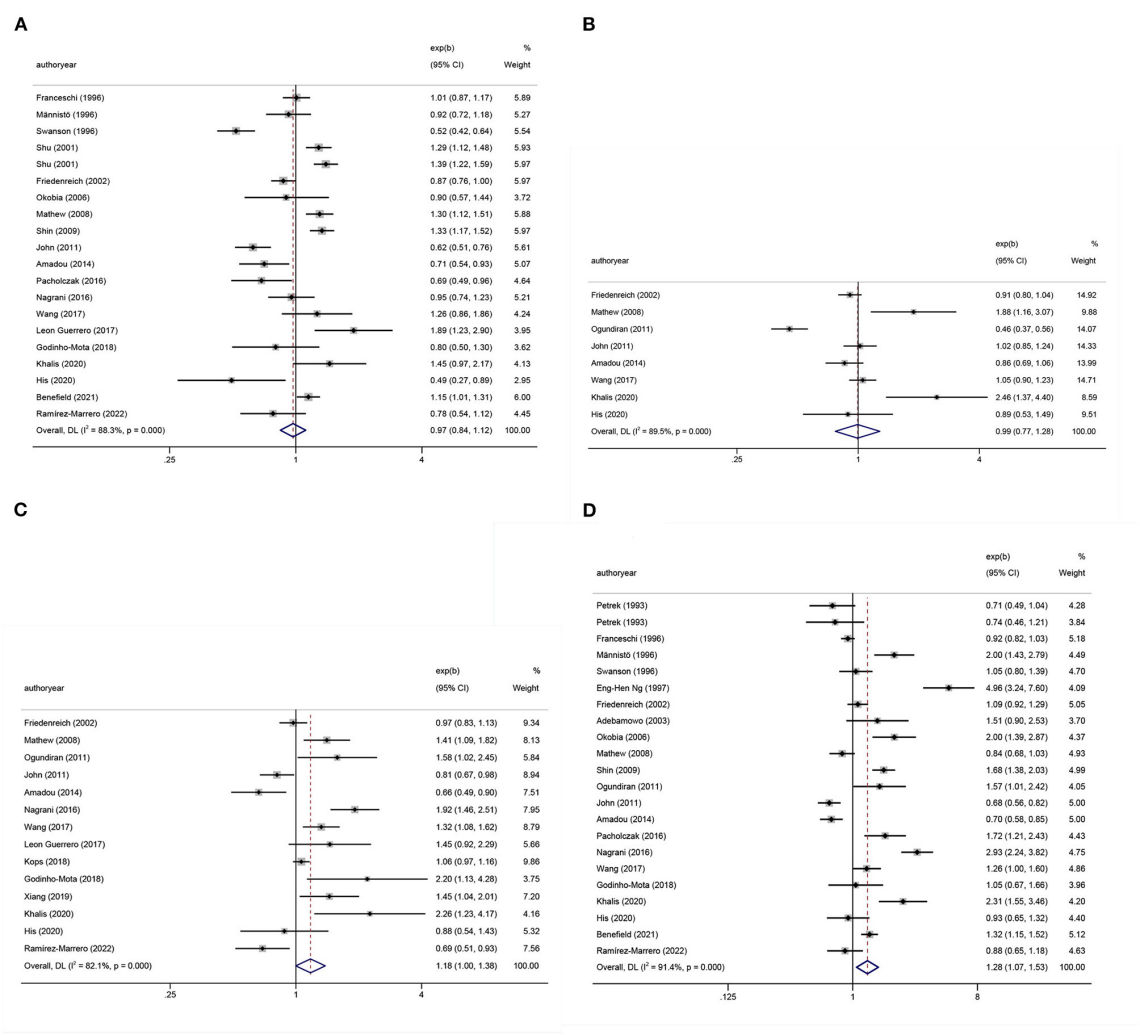
Forest plots of odds ratios (ORs) with corresponding 95% confidence intervals (CIs) of breast cancer risk for **(A)** body mass index (BMI), **(B)** hip circumference (HC), **(C)** waist circumference (WC), and **(D)** waist–hip ratio (WHR).

**Figure 3:**
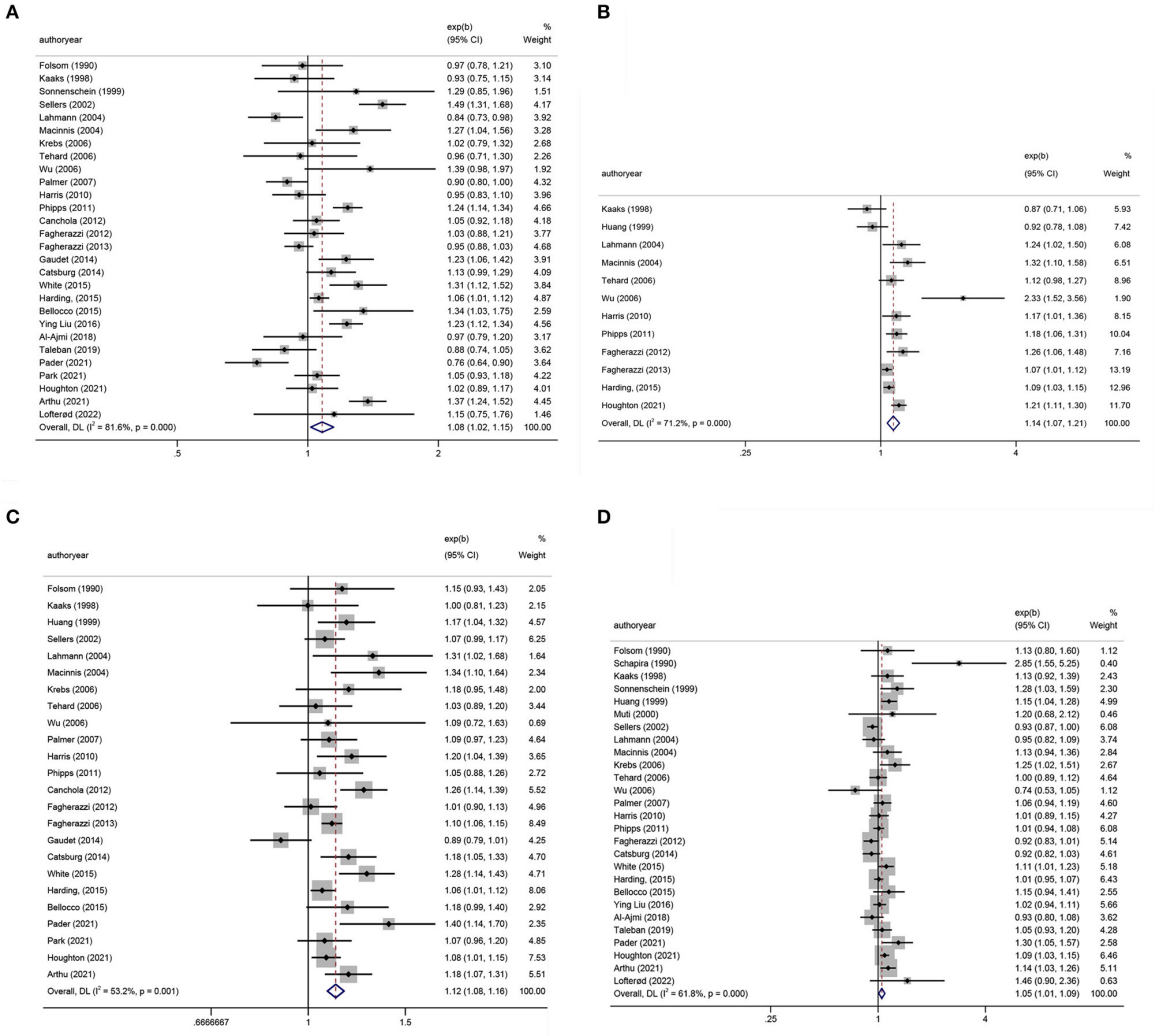
Forest plots of relative risks (RRs) with corresponding 95% confidence intervals (CIs) of breast cancer risk for **(A)** body mass index (BMI), **(B)** hip circumference (HC), **(C)** waist circumference (WC), and **(D)** waist–hip ratio (WHR).

Tables 1, 2 show the multifactor-adjusted risk estimates generated from the case–control and prospective cohort studies. OR showed no statistically significant BMI associations (adjusted OR = 0.98; 0.88–1.11; *P* = 0.792) (Figure 2A). According to RR, BC was related to significant BMI changes (adjusted RR = 1.08; 1.01–1.16; *P* = 0.035) (Figure 3A).

**Table 1:** Overall and subgroup analyses of the association between UA levels and the risk of stroke.

**Groups**	**Studies (*n*)**	**BMI**		**HC**		**WC**		**WHR**	
		**OR (95%CI);** ***P***	*I* ^2^	**OR (95%CI);** ***P***	*I* ^2^	**OR (95%CI);** ***P***	*I* ^2^	**OR (95% CI);** ***P***	*I* ^2^
**Overall analysis**									
Total	21/8/14/22	0.97 (0.84–1.12); 0.676	94.00%	0.99 (0.77–1.28); 0.958	96.00%	1.18 (1.00–1.38); 0.051	92.20%	1.28 (1.07–1.53); 0.008	95.60%
**Subgroup analysis**									
**By location**									
Asia	6/2/4/7	1.23 (1.10–1.36); < 0.001	62.90%	1.34 (0.77–2.35; 0.303	79.70%	1.49 (1.27–1.76); < 0.001	38.40%	1.65 (1.07–2.55); 0.023	96.10%
Europe	3/0/0/3	0.81 (0.61–1.09); 0.162	49.40%	NA	NA	NA	NA	1.18 (0.69–2.01); 0.543	87.20%
North America	5/3/3/5	0.75 (0.56–1.00); 0.051	92.60%	0.93 (0.84–1.02); 0.127	0.00%	0.83 (0.68–1.02); 0.071	64.10%	0.94 (0.70–1.24); 0.651	91.30%
South America	4/1/5/3	0.89 (0.53–1.51); 0.667	81.40%	0.89 (0.53–1.49); 0.654	0.00%	1.07 (0.80–1.43); 0.637	72.70%	0.93 (0.76–1.14); 0.465	0.00%
Africa	3/2/2/5	1.16 (0.73–1.85); 0.807	56.80%	1.04 (0.20–5.39); 0.967	88.30%	1.78 (1.25–2.55); 0.001	0.00%	1.88 (1.52–2.32); < 0.001	0.00%
**By menopause status**									
Pre-menopause	16/8/14/19	0.92 (0.82–1.03); 0.146	71.10%	0.92 (0.75–1.12); 0.392	80.90%	1.15 (0.99–1.34); 0.063	79.60%	1.38 (1.19–1.59); < 0.001	85.50%
Post-menopause	14/6/12/17	1.14 (1.03–1.26); 0.011	53.40%	0.93 (0.72–1.20); 0.565	79.80%	1.18 (1.03–1.36); 0.018	68.20%	1.41 (1.22–1.64; < 0.001	79.80%
**Subtype of BC**									
HR-	4/2/3/4	1.02 (0.85–1.23); 0.803	60.90%	0.93 (0.78–1.11); 0.435	0.00%	1.44 (1.13–1.83); 0.003	63.00%	1.42 (0.95–2.13); 0.087	90.00%
HR+	4/2/3/4	0.97 (0.80–1.19); 0.791	83.90%	1.00 (0.87–1.16); 0.967	28.00%	1.26 (1.02–1.57); 0.035	83.20%	1.41 (1.00–1.98); 0.051	93.50%

OR, odds ratio; 95% CI, 95% confidence interval; BMI, body mass index; WC, waist circumference; HC, hip circumference; WHR, waist-hip-ratio; NA, not available; HR, hormone receptor -positive (estrogen receptor-positive or progesterone receptor-positive); HR–, hormone receptor- negative (estrogen receptor-negative and progesterone receptor-negative); UC, un classified.

**Table 2:** Overall and subgroup analyses of the association between UA levels and the risk of stroke.

**Groups**	**Studies (*n*)**	**BMI**		**HC**		**WC**		**WHR**	
		**RR (95%CI);** ***P***	*I* ^2^	**RR (95%CI);** ***P***	*I* ^2^	**RR (95%CI);** ***P***	*I* ^2^	**RR (95% CI);** ***P***	*I* ^2^
**Overall analysis**									
Total	28/12/24/27	1.08 (1.02–1.15); 0.015	89.90%	1.14 (1.07–1.21); < 0.001	87.60%	1.12 (1.08–1.16); < 0.001	73.80%	1.05 (1.01−1.09); 0.017	77.90%
**Subgroup analysis**									
**By location**									
Asia	3/1/2/2	1.17 (1.02–1.34); 0.023	61.50%	2.33 (1.52–3.56); < 0.001	0.00%	1.07 (0.96–12); 0.211	0.00%	0.91 (0.68–1.23); 0.548	66.30%
Europe	11/5/8/12	1.00 (0.88–1.13); 0.943	84.20%	1.10 (1.00–1.21); 0.052	60.50%	1.12 (1.05–1.20); < 0.001	48.30%	1.08 (0.99–1.23); 0.100	66.70%
North America	11/4/11/10	1.15 (1.05–1.25); 0.002	73.20%	1.13 (1.02–1.25); 0.022	73.70%	1.13 (1.07–1.20); < 0.001	64.50%	1.06 (0.99–1.12); 0.081	69.80%
South America	1/0/1/1	0.90 (0.80–1.00); 0.053	0.00%	NA	NA	1.09 (0.97–1.23); 0.148	0.00%	1.06 (0.94–1.19); 0.331	0.00%
Oceania	2/2/2/2	1.13 (1.02–1.15); 0.160	67.10%	1.17 (0.98–1.04); 0.084	71.20%	1.17 (0.93–1.47); 0.181	80.10%	1.03 (0.95–1.12); 0.491	61.80%
**By follow up years**									
(0, 5)	3/2/3/4	0.89 (0.80–1.00); 0.460	0.00%	1.15 (1.03–1.28); 0.010	0.00%	1.13 (0.99−1.29); 0.081	24.50%	1.12 (0.89–1.40); 0.330	75.50%
(5, 10)	9/4/8/10	1.22 (1.11–1.34); < 0.001	73.40%	1.16 (1.00–1.33); 0.052	72.40%	1.13 (1.06–1.21); < 0.001	53.80%	1.05 (0.98–1.12); 0.194	71.50%
(10, +∞)	14/6/13/11	1.04 (0.97–1.12); 0.267	75.80%	1.12 (1.03–1.23); 0.010	79.80%	1.12 (1.06–1.17); < 0.001	60.50%	1.05 (1.00–1.11); 0.068	52.90%
**By menopause status**									
Pre-menopause	12/7/11/14	0.91 (0.87–0.94); < 0.001	33.60%	1.07 (0.97–1.17); 0.175	42.60%	1.08 (1.02–1.14); 0.007	56.80%	1.04 (0.98–1.10); 0.188	28.90%
Post-menopause	20/9/20/21	1.19 (1.03–1.24); < 0.001	84.50%	1.09 (1.05–1.13); < 0.001	0.00%	1.14 (1.10–1.19); < 0.001	67.50%	1.05 (1.02–1.07); 0.001	5.30%
**Subtype of BC**									
HR–	6/5/8/6	1.10 (1.01–1.19); 0.024	0.00%	1.31 (1.05–1.62); 0.017	62.40%	1.11 (0.99–1.24); 0.086	50.80%	1.01 (0.91–1.13); 0.808	38.90%
HR+	6/5/8/6	1.28 (1.18–1.38); < 0.001	73.80%	1.09 (1.02–1.15); 0.009	37.40%	1.13 (1.07–1.19); < 0.001	49.00%	1.03 (0.98–1.07); 0.244	40.40%

RR, relative risk; 95% CI, 95% confidence interval; BMI, body mass index; WC, waist circumference; HC, hip circumference; WHR, waist-hip-ratio; NA, not available; HR, hormone receptor -positive (estrogen receptor-positive or progesterone receptor-positive); HR–, hormone receptor- negative (estrogen receptor-negative and progesterone receptor-negative); UC, un classified.

#### Publication bias

**Figure 4:**
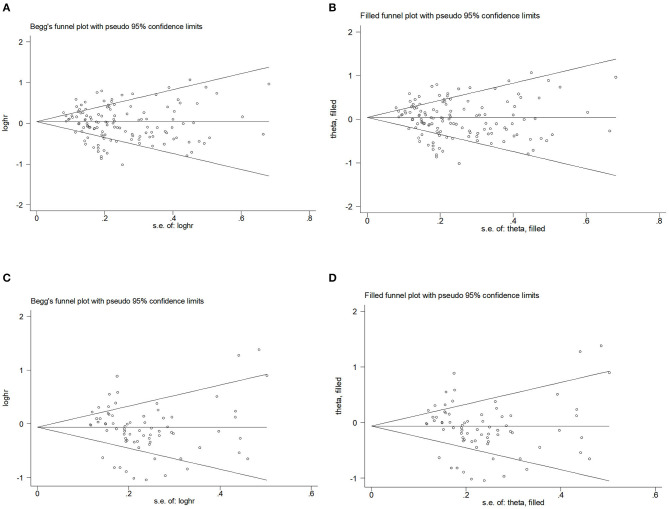
Begg's funnel plot and filled funnel plots of included studies showing odds ratios (ORs) and relative risks (RRs) for body mass index (BMI). **(A)** Begg's funnel plot, **(B)** filled funnel plot of OR, **(C)** Begg's funnel plot, and **(D)** filled funnel plot of RR.

To assess publication bias, Figures 4A–D presents Begg's filled funnel plots for the association with BMI in BC of OR (Figures 4A, B) and RR (Figures 4C, D). Begg's funnel plots seemed not perfectly symmetrical in RR but symmetrical in OR. Egger's test demonstrated some potential publication bias by OR (*P*_OR_ = 0.047, *P*_RR_ = 0.138), but no substantial changes in the data after trim-and-fill analysis. Moreover, the filled funnel plots revealed no missing studies in theory. The subjective nature of Begg's funnel plots suggests that publication bias did not affect the robustness of the results.

#### Subgroup analysis

We performed subgroup analyses to evaluate the cause of between-study heterogeneity (Tables 1, 2). Significant heterogeneities were found in the study location, follow-up years, menopause status, and BC subtype.

The subgroup analysis of location OR showed a positive relationship between BMI and BC risk in Asia (adjusted OR = 1.23; 95% CI: 1.10–1.36; *P* < 0.001). In the subgroup analysis of RR, in both Asia (adjusted RR = 1.17; 95% CI: 1.02–1.34; *P* = 0.023) and North America (adjusted RR = 1.15; 95% CI: 1.05–1.25; *P* = 0.002), BMI was associated with a facilitative effect on BC.

In the subgroup analysis of follow-up time, regarding RR, the results became statistically significant when the follow-up was 5–10 years (adjusted RR = 1.22; 95% CI: 1.11–1.34; *P* < 0.001).

In the subgroup analysis of menstrual status, premenopausal obesity (adjusted OR = 0.92; 95% CI: 0.82–1.03; *P* = 0.146) inhibited the BC development, while postmenopausal (adjusted OR = 1.14; 95% CI: 1.03–1.26; *P* = 0.011) obesity promoted it. When the effect quantity for RR, premenopausal (adjusted RR = 0.91; 95% CI: 0.87–0.94; *P* < 0.001) and postmenopausal (adjusted RR = 1.19; 95% CI: 1.03–1.24; *P* < 0.001) obesity had consistent results as OR.

No BC subtype showed statistical significance by OR in subgroup analysis. Regarding RR, BMI was related to BC development in a promotive role, regardless of HR– BC (adjusted RR = 1.10; 95% CI: 1.01–1.19; *P* = 0.024) or HR+ BC (adjusted RR = 1.28; 95% CI: 1.18–1.38; *P* < 0.001).

#### Meta-analysis of HC and BC

According to OR, there were no statistically significant associations of HC (adjusted OR = 0.99; 0.77–1.28; *P* = 0.958) (Figure 2B). Regarding RR, BC was associated with significant changes in HC (adjusted RR = 1.14; 1.07–1.21; *P* < 0.001) (Figure 3B).

#### Publication bias

**Figure 5:**
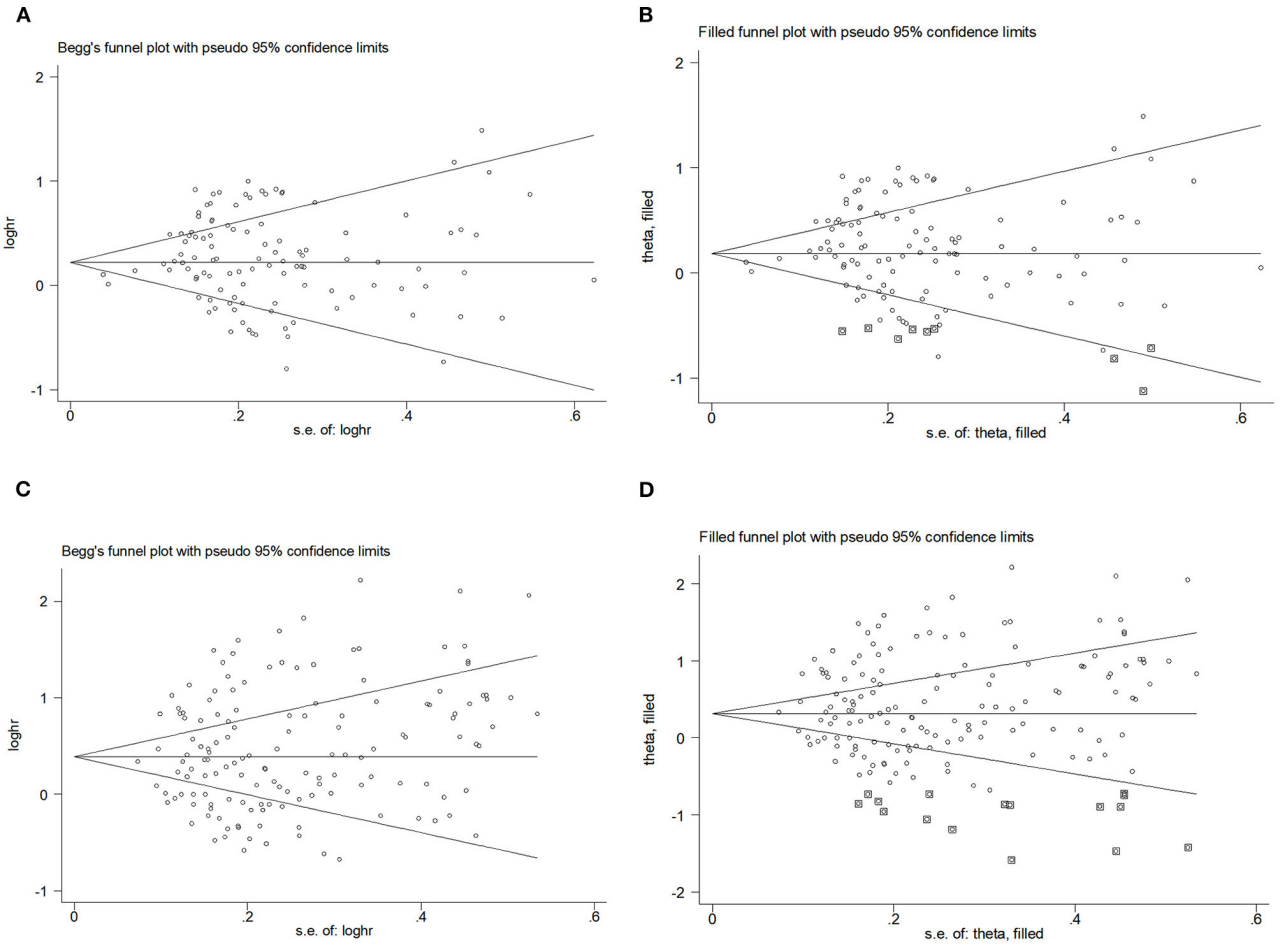
Begg's funnel plot and filled funnel plots of included studies showing odds ratios (ORs) and relative risks (RRs) for hip circumference (HC). **(A)** Begg's funnel plot, **(B)** filled funnel plot of OR, **(C)** Begg's funnel plot, and **(D)** filled funnel plot of RR.

Begg's funnel plot and Egger's test were used to assess publication bias. The results showed that Begg's funnel plots were symmetrical in OR (Figures 5A, B) and RR (Figures 5C, D), Egger's test of OR and RR (P_OR_ = 0.195, P_RR_ = 0.198) showed that there was no evidence of publication bias. The trim-and-fill analysis showed no substantial changes in OR and RR.

#### Subgroup analysis

The subgroup analysis of location OR showed that HC showed no statistical significance in any location. Geographically, HC had a more significant promoting effect on BC development in Asia (adjusted RR = 2.33; 95% CI: 1.52–3.56; *P* < 0.001) and a weaker effect in North America (adjusted RR = 1.13; 95% CI: 1.02–1.25; *P* = 0.022).

In the subgroup analysis of follow-up time, studies showed that for RR, the promotion effect of WHR on BC was statistically significant when the follow-up time was <5 years (adjusted RR = 1.15; 95% CI: 1.03–1.28; *P* = 0.010) and >10 years (adjusted RR = 1.12; 95% CI: 1.03–1.23; *P* = 0.010).

In the subgroup analysis of menstrual status, no statistical significance appeared in OR. Regarding RR, we observed only postmenopausal obesity was associated with BC (adjusted RR = 1.09; 95% CI: 1.05–1.13; *P* < 0.001).

In the subgroup analysis of the BC subtype, regardless of molecular typing, the BC is independent of HC by OR. Regarding RR, HR+ BC (adjusted RR = 1.09; 95% CI: 1.02–1.15; *P* = 0.009) and HR- BC (adjusted RR = 1.31; 95% CI: 1.05–1.62; *P* = 0.017) are associated with HC.

#### Meta-analysis of WC and BC

The summary OR of BC was 1.18 (95% CI: 1.00–1.38; *P* = 0.051) (Figure 2C). According to RR, BC was similarly related to significant changes in WC (adjusted RR = 1.12; 1.08–1.16; *P* < 0.001) (Figure 3C).

#### Publication bias

**Figure 6:**
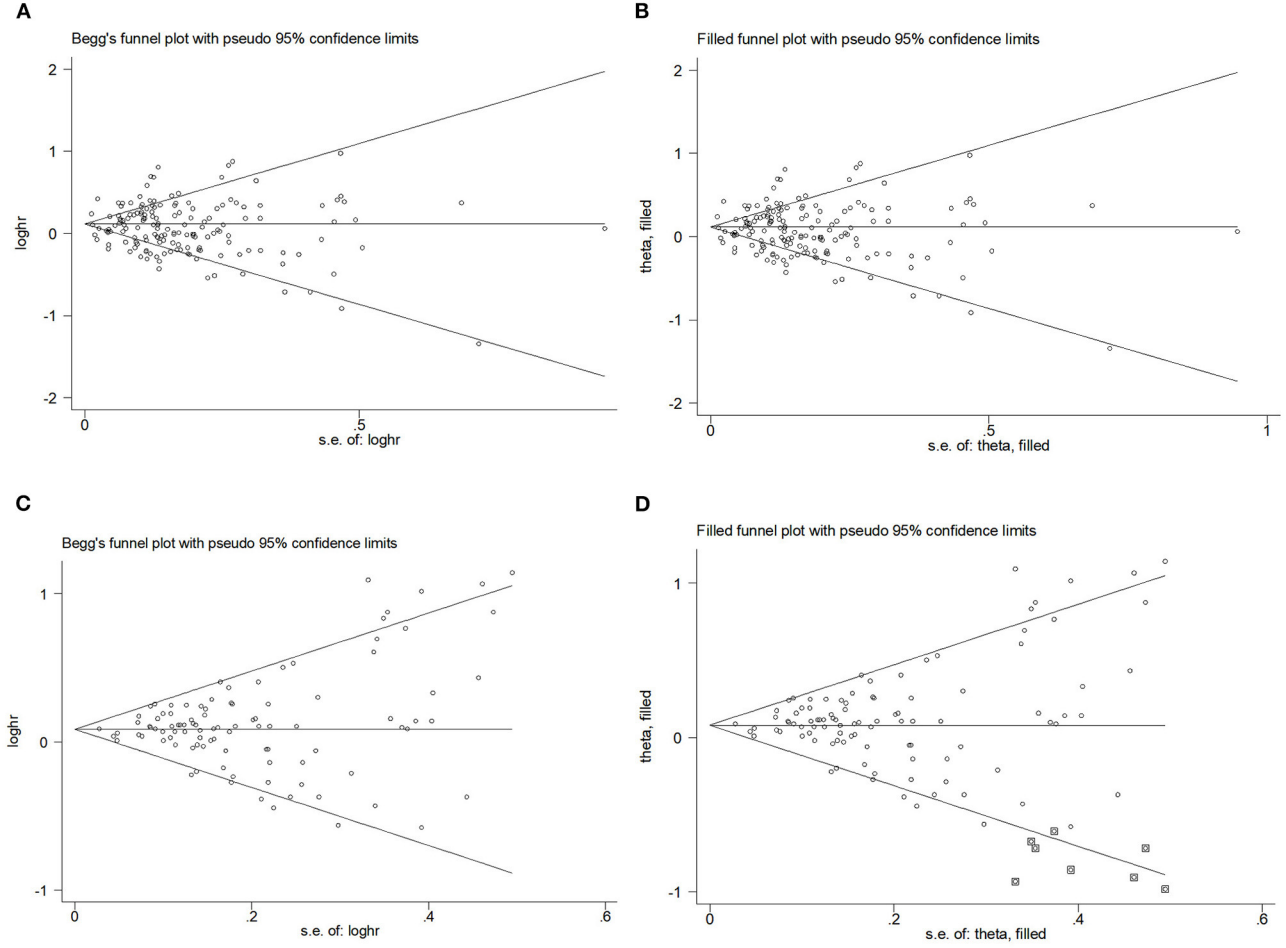
Begg's funnel plot and filled funnel plots of included studies showing odds ratios (ORs) and relative risks (RRs) for waist circumference (WC). **(A)** Begg's funnel plot, **(B)** filled funnel plot of OR, **(C)** Begg's funnel plot, and **(D)** filled funnel plot of RR.

Begg's funnel plot and Egger's test. Figures 6A–D presents Begg's filled funnel plots for the association with WC in BC. The symmetry of Begg's funnel plots by OR (Figures 6A, B) and RR (Figures 6C, D) is perfect. Egger's funnel plot showed that the study had some missing data in both OR and RR. Only Egger's test of OR indicates potential publication bias (*P*_OR_ = 0.038, *P*_RR_ = 0.832) but no substantial change in the data after trim-and-fill analysis, so potential publication bias did not affect the robustness of the results.

#### Subgroup analysis

The subgroup analysis of location revealed that WC promoted BC by OR in Asia (adjusted OR = 1.49; 95% CI: 1.27–1.76; *P* < 0.001). In the summary of RR, in Europe (adjusted RR = 1.12; 95% CI: 1.05–1.20; *P* < 0.001), North America (adjusted RR = 1.15; 95% CI: 1.11–1.20; *P* < 0.001), and Oceania (adjusted RR= 1.13; 95% CI: 1.07–1.20; *P* < 0.001), WC had a facilitative effect on BC.

The subgroup analysis of follow-up time by RR revealed a statistically significant association with BC after more than 5-year follow-up time (adjusted RR = 1.13; 95% CI: 1.06–1.21; *P* < 0.001; adjusted RR = 1.12; 95% CI: 1.06–1.17; *P* < 0.001).

The subgroup analysis of menstrual status OR showed that a statistically significant difference in promoting BC with WC was equally indicated in premenopausal (adjusted OR = 1.15; 95% CI: 0.99–1.34; *P* = 0.063) and postmenopausal (adjusted OR = 1.18; 95% CI: 1.06–1.37; *P* = 0.004) status. Regarding RR, premenopausal (adjusted RR = 1.08; 95% CI: 1.02–1.14; *P* = 0.007) and postmenopausal (adjusted RR = 1.14; 95% CI: 1.10–1.19; *P* < 0.001) had similar results.

The subgroup analysis of BC subtype OR showed that stratification of estrogen-progestin receptor status did not alter the contribution of WC to BC (adjusted OR_HR−_ = 1.44; 95% CI: 1.13–1.83; *P* = 0.003; adjusted OR_HR+_ = 1.26; 95% CI: 1.02–1.60; *P* = 0.035). Regarding RR, WC was associated with the BC development in a promotive role regardless of HR– BC (adjusted RR = 1.11; 95% CI: 0.99–1.24; *P* = 0.086) and HR+ BC (adjusted RR = 1.13; 95% CI: 1.07–1.19; *P* < 0.001).

#### Meta-analysis of WHR and BC

After pooling the results of all qualified articles, there were statistically significant associations of WHR (adjusted OR = 1.28; 1.07–1.53; *P* = 0.008) (Figure 2D) with BC. With effect values of RR, the relationship became weaker (RR = 1.05; 95% CI: 1.01–1.09; *P* = 0.017) (Figure 3D).

#### Publication bias

**Figure 7:**
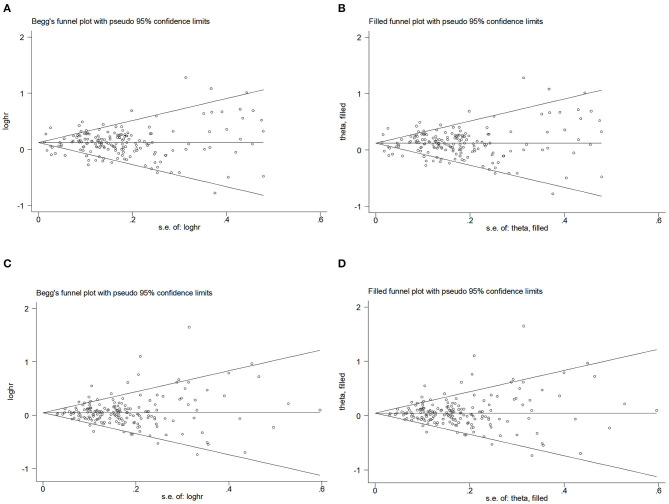
Begg's funnel plot and filled funnel plots of included studies showing odds ratios (ORs) and relative risks (RRs) for waist–hip ratio (WHR). **(A)** Begg's funnel plot, **(B)** filled funnel plot of OR, **(C)** Begg's funnel plot, and **(D)** filled funnel plot of RR.

Begg's funnel plot and Egger's test were used to assess publication bias. The results showed that Begg's funnel plots were not symmetrical in OR (Figures 7A, B) and RR (Figures 7C, D), Egger's test (*P*_OR_ = 0.156, *P*_RR_ = 0.394) showed that there was no evidence of publication bias. Egger's funnel plot showed that the study has some missing OR and RR data but no substantial change after trim-and-fill analysis.

#### Subgroup analysis

The subgroup analysis of location OR showed that there was a growing relationship between BC incidence and WHR when the regions were Asia (adjusted OR = 1.65; 95% CI: 1.07–2.55; *P* = 0.023) and Africa (adjusted OR = 1.88; 95% CI: 1.52–2.32; *P* < 0.001). No statistically significant differences were identified by RR location in any of the areas.

In the subgroup analysis of follow-up time by RR, the contribution of WHR to the BC response disappeared.

The subgroup analysis of menstrual status OR showed that WHR increased the risk of BC in premenopausal (adjusted OR = 1.38; 95% CI: 1.19–1.59; *P* < 0.001) and postmenopausal (adjusted OR = 1.41; 95% CI: 1.22–2.32; *P* < 0.001) periods. Regarding RR, the promotive effect of WHR on BC occurs in premenopausal status (adjusted RR = 1.04; 95% CI: 0.98–1.10; *P* = 0.188) and postmenopausal status (adjusted RR = 1.05; 95% CI: 1.02–1.07; *P* = 0.001).

The subgroup analysis of BC subtype OR showed that HR+ BC (adjusted OR = 1.41; 95% CI: 1.00–1.98; *P* = 0.051) and HR– BC (adjusted OR = 1.42; 95% CI: 0.95–2.13; *P* = 0.087) was associated with WHR. Both HR+ BC (adjusted RR = 1.01; 95% CI: 0.91–1.13; *P* = 0.808) and HR– BC (adjusted RR = 1.03; 95% CI: 0.98–1.07; *P* = 0.244) showed a weak relationship with WHR on BC.

#### Dose–response analysis

**Figure 8:**
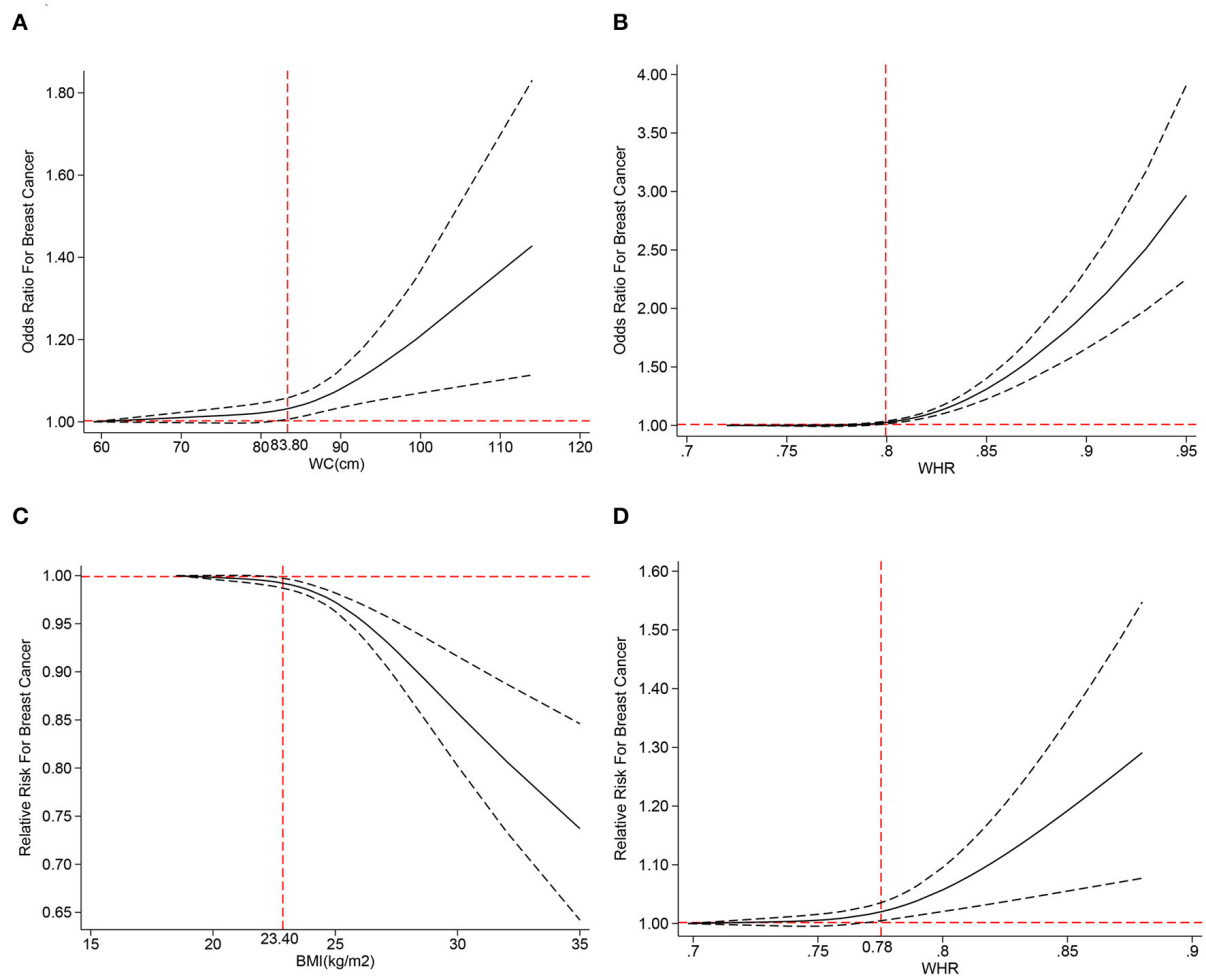
The dose–response analysis of central obesity and the risk of breast cancer for **(A)** waist circumference (WC) of odds ratios (ORs), **(B)** waist–hip ratio (WHR) of OR, **(C)** body mass index (BMI) of RR, and **(D)** waist–hip ratio (WHR) of relative risks (RRs).

We performed a dose–response analysis of the BMI, WC, HC, and WHR. Regarding OR, we only obtained J-shaped curves (Figures 8A, B) for WC and WHR. The results reflected that BC risk began to rise when WC exceeded 83.80 cm (adjusted OR = 1.03; 95% CI: 1.01–1.06) or WHR exceeded 0.80 (adjusted OR = 1.02; 95% CI: 1.01–1.03). According to RR, we obtained an inverted J-shaped curve (Figure 8C) of premenopausal BMI studies, while in WHR, we obtained a J-shaped curve (Figure 8D). The study showed that when premenopausal BMI exceeded 23.40 kg/m^2^ (adjusted RR = 0.99; 95% CI: 0.98–0.99), BC risk decreased, while WHR above 0.78 (adjusted RR = 1.02; 95% CI: 1.01–1.03) increased BC risk.

## Discussion

### Association between central obesity and BC

According to our knowledge, this is the most extensive meta-analysis exploring the dose–response relationship between central obesity and BC. We found a significant association between central obesity and BC as measured by WC and WHR in case–control and prospective cohort studies. Contrarily, HC, and BMI showed some positive relationships only in cohort studies. Moreover, sensitivity and subgroup analyses showed a weak relationship between central obesity and BC risk. The dose–response analysis showed an increased risk of BC with higher WC and WHR, while the risk decreased with higher BMI (during premenopausal).

The four measures, BMI, HC, WC, and WHR, do not have the same clinical significance. As a fat deposition marker, BMI is associated with general obesity (10). HC, measured by wrapping a tape measure around the widest area of the buttocks, represented the adipose tissue level in the buttocks (78). WC and WHR, characterized by the abdominal or visceral adipose tissue levels, are more related to the metabolic mechanism of obesity involved in BC and were used as indicators to measure central obesity (79). Our study found that BMI increases postmenopausal BC risk but decreases premenopausal risk as many studies have shown (22, 41, 47, 69); second, central obesity evaluated by WC and WHR, not HC, can predict BC. A 2015 Sister Study proved that WC and WHR are related to BC irrespective of menopausal status, validating the results of the current study. We found no evidence of a relationship between HC and BC in a combined NHS and NHSII study (58).

### Reasons for replacing HC by WC

We could elucidate anatomically the reasons for using WC instead of HC to measure central obesity. Abdominal fat mainly comprises large fat cells, whereas femur obesity is caused by an increase in normal-sized fat cells. Increasing adipose cell size decreases the insulin effect on glucose oxidation, perhaps due to a decrease in insulin receptors (80). Different glucose and insulin responses to oral glucose loading have been shown earlier in abdominal and gluteal adipocytes. Abdominal fat affects the progressive loss of glucose tolerance in surrounding tissues, leading to hyperinsulinemia and insulin insensitivity, but normal buttocks fat cells do not create this risk. Basal lipolysis rates in abdominal fat cells are greater, which compromises glucose oxidation and increases BC risk (81). This may explain why WC and WHR measure central obesity instead of HC.

### Biological plausibility

The relationship between central obesity and BC is biologically plausible. Studies have shown that excess visceral obesity tissue (VAT) has more M1 macrophages, which secrete and release pro-inflammatory cytokines and adipokines, causing insulin resistance and altered insulin initiation signaling pathways, contributing to BC (82). White adipose tissue (WAT), an adipose tissue subtype combined with central obesity, secretes many inflammatory cytokines, including TNF-α, IL-6, and IL-1β, exhibiting steroid hormone and adipokine production and chronic subclinical inflammatory changes that increase the cancer risk (83). Obese people have many senescent cells (senescent cells accumulate primarily in the viscera during obesity, creating fat deposits). Senescent cells generate a vast secretome of inflammatory chemicals, which, if persistent, can aggravate tissue damage and promote tumor growth. Therefore, obesity may promote tumor growth by inducing cellular senescence (84).

### Difference in menstrual status

According to menopausal status, our findings indicate that high WC and WHR promote BC in young women, both premenopausal and postmenopausal. Regarding premenopausal status, the finding is consistent with other prospective studies that have adjusted for BMI in premenopausal women (52, 57, 59, 62, 67, 76). The mechanisms underlying the increased central obesity and BC risk differ between premenopausal and postmenopausal. The obese and overweight groups had considerably lower estradiol levels than the normal BMI group during premenopause (85). The relationship was reversed after menopause, with the normal-weight group having the lowest estradiol levels. Studies on the effect of endogenous estrogen on BC risk in premenopausal women are rarer than in postmenopausal women and do not demonstrate a cause of premenopausal estrogen and BC risk (86, 87). We suspect that premenopausal women may be at risk due to the inflammatory reaction outlined above, not estrogen. After menopause, total estrogen decreases markedly, and most estrogen is derived from the aromatase conversion of plasma androstenedione to estrone in adipose tissue. This aromatase activity increases with body weight, raising postmenopausal plasma estrogen levels (88). In other studies, estradiol levels were consistently associated with central obesity, showing positive correlations with the WHR ratio and computed tomography-measured visceral fat area (89). Endogenously produced estrogens cause BC, and high plasma estrogen concentrations and bioavailability enhance postmenopausal BC risk (90). Thus, central adiposity may cause postmenopausal BC by increasing estrogen release. A prospective study shows that WC and WHR were more strongly associated with estrogen receptor (ER) and luminal BC in people who never took serotonin also suggests that central obesity may increase postmenopausal BC in part through estrogen levels (58).

In conclusion, central obesity promotes pre- and postmenopausal BC. A prospective investigation of central adiposity and BC risk by menopause state showed our findings (58). However, a meta-analysis by Chen et al. found that central obesity measured by WC, but not by WHR, was related to modestly increased risks of premenopausal (RR = 1.05, 95% CI: 0.99–1.10) and postmenopausal (RR = 1.06, 95% CI: 1.04–1.09) BC (91).

### Subtypes of BC

Central obesity and molecular subtypes of BC are understudied, possibly due to sample size. The sister study found no association of WC or WHR with premenopausal estrogen receptor-positive/progesterone receptor-positive (ER+/PR+) cancer and had insufficient numbers to examine estrogen receptor-negative and progesterone receptor-negative (ER–PR–) tumors (76). After BMI adjustment, HC was positively associated with premenopausal ER+/PR+ and ER–/PR– cancers in the E3N cohort. However, independent of BC molecular typing, WC and WHR did not increase BC risk (52). We pooled enough cases to find statistically significant heterogeneity in the ER/PR status of WC and WHR; with different BC molecular subtypes, central obesity can produce a consistent promoting relationship and stronger correlation in the case–control study. Central obesity is associated with elevated insulin levels, insulin-like growth factors, and reduced sex hormone-binding globulin, which may be independent of ER/PR-mediated stimulation of tumor growth. Chronic inflammation and abnormalities in visceral fat-related metabolism, such as elevated insulin-like growth factor-1 levels and hyperinsulinemia, may also contribute to this association (45).

### Sources of heterogeneity

This meta-analysis found a lot of variation across the case–control and prospective cohort studies, possibly due to methodology inconsistencies and recall and selection biases present in case–control studies. We also observed significant heterogeneity in the subgroup analyses. Two factors can cause subgroup analysis heterogeneity, first, with regional differences. Different regions have different levels of economic development, and higher prevalence rates are often found in the populations of countries with higher income levels (92) and second, exposure assessment differences. Some heterogeneity may also exist regarding different measurement methods, as in one part of the study, patients used self-reported, and the other part used instrumental measurements in hospitals.

### Comparison with previous studies

Several systematic reviews and meta-analyses have examined central obesity and breast cancer risk. A 2002 meta-analysis found that BC risk increased with waist-to-hip ratio. However, the study was limited to the relationship between WHR and BC risk and lacked other measures. Moreover, the relationship between BC molecular subtypes and central obesity has not been examined (8). Harvie showed that adjusting BMI for central obesity eliminated the link between WC or WHR and postmenopausal BC risk. However, BMI correction for central obesity was not used in subsequent meta-analyses (9). Pooling 18 prospective cohort studies by Chen showed that central obesity, as measured by WC rather than WHR, was related to a modest increase in premenopausal and postmenopausal obesity risk (10). Although the evidence is substantial, there are insufficient case–control studies to examine it. To delve deeper into the relationship, we conducted a comprehensive study with a sufficiently large sample size to assess central obesity and BC risk regarding menopausal status, tumor subtypes, and various metrics such as BMI, WC, HC, and WHR. We also observed that central obesity was associated with an increased BC risk during premenopausal and postmenopausal and verified the opposite relationship between BMI and BC risk during premenopausal and postmenopausal. However, studies involving central obesity and different BC molecular typing are lacking, and more longitudinal and well-designed studies are needed to confirm or refute our findings.

### Limitations

However, there are some limitations to our study. First, despite combining adjusted effect size estimates in the current meta-analysis, several important confounders, such as age, socioeconomic status, estrogen use, and other lifestyle factors, remained unaccounted for in all the studies. Second, participants may have undergone a pre- to postmenopausal transition during follow-up. In this study, the transition was not stratified, and menstrual status at the time of diagnosis was used as the criterion, which may have ignored the impact of premenopausal hormone levels on body shape. Third, our data suggested that central obesity may raise the risk of different molecular subtypes of BC, although further longitudinal studies are needed to validate this. Finally, as with all meta-analyses, this study was limited by the quality of the included studies. Observational studies are prone to selection bias, recall bias, and exaggerated associations. Interpreting observational studies is difficult, specifically since the studies included in our meta-analysis were rated at moderate risk of bias, mainly because of severe bias due to confounding confounders. Confounding bias may affect the validity of the associations we observed. We hope that the quality of the evidence will improve with future updates and more high-quality research.

## Conclusion

This dose analysis showed that central obesity, as WC and WHR measured, was associated with premenopausal and postmenopausal BC risk and ER+/ER– BC risk. Our study suggests that women should prioritize body type management to prevent BC.

## Data availability statement

The original contributions presented in the study are included in the article/Supplementary material, further inquiries can be directed to the corresponding author.

## Author contributions

DW, GY, and CA conceived and designed the experiments. MY, HC, and XQ performed the experiments. MY and HC analyzed the data. HC, XQ, DC, FL, JY, CZ, and YN contributed materials and analysis tools. HC, MY, and DW wrote and revised the article. All authors read and approved the final manuscript before submission.
