# Supplementary material for: The relationship between central obesity and risk of breast cancer: a dose–response meta-analysis of 7,989,315 women
Source: Front Nutr. 2023 Nov 9;10:1236393. doi: 10.3389/fnut.2023.1236393 (PMC10665573; doi:10.3389/fnut.2023.1236393)
Supplement: Supplementary file 1 [file Table_1.DOCX]

**Supplementary Table 1. PRISMA checklist of this Meta-analysis.**

Title: The Relationship Between Central Obesity and Risk of Breast Cancer: a Dose–Response Meta‑analysis of 7,989,315 Women

Question: Central obesity may increase the risk of breast cancer, which is proven. But there is a lack of dose-response analysis, to evaluate the relationship between Central obesity and risk of breast cancer, Including premenopausal, postmenopausal and hormone receptor types. And these questions remain unknown.

**18. * Condition or domain being studied.**

Breast cancer has surpassed lung cancer as the most commonly diagnosed cancer, with an estimated 2.3 million new cases, which accounted for approximately 11.7% % of all cancer. Given this disease burden, identifying potentially modifiable factors associated with BC development is of particular public health significance. Specifically, central obesity is such a factor that has been consistently shown to be associated with increased risk of BC. But the dose-response relationship between them remains unknown. So in this meta-analysis,we aim to prove the relationship between central obesity and risk of breast cancer and to do a dose-response analysis.

1. *** Participants/population.**

**Women who were diagnosed with breast cancer .**

**20. * Intervention(s), exposure(s).**

Obesity is a chronic disease characterised by an increase of body fat stores. In clinical practice, the body fatness is usually estimated by BMI. BMI is calculated as measured body weight (kg) divided by measured height squared (m^2^). In adults (age over 18 years) obesity is defined by a BMI 30 kg/m^2^ and overweight (also termed pre-obesity) by a BMI between 25 and 29.9 kg/m2. The amount of abdominal fat can be assessed by waist circumference (WC) and waist-to-hip ratio (WHR) which highly correlates with intra-abdominal fat content. Central obesity is defined by the World Health Organization defined central obesity as a (WC) of greater than 80 cm, or the WHR is more than 0.85 for females.

**21. * Comparator(s)/control.**

Women without breast cancer.

**22. * Types of study to be included**

Case-control studies and prospective cohort studies.

**24. * Main outcome(s).**

The main outcome of our research was the impact of central obesity on the risk of breast cancer.

Measures of effect

Odds Ratio and Risk Ratio were used as the effect measure for our main outcome.

**25. * Additional outcome(s).**

The effect of measured value of central obesity on the risk of breast cancer will be demonstrated as a dose-response analysis result.

**26. * Data extraction (selection and coding).**

For the studies fulfilled our inclusion criteria, two investigators will independently extract data including the following items: first author, year of publication, country where study was conducted, sample size, study type, age of the population, menstrual status, measurement index, hormone receptor typing.

**27. * Risk of bias (quality) assessment.**

Both case-control and prospective cohort studies will be assessed by the Newcastle-Ottawa Scale (NOS) tool to evaluate the risk of bias.

**28. * Strategy for data synthesis.**

1. STATA software version 14.1 for Windows (Stata Corp, College Station, TX, USA) was applied to handle the data management and analysis. Effect size was estimated as odds ratio (OR) and risk ratio (RR) with 95% confidence interval (CI) .

2.The dose-response association for trend estimation of summarized dose-response data was examined by Generalized least squares regression proposed by Greenland and Longnecker.

3.Pooled effect-size estimates were derived under the random-effects model, irrespective of the magnitude of between-study heterogeneity.

4.The inconsistency index (I²) statistic, a kind of index to represent the percent of diversity that is due to heterogeneity rather than chance, was applied to quantify the magnitude of heterogeneity derived from the random-effects Mantel-Haenszel model. It indicated the significant heterogeneity if the I² is greater than 50%.

5. Begg’s funnel plots and Egger regression asymmetry tests was operated to evaluate the probability of publication bias at a significance level of 10%. The trim-and-fill method was utilized to estimate the number of theoretically missing studies. Sensitivity analysis was performed to test the reliability of the total pooled results by omitting the study sequentially.

6. To state the possible sources of between-study heterogeneity from clinical and methodological aspects, an amount of pre-specified subgroup analyses will be done.

**29. * Analysis of subgroups or subsets.**

The subgroup analysis will be conducted according to geographical area, age, menstrual status, measurement index, molecular classification, follow-up time and type of study.

Systematic review registration：CRD42022365788
