# Supplementary material for: The relationship between central obesity and risk of breast cancer: a dose–response meta-analysis of 7,989,315 women
Source: Front Nutr. 2023 Nov 9;10:1236393. doi: 10.3389/fnut.2023.1236393 (PMC10665573; doi:10.3389/fnut.2023.1236393)
Supplement: Supplementary file 2 [file Table_2.DOCX]

**Supplementary Table 2. Search terms of this meta-analysis.**

| **Search Terms** |
| --- |
| Based on PubMed, Medline, Embase, and Web of Science. |
| (breast carcinoma OR breast tumor OR breast cancer OR breast neoplasms)[Title/Abstract] AND (adipose tissue OR fat distribution OR body size OR central obesities OR central adiposity OR abdominal obesity OR abdominal obesities OR central fatness OR abdominal adiposity OR visceral obesity OR visceral obesities OR visceral adiposity OR abdominal fat OR abdominal adipose tissue OR waist hip ratio OR waist-hip ratios OR waist-to-hip ratio OR waist to hip ratio OR WHR OR waistline OR waist circumference OR somatotype OR body type OR body build OR body shape OR mesomorph )[Title/Abstract] AND ( risk OR cohort OR incidence OR concurrent OR nested)[Title/Abstract] |
