# Supplementary material for: The relationship between central obesity and risk of breast cancer: a dose–response meta-analysis of 7,989,315 women
Source: Front Nutr. 2023 Nov 9;10:1236393. doi: 10.3389/fnut.2023.1236393 (PMC10665573; doi:10.3389/fnut.2023.1236393)
Supplement: Supplementary file 3 [file Table_3.docx]

**Supplementary table 3: List of excluded studies after full text screening**

| S. No. | Study reference | Reasons for exclusion |
| --- | --- | --- |
|  | Sellers TA, Kushi LH, Potter JD, Kaye SA, Nelson CL, McGovern PG, et al. Effect of family history, body-fat distribution, and reproductive factors on the risk of postmenopausal breast cancer. The New England journal of medicine. 1992;326(20):1323-9. | Wrong patient population |
|  | Sellers TA, Gapstur SM, Potter JD, Kushi LH, Bostick RM, Folsom AR. Association of body fat distribution and family histories of breast and ovarian cancer with risk of postmenopausal breast cancer. American journal of epidemiology. 1993;138(10):799-803. | Wrong patient population |
|  | Barnes-Josiah D, Potter JD, Sellers TA, Himes JH. Early body size and subsequent weight gain as predictors of breast cancer incidence (Iowa, United States). Cancer causes & control : CCC. 1995;6(2):112-8. | Wrong outcomes |
|  | Ballard-Barbash R, Swanson CA. Body weight: estimation of risk for breast and endometrial cancers. The American journal of clinical nutrition. 1996;63(3 Suppl):437s-41s. | Wrong outcomes |
|  | Folsom AR, Kushi LH, Anderson KE, Mink PJ, Olson JE, Hong CP, et al. Associations of general and abdominal obesity with multiple health outcomes in older women: the Iowa Women's Health Study. Archives of internal medicine. 2000;160(14):2117-28. | Wrong outcomes |
|  | Hall IJ, Newman B, Millikan RC, Moorman PG. Body size and breast cancer risk in black women and white women: the Carolina Breast Cancer Study. American journal of epidemiology. 2000;151(8):754-64. | Wrong patient population |
|  | Morimoto LM, White E, Chen Z, Chlebowski RT, Hays J, Kuller L, et al. Obesity, body size, and risk of postmenopausal breast cancer: the Women's Health Initiative (United States). Cancer causes & control : CCC. 2002;13(8):741-51. | Wrong patient population |
|  | McCormack VA, Mangtani P, Bhakta D, McMichael AJ, dos Santos Silva I. Heterogeneity of breast cancer risk within the South Asian female population in England: a population-based case-control study of first-generation migrants. British journal of cancer. 2004;90(1):160-6. | Wrong patient population |
|  | Kumar NB, Riccardi D, Cantor A, Dalton K, Allen K. A case-control study evaluating the association of purposeful physical activity, body fat distribution, and steroid hormones on premenopausal breast cancer risk. The breast journal. 2005;11(4):266-72. | Wrong outcomes |
|  | Han D, Nie J, Bonner MR, McCann SE, Muti P, Trevisan M, et al. Lifetime adult weight gain, central adiposity, and the risk of pre- and postmenopausal breast cancer in the Western New York exposures and breast cancer study. International journal of cancer. 2006;119(12):2931-7. | Wrong outcomes |
|  | Li HL, Gao YT, Li Q, Liu DK. [Anthropometry and female breast cancer: a prospective cohort study in urban Shanghai]. Zhonghua liu xing bing xue za zhi = Zhonghua liuxingbingxue zazhi. 2006;27(6):488-93. | Wrong outcomes |
|  | Rinaldi S, Key TJ, Peeters PH, Lahmann PH, Lukanova A, Dossus L, et al. Anthropometric measures, endogenous sex steroids and breast cancer risk in postmenopausal women: a study within the EPIC cohort. International journal of cancer. 2006;118(11):2832-9. | Wrong outcomes |
|  | Ahn J, Schatzkin A, Lacey JV, Jr., Albanes D, Ballard-Barbash R, Adams KF, et al. Adiposity, adult weight change, and postmenopausal breast cancer risk. Archives of internal medicine. 2007;167(19):2091-102. | Wrong outcomes |
|  | Slattery ML, Sweeney C, Edwards S, Herrick J, Baumgartner K, Wolff R, et al. Body size, weight change, fat distribution and breast cancer risk in Hispanic and non-Hispanic white women. Breast cancer research and treatment. 2007;102(1):85-101. | Wrong patient population |
|  | Kabat GC, Kim M, Chlebowski RT, Khandekar J, Ko MG, McTiernan A, et al. A longitudinal study of the metabolic syndrome and risk of postmenopausal breast cancer. Cancer epidemiology, biomarkers & prevention : a publication of the American Association for Cancer Research, cosponsored by the American Society of Preventive Oncology. 2009;18(7):2046-53. | Wrong outcomes |
|  | Nemesure B, Wu SY, Hennis A, Leske MC. Body size and breast cancer in a black population--the Barbados National Cancer Study. Cancer causes & control : CCC. 2009;20(3):387-94. | Wrong outcomes |
|  | Hajian-Tilaki KO, Gholizadehpasha AR, Bozorgzadeh S, Hajian-Tilaki E. Body mass index and waist circumference are predictor biomarkers of breast cancer risk in Iranian women. Medical oncology (Northwood, London, England). 2011;28(4):1296-301. | Wrong outcomes |
|  | Inamdar P, Mehta G. Correlation Between Obesity and High Density Lipoprotein Cholesterol (HDL-C) in Breast Cancer Patients of Southern Rajasthan. Indian journal of surgical oncology. 2011;2(2):118-21. | Wrong outcomes |
|  | Luo J, Horn K, Ockene JK, Simon MS, Stefanick ML, Tong E, et al. Interaction between smoking and obesity and the risk of developing breast cancer among postmenopausal women: the Women's Health Initiative Observational Study. American journal of epidemiology. 2011;174(8):919-28. | Wrong patient population |
|  | Lynch BM, Friedenreich CM, Winkler EA, Healy GN, Vallance JK, Eakin EG, et al. Associations of objectively assessed physical activity and sedentary time with biomarkers of breast cancer risk in postmenopausal women: findings from NHANES (2003-2006). Breast cancer research and treatment. 2011;130(1):183-94. | Wrong intervention |
|  | Rosato V, Bosetti C, Talamini R, Levi F, Montella M, Giacosa A, et al. Metabolic syndrome and the risk of breast cancer in postmenopausal women. Annals of oncology : official journal of the European Society for Medical Oncology. 2011;22(12):2687-92. | Wrong outcomes |
|  | Hartz A, He T, Rimm A. Comparison of adiposity measures as risk factors in postmenopausal women. The Journal of clinical endocrinology and metabolism. 2012;97(1):227-33. | Wrong patient population |
|  | White KK, Park SY, Kolonel LN, Henderson BE, Wilkens LR. Body size and breast cancer risk: the Multiethnic Cohort. International journal of cancer. 2012;131(5):E705-16. | Wrong intervention; Wrong patient population |
|  | Bandera EV, Chandran U, Zirpoli G, Gong Z, McCann SE, Hong CC, et al. Body fatness and breast cancer risk in women of African ancestry. BMC cancer. 2013;13:475. | Wrong patient population |
|  | John EM, Phipps AI, Sangaramoorthy M. Body size, modifying factors, and postmenopausal breast cancer risk in a multiethnic population: the San Francisco Bay Area Breast Cancer Study. SpringerPlus. 2013;2(1):239. | Wrong patient population |
|  | van Kruijsdijk RC, van der Graaf Y, Peeters PH, Visseren FL. Cancer risk in patients with manifest vascular disease: effects of smoking, obesity, and metabolic syndrome. Cancer epidemiology, biomarkers & prevention : a publication of the American Association for Cancer Research, cosponsored by the American Society of Preventive Oncology. 2013;22(7):1267-77. | Wrong patient population |
|  | Zagami SE, Golmakani N, Shandiz FH, Saki A. Evaluating the Relationship between Body Size and Body Shape with the Risk of Breast Cancer. Oman medical journal. 2013;28(6):389-94. | Wrong outcomes |
|  | Fourkala EO, Burnell M, Cox C, Ryan A, Salter LC, Gentry-Maharaj A, et al. Association of skirt size and postmenopausal breast cancer risk in older women: a cohort study within the UK Collaborative Trial of Ovarian Cancer Screening (UKCTOCS). BMJ open. 2014;4(9):e005400. | Wrong outcomes |
|  | Robinson WR, Tse CK, Olshan AF, Troester MA. Body size across the life course and risk of premenopausal and postmenopausal breast cancer in Black women, the Carolina Breast Cancer Study, 1993-2001. Cancer causes & control : CCC. 2014;25(9):1101-17. | Wrong patient population |
|  | John EM, Sangaramoorthy M, Hines LM, Stern MC, Baumgartner KB, Giuliano AR, et al. Overall and abdominal adiposity and premenopausal breast cancer risk among hispanic women: the breast cancer health disparities study. Cancer epidemiology, biomarkers & prevention : a publication of the American Association for Cancer Research, cosponsored by the American Society of Preventive Oncology. 2015;24(1):138-47. | Wrong patient population |
|  | John EM, Sangaramoorthy M, Hines LM, Stern MC, Baumgartner KB, Giuliano AR, et al. Body size throughout adult life influences postmenopausal breast cancer risk among hispanic women: the breast cancer health disparities study. Cancer epidemiology, biomarkers & prevention : a publication of the American Association for Cancer Research, cosponsored by the American Society of Preventive Oncology. 2015;24(1):128-37. | Wrong patient population; Wrong comparison;Dupliction |
|  | Kabat GC, Xue X, Kamensky V, Lane D, Bea JW, Chen C, et al. Risk of breast, endometrial, colorectal, and renal cancers in postmenopausal women in association with a body shape index and other anthropometric measures. Cancer causes & control : CCC. 2015;26(2):219-29. | Wrong outcomes |
|  | Chollet-Hinton L, Anders CK, Tse CK, Bell MB, Yang YC, Carey LA, et al. Breast cancer biologic and etiologic heterogeneity by young age and menopausal status in the Carolina Breast Cancer Study: a case-control study. Breast cancer research : BCR. 2016;18(1):79. | Wrong patient population |
|  | Horn-Ross PL, Canchola AJ, Bernstein L, Neuhausen SL, Nelson DO, Reynolds P. Lifetime body size and estrogen-receptor-positive breast cancer risk in the California Teachers Study cohort. Breast cancer research : BCR. 2016;18(1):132. | Wrong intervention |
|  | Wu AH, Vigen C, Lee E, Tseng CC, Butler LM. Traditional Breast Cancer Risk Factors in Filipina Americans Compared with Chinese and Japanese Americans in Los Angeles County. Cancer epidemiology, biomarkers & prevention : a publication of the American Association for Cancer Research, cosponsored by the American Society of Preventive Oncology. 2016;25(12):1572-86. | Wrong patient population |
|  | Chollet-Hinton L, Olshan AF, Nichols HB, Anders CK, Lund JL, Allott EH, et al. Biology and Etiology of Young-Onset Breast Cancers among Premenopausal African American Women: Results from the AMBER Consortium. Cancer epidemiology, biomarkers & prevention : a publication of the American Association for Cancer Research, cosponsored by the American Society of Preventive Oncology. 2017;26(12):1722-9. | Wrong patient population |
|  | Kabat GC, Kim MY, Lee JS, Ho GY, Going SB, Beebe-Dimmer J, et al. Metabolic Obesity Phenotypes and Risk of Breast Cancer in Postmenopausal Women. Cancer epidemiology, biomarkers & prevention : a publication of the American Association for Cancer Research, cosponsored by the American Society of Preventive Oncology. 2017;26(12):1730-5. | Wrong outcomes |
|  | Liu LY, Wang F, Cui SD, Tian FG, Fan ZM, Geng CZ, et al. A case-control study on risk factors of breast cancer in Han Chinese women. Oncotarget. 2017;8(57):97217-30. | Wrong outcomes |
|  | Park YM, White AJ, Nichols HB, O'Brien KM, Weinberg CR, Sandler DP. The association between metabolic health, obesity phenotype and the risk of breast cancer. International journal of cancer. 2017;140(12):2657-66. | Wrong outcomes |
|  | Akalanka HMK, Ekanayake S, Samarasinghe K. Could Anthropometric and Lipid Parameters Reflect Susceptibility to Breast Cancer? Comparison of Newly Diagnosed Breast Cancer and Apparently Healthy Women. Asian Pacific journal of cancer prevention : APJCP. 2018;19(9):2475-80. | Wrong comparison |
|  | Antony MP, Surakutty B, Vasu TA, Chisthi M. Risk factors for breast cancer among Indian women: A case-control study. Nigerian journal of clinical practice. 2018;21(4):436-42. | Wrong comparison |
|  | Godinho-Mota JCM, Martins KA, Vaz-Gonçalves L, Mota JF, Soares LR, Freitas-Junior R. Visceral adiposity increases the risk of breast cancer: a case-control study. Nutricion hospitalaria. 2018;35(3):576-81. | Wrong outcomes |
|  | Chen F, Zhu Z, van Duijnhoven FJB, Dong M, Qian Y, Yu H, et al. Genetic Variants in Group-Specific Component (GC) Gene Are Associated with Breast Cancer Risk among Chinese Women. BioMed research international. 2019;2019:3295781. | Wrong outcomes |
|  | Asaduzzaman M, Zannat IA, Akhtar PS, Shahi A, Sarker F, Islam MR, et al. Relation of Obesity with Breast Cancer among the Patients Attending at National Institute of Cancer Research & Hospital. Mymensingh medical journal : MMJ. 2020;29(3):676-83. | Not accepted |
|  | Park B, Kim S, Kim H, Cha C, Chung MS. Associations between obesity, metabolic health, and the risk of breast cancer in East Asian women. British journal of cancer. 2021;125(12):1718-25. | Wrong outcomes |
|  | Park IS, Kim SI, Han Y, Yoo J, Seol A, Jo H, et al. Risk of female-specific cancers according to obesity and menopausal status in 2•7 million Korean women: Similar trends between Korean and Western women. The Lancet regional health Western Pacific. 2021;11:100146. | Wrong outcomes |
|  | Parra-Soto S, Cowley ES, Rezende LFM, Ferreccio C, Mathers JC, Pell JP, et al. Associations of six adiposity-related markers with incidence and mortality from 24 cancers-findings from the UK Biobank prospective cohort study. BMC medicine. 2021;19(1):7. | Wrong outcomes |
|  | Swerdlow AJ, Bruce C, Cooke R, Coulson P, Griffin J, Butlin A, et al. Obesity and Breast Cancer Risk in Men: A National Case-Control Study in England and Wales. JNCI cancer spectrum. 2021;5(5). | Not accepted |
|  | Brantley KD, Zeleznik OA, Dickerman BA, Balasubramanian R, Clish CB, Avila-Pacheco J, et al. A metabolomic analysis of adiposity measures and pre- and postmenopausal breast cancer risk in the Nurses' Health Studies. British journal of cancer. 2022. | Wrong outcomes |
|  | Hao Y, Xiao J, Liang Y, Wu X, Zhang H, Xiao C, et al. Reassessing the causal role of obesity in breast cancer susceptibility: a comprehensive multivariable Mendelian randomization investigating the distribution and timing of exposure. International journal of epidemiology. 2022. | Wrong study design |
|  | Park B. Changes in weight and waist circumference during menopausal transition and postmenopausal breast cancer risk. International journal of cancer. 2022;150(9):1431-8. | Unavailable for the articles |
|  | Swerdlow AJ, Bruce C, Cooke R, Coulson P, Schoemaker MJ, Jones ME. Risk of breast cancer in men in relation to weight change: A national case-control study in England and Wales. International journal of cancer. 2022;150(11):1804-11. | Wrong patient population |
|  | den Tonkelaar I, Seidell JC, Collette HJ. Body fat distribution in relation to breast cancer in women participating in the DOM-project. Breast cancer research and treatment. 1995;34(1):55-61. | Wrong outcomes |
|  | Bruning PF, Bonfrèr JM, Hart AA, van Noord PA, van der Hoeven H, Collette HJ, et al. Body measurements, estrogen availability and the risk of human breast cancer: a case-control study. International journal of cancer. 1992;51(1):14-9. | Wrong outcomes |
