# Supplementary material for: The relationship between central obesity and risk of breast cancer: a dose–response meta-analysis of 7,989,315 women
Source: Front Nutr. 2023 Nov 9;10:1236393. doi: 10.3389/fnut.2023.1236393 (PMC10665573; doi:10.3389/fnut.2023.1236393)
Supplement: Supplementary file 4 [file Table_4.DOCX]

Supplementary Table 4. Baseline characteristics of the studies included in this meta-analysis.

| **Year** | **Author** | **Location** | **Study type** | **age（years）** | **No.case** | **No.total** | **Exposure assessment** | **Follow up（years）** | **Menopause status** | **Exposure** | **Exposure range** | **Subtype of BC** | **RR (95%CI)** | **Adjustment** |
| --- | --- | --- | --- | --- | --- | --- | --- | --- | --- | --- | --- | --- | --- | --- |
| 1990 | Folsom | USA | Prospective cohort studies | 55-69 | 229 | 41,837 | Self reported | 2.00 | Postmenopause | BMI | < 24.40 | NA | 1.00 | Age at menarche, late age at first live birth, educational level, use of estrogens, and family history of BC. |
| 1990 | Folsom | USA | Prospective cohort studies | 55-69 | 229 | 41,837 | Self reported | 2.00 | Postmenopause | BMI | 24.40- 28.35 | NA | 0.89 (0.65-1.22) |  |
| 1990 | Folsom | USA | Prospective cohort studies | 55-69 | 229 | 41,837 | Self reported | 2.00 | Postmenopause | BMI | > 28.35 | NA | 1.05 (0.78-1.41) |  |
| 1990 | Folsom | USA | Prospective cohort studies | 55-69 | 229 | 41,837 | Self reported | 2.00 | Postmenopause | WC | <80 | NA | 1.00 |  |
| 1990 | Folsom | USA | Prospective cohort studies | 55-69 | 229 | 41,837 | Self reported | 2.00 | Postmenopause | WC | 80-92.96 | NA | 1.25 (0.92-1.67) |  |
| 1990 | Folsom | USA | Prospective cohort studies | 55-69 | 229 | 41,837 | Self reported | 2.00 | Postmenopause | WC | >92.96 | NA | 1.05 (0.75-1.42) |  |
| 1990 | Folsom | USA | Prospective cohort studies | 55-69 | 229 | 41,837 | Self reported | 2.00 | Postmenopause | WHR | <0.79 | NA | 1.00 |  |
| 1990 | Folsom | USA | Prospective cohort studies | 55-69 | 229 | 41,837 | Self reported | 2.00 | Postmenopause | WHR | 0.79-0.87 | NA | 0.94 (0.67-1.30) |  |
| 1990 | Folsom | USA | Prospective cohort studies | 55-69 | 229 | 41,837 | Self reported | 2.00 | Postmenopause | WHR | >0.87 | NA | 1.34 (0.99-1.79) |  |
| 1990 | Schapira | USA | Prospective cohort studies | 25-83 | 216 | 648 | Technicians measured | NA | NA | WHR | < 0.73 | NA | 1.00 | Abdomen, age, triceps skinfold, fibrocystic disease, height, percentage fat, weight, BMI, and WC. |
| 1990 | Schapira | USA | Prospective cohort studies | 25-83 | 216 | 648 | Technicians measured | NA | NA | WHR | 0.73 -0.76 | NA | 1.73 (1.41-2.13) |  |
| 1990 | Schapira | USA | Prospective cohort studies | 25-83 | 216 | 648 | Technicians measured | NA | NA | WHR | 0.77 - 0.80 | NA | 3.01 (1.99-4.53) |  |
| 1990 | Schapira | USA | Prospective cohort studies | 25-83 | 216 | 648 | Technicians measured | NA | NA | WHR | > 0.81 | NA | 5.21 (2.81-9.65) |  |
| 1998 | Kaaks | The Netherlands | Prospective cohort studies | 39-73 | 275 | 11,663 | Technicians measured | 10.60 | Premenopause | BMI | ≤22.5 | NA | 1.00 | Age, age at menarche, age at first birth, age at menopause, and parity. |
| 1998 | Kaaks | The Netherlands | Prospective cohort studies | 39-73 | 275 | 11,663 | Technicians measured | 10.60 | Premenopause | BMI | 22.50–24.54 | NA | 1.08 (0.71-1.64) |  |
| 1998 | Kaaks | The Netherlands | Prospective cohort studies | 39-73 | 275 | 11,663 | Technicians measured | 10.60 | Premenopause | BMI | 24.54–27.15 | NA | 0.76 (0.47-1.24) |  |
| 1998 | Kaaks | The Netherlands | Prospective cohort studies | 39-73 | 275 | 11,663 | Technicians measured | 10.60 | Premenopause | BMI | >27.15 | NA | 1.04 (0.65-1.68) |  |
| 1998 | Kaaks | The Netherlands | Prospective cohort studies | 39-73 | 275 | 11,663 | Technicians measured | 10.60 | Premenopause | WC | ≤71 | NA | 1.00 |  |
| 1998 | Kaaks | The Netherlands | Prospective cohort studies | 39-73 | 275 | 11,663 | Technicians measured | 10.60 | Premenopause | WC | 71-77 | NA | 0.97 (0.65-1.45) |  |
| 1998 | Kaaks | The Netherlands | Prospective cohort studies | 39-73 | 275 | 11,663 | Technicians measured | 10.60 | Premenopause | WC | 77-83.5 | NA | 0.73 (0.44-1.19) |  |
| 1998 | Kaaks | The Netherlands | Prospective cohort studies | 39-73 | 275 | 11,663 | Technicians measured | 10.60 | Premenopause | WC | >83.5 | NA | 0.92 (0.57-1.50) |  |
| 1998 | Kaaks | The Netherlands | Prospective cohort studies | 39-73 | 275 | 11,663 | Technicians measured | 10.60 | Premenopause | HC | ≤96 | NA | 1.00 |  |
| 1998 | Kaaks | The Netherlands | Prospective cohort studies | 39-73 | 275 | 11,663 | Technicians measured | 10.60 | Premenopause | HC | 96-101 | NA | 0.64 (0.41-0.99) |  |
| 1998 | Kaaks | The Netherlands | Prospective cohort studies | 39-73 | 275 | 11,663 | Technicians measured | 10.60 | Premenopause | HC | 101-106 | NA | 0.95 (0.62-1.46) |  |
| 1998 | Kaaks | The Netherlands | Prospective cohort studies | 39-73 | 275 | 11,663 | Technicians measured | 10.60 | Premenopause | HC | >106 | NA | 0.69 (0.43-1.12) |  |
| 1998 | Kaaks | The Netherlands | Prospective cohort studies | 39-73 | 275 | 11,663 | Technicians measured | 10.60 | Premenopause | WHR | ≤0.73 | NA | 1.00 |  |
| 1998 | Kaaks | The Netherlands | Prospective cohort studies | 39-73 | 275 | 11,663 | Technicians measured | 10.60 | Premenopause | WHR | 0.73-0.76 | NA | 0.99 (0.64-1.56) |  |
| 1998 | Kaaks | The Netherlands | Prospective cohort studies | 39-73 | 275 | 11,663 | Technicians measured | 10.60 | Premenopause | WHR | 0.76-0.80 | NA | 1.07 (0.69-1.64) |  |
| 1998 | Kaaks | The Netherlands | Prospective cohort studies | 39-73 | 275 | 11,663 | Technicians measured | 10.60 | Premenopause | WHR | >0.80 | NA | 0.96 (0.60-1.54) |  |
| 1998 | Kaaks | The Netherlands | Prospective cohort studies | 39-73 | 275 | 11,663 | Technicians measured | 10.60 | Postmenopause | BMI | ≤22.5 | NA | 1.00 |  |
| 1998 | Kaaks | The Netherlands | Prospective cohort studies | 39-73 | 275 | 11,663 | Technicians measured | 10.60 | Postmenopause | BMI | 22.50–24.54 | NA | 0.69 (0.34-1.40) |  |
| 1998 | Kaaks | The Netherlands | Prospective cohort studies | 39-73 | 275 | 11,663 | Technicians measured | 10.60 | Postmenopause | BMI | 24.54–27.15 | NA | 0.49 (0.24-1.00) |  |
| 1998 | Kaaks | The Netherlands | Prospective cohort studies | 39-73 | 275 | 11,663 | Technicians measured | 10.60 | Postmenopause | BMI | >27.15 | NA | 0.81 (0.43-1.51) |  |
| 1998 | Kaaks | The Netherlands | Prospective cohort studies | 39-73 | 275 | 11,663 | Technicians measured | 10.60 | Postmenopause | WC | ≤71 | NA | 1.00 |  |
| 1998 | Kaaks | The Netherlands | Prospective cohort studies | 39-73 | 275 | 11,663 | Technicians measured | 10.60 | Postmenopause | WC | 71-77 | NA | 1.68 (0.66-4.25) |  |
| 1998 | Kaaks | The Netherlands | Prospective cohort studies | 39-73 | 275 | 11,663 | Technicians measured | 10.60 | Postmenopause | WC | 77-83.5 | NA | 1.38 (0.54-3.53) |  |
| 1998 | Kaaks | The Netherlands | Prospective cohort studies | 39-73 | 275 | 11,663 | Technicians measured | 10.60 | Postmenopause | WC | >83.5 | NA | 1.99 (0.81-4.86) |  |
| 1998 | Kaaks | The Netherlands | Prospective cohort studies | 39-73 | 275 | 11,663 | Technicians measured | 10.60 | Postmenopause | HC | ≤96 | NA | 1.00 |  |
| 1998 | Kaaks | The Netherlands | Prospective cohort studies | 39-73 | 275 | 11,663 | Technicians measured | 10.60 | Postmenopause | HC | 96-101 | NA | 1.09 (0.52-2.27) |  |
| 1998 | Kaaks | The Netherlands | Prospective cohort studies | 39-73 | 275 | 11,663 | Technicians measured | 10.60 | Postmenopause | HC | 101-106 | NA | 1.10 (0.53-2.26) |  |
| 1998 | Kaaks | The Netherlands | Prospective cohort studies | 39-73 | 275 | 11,663 | Technicians measured | 10.60 | Postmenopause | HC | >106 | NA | 1.17 (0.58-2.35) |  |
| 1998 | Kaaks | The Netherlands | Prospective cohort studies | 39-73 | 275 | 11,663 | Technicians measured | 10.60 | Postmenopause | WHR | ≤0.73 | NA | 1.00 |  |
| 1998 | Kaaks | The Netherlands | Prospective cohort studies | 39-73 | 275 | 11,663 | Technicians measured | 10.60 | Postmenopause | WHR | 0.73-0.76 | NA | 1.24 (0.44-3.49) |  |
| 1998 | Kaaks | The Netherlands | Prospective cohort studies | 39-73 | 275 | 11,663 | Technicians measured | 10.60 | Postmenopause | WHR | 0.76-0.80 | NA | 2.06 (0.83-5.13) |  |
| 1998 | Kaaks | The Netherlands | Prospective cohort studies | 39-73 | 275 | 11,663 | Technicians measured | 10.60 | Postmenopause | WHR | >0.80 | NA | 2.63 (1.09-6.35) |  |
| 1998 | Kaaks | The Netherlands | Prospective cohort studies | 39-73 | 275 | 11,663 | Technicians measured | 10.60 | Hysterectomy/ovariectomy | BMI | ≤22.5 | NA | 1.00 |  |
| 1998 | Kaaks | The Netherlands | Prospective cohort studies | 39-73 | 275 | 11,663 | Technicians measured | 10.60 | Hysterectomy/ovariectomy | BMI | 22.50–24.54 | NA | 1.47 (0.58-3.70) |  |
| 1998 | Kaaks | The Netherlands | Prospective cohort studies | 39-73 | 275 | 11,663 | Technicians measured | 10.60 | Hysterectomy/ovariectomy | BMI | 24.54–27.15 | NA | 1.57 (0.63-3.92) |  |
| 1998 | Kaaks | The Netherlands | Prospective cohort studies | 39-73 | 275 | 11,663 | Technicians measured | 10.60 | Hysterectomy/ovariectomy | BMI | >27.15 | NA | 1.50 (0.61-3.70) |  |
| 1998 | Kaaks | The Netherlands | Prospective cohort studies | 39-73 | 275 | 11,663 | Technicians measured | 10.60 | Hysterectomy/ovariectomy | WC | ≤71 | NA | 1.00 |  |
| 1998 | Kaaks | The Netherlands | Prospective cohort studies | 39-73 | 275 | 11,663 | Technicians measured | 10.60 | Hysterectomy/ovariectomy | WC | 71-77 | NA | 0.62 (0.24-1.57) |  |
| 1998 | Kaaks | The Netherlands | Prospective cohort studies | 39-73 | 275 | 11,663 | Technicians measured | 10.60 | Hysterectomy/ovariectomy | WC | 77-83.5 | NA | 1.43 (0.65-3.13) |  |
| 1998 | Kaaks | The Netherlands | Prospective cohort studies | 39-73 | 275 | 11,663 | Technicians measured | 10.60 | Hysterectomy/ovariectomy | WC | >83.5 | NA | 0.95 (0.42-2.18) |  |
| 1998 | Kaaks | The Netherlands | Prospective cohort studies | 39-73 | 275 | 11,663 | Technicians measured | 10.60 | Hysterectomy/ovariectomy | HC | ≤96 | NA | 1.00 |  |
| 1998 | Kaaks | The Netherlands | Prospective cohort studies | 39-73 | 275 | 11,663 | Technicians measured | 10.60 | Hysterectomy/ovariectomy | HC | 96-101 | NA | 0.69 (0.29-1.65) |  |
| 1998 | Kaaks | The Netherlands | Prospective cohort studies | 39-73 | 275 | 11,663 | Technicians measured | 10.60 | Hysterectomy/ovariectomy | HC | 101-106 | NA | 1.15 (0.52-2.53) |  |
| 1998 | Kaaks | The Netherlands | Prospective cohort studies | 39-73 | 275 | 11,663 | Technicians measured | 10.60 | Hysterectomy/ovariectomy | HC | >106 | NA | 1.15 (0.54-2.45) |  |
| 1998 | Kaaks | The Netherlands | Prospective cohort studies | 39-73 | 275 | 11,663 | Technicians measured | 10.60 | Hysterectomy/ovariectomy | WHR | ≤0.73 | NA | 1.00 |  |
| 1998 | Kaaks | The Netherlands | Prospective cohort studies | 39-73 | 275 | 11,663 | Technicians measured | 10.60 | Hysterectomy/ovariectomy | WHR | 0.73-0.76 | NA | 0.80 (0.30-2.10) |  |
| 1998 | Kaaks | The Netherlands | Prospective cohort studies | 39-73 | 275 | 11,663 | Technicians measured | 10.60 | Hysterectomy/ovariectomy | WHR | 0.76-0.80 | NA | 1.44 (0.67-3.10) |  |
| 1998 | Kaaks | The Netherlands | Prospective cohort studies | 39-73 | 275 | 11,663 | Technicians measured | 10.60 | Hysterectomy/ovariectomy | WHR | >0.80 | NA | 0.94 (0.41-2.16) |  |
| 1999 | Huang | USA | Prospective cohort studies | 30-55 | 1,037 | 47,382 | Self reported | 7.00 | Premenopause | WC | 38.1-70.866 | NA | 1.00 | Age, height, history of benign breast disease, family history, age at menarche, physical activity, age at first birth and parity, BMI. |
| 1999 | Huang | USA | Prospective cohort studies | 30-55 | 1,037 | 47,382 | Self reported | 7.00 | Premenopause | WC | 71.12-75.946 | NA | 1.07 (0.71-1.61) |  |
| 1999 | Huang | USA | Prospective cohort studies | 30-55 | 1,037 | 47,382 | Self reported | 7.00 | Premenopause | WC | 76.2-81.026 | NA | 1.49 (0.94-2.37) |  |
| 1999 | Huang | USA | Prospective cohort studies | 30-55 | 1,037 | 47,382 | Self reported | 7.00 | Premenopause | WC | 81.28-91.186 | NA | 1.38 (0.79-2.43) |  |
| 1999 | Huang | USA | Prospective cohort studies | 30-55 | 1,037 | 47,382 | Self reported | 7.00 | Premenopause | WC | 91.44-139.7 | NA | 1.74 (0.74-4.07) |  |
| 1999 | Huang | USA | Prospective cohort studies | 30-55 | 1,037 | 47,382 | Self reported | 7.00 | Premenopause | HC | 50.8-93.726 | NA | 1.00 |  |
| 1999 | Huang | USA | Prospective cohort studies | 30-55 | 1,037 | 47,382 | Self reported | 7.00 | Premenopause | HC | 93.98-97.536 | NA | 0.68 (0.45-1.03) |  |
| 1999 | Huang | USA | Prospective cohort studies | 30-55 | 1,037 | 47,382 | Self reported | 7.00 | Premenopause | HC | 97.79-102.616 | NA | 0.76 (0.50-1.18) |  |
| 1999 | Huang | USA | Prospective cohort studies | 30-55 | 1,037 | 47,382 | Self reported | 7.00 | Premenopause | HC | 102.87-108.204 | NA | 0.57 (0.32-1.03) |  |
| 1999 | Huang | USA | Prospective cohort studies | 30-55 | 1,037 | 47,382 | Self reported | 7.00 | Premenopause | HC | 109.22-165.1 | NA | 0.56 (0.26-1.21) |  |
| 1999 | Huang | USA | Prospective cohort studies | 30-55 | 1,037 | 47,382 | Self reported | 7.00 | Premenopause | WHR | <0.73 | NA | 1.00 |  |
| 1999 | Huang | USA | Prospective cohort studies | 30-55 | 1,037 | 47,382 | Self reported | 7.00 | Premenopause | WHR | 0.73-0.75 | NA | 1.02 (0.68-1.52) |  |
| 1999 | Huang | USA | Prospective cohort studies | 30-55 | 1,037 | 47,382 | Self reported | 7.00 | Premenopause | WHR | 0.76-0.79 | NA | 0.94 (0.62-1.42) |  |
| 1999 | Huang | USA | Prospective cohort studies | 30-55 | 1,037 | 47,382 | Self reported | 7.00 | Premenopause | WHR | 0.80-0.83 | NA | 1.32 (0.84-2.07) |  |
| 1999 | Huang | USA | Prospective cohort studies | 30-55 | 1,037 | 47,382 | Self reported | 7.00 | Premenopause | WHR | ≥0.84 | NA | 1.43 (0.86-2.37) |  |
| 1999 | Huang | USA | Prospective cohort studies | 30-55 | 1,037 | 47,382 | Self reported | 7.00 | Postmenopause | WC | 38.1-70.866 | NA | 1.00 |  |
| 1999 | Huang | USA | Prospective cohort studies | 30-55 | 1,037 | 47,382 | Self reported | 7.00 | Postmenopause | WC | 71.12-75.946 | NA | 1.05 (0.82-1.33) |  |
| 1999 | Huang | USA | Prospective cohort studies | 30-55 | 1,037 | 47,382 | Self reported | 7.00 | Postmenopause | WC | 76.2-81.026 | NA | 1.13 (0.88-1.46) |  |
| 1999 | Huang | USA | Prospective cohort studies | 30-55 | 1,037 | 47,382 | Self reported | 7.00 | Postmenopause | WC | 81.28-91.186 | NA | 1.20 (0.92-1.56) |  |
| 1999 | Huang | USA | Prospective cohort studies | 30-55 | 1,037 | 47,382 | Self reported | 7.00 | Postmenopause | WC | 91.44-139.7 | NA | 1.26 (0.88-1.81) |  |
| 1999 | Huang | USA | Prospective cohort studies | 30-55 | 1,037 | 47,382 | Self reported | 7.00 | Postmenopause | HC | 50.8-93.726 | NA | 1.00 |  |
| 1999 | Huang | USA | Prospective cohort studies | 30-55 | 1,037 | 47,382 | Self reported | 7.00 | Postmenopause | HC | 93.98-97.536 | NA | 1.12 (0.89-1.42) |  |
| 1999 | Huang | USA | Prospective cohort studies | 30-55 | 1,037 | 47,382 | Self reported | 7.00 | Postmenopause | HC | 97.79-102.616 | NA | 1.07 (0.84-1.37) |  |
| 1999 | Huang | USA | Prospective cohort studies | 30-55 | 1,037 | 47,382 | Self reported | 7.00 | Postmenopause | HC | 102.87-108.204 | NA | 0.98 (0.75-1.29) |  |
| 1999 | Huang | USA | Prospective cohort studies | 30-55 | 1,037 | 47,382 | Self reported | 7.00 | Postmenopause | HC | 109.22-165.1 | NA | 1.07 (0.76-1.51) |  |
| 1999 | Huang | USA | Prospective cohort studies | 30-55 | 1,037 | 47,382 | Self reported | 7.00 | Postmenopause | WHR | <0.73 | NA | 1.00 |  |
| 1999 | Huang | USA | Prospective cohort studies | 30-55 | 1,037 | 47,382 | Self reported | 7.00 | Postmenopause | WHR | 0.73-0.75 | NA | 1.08 (0.85-1.37) |  |
| 1999 | Huang | USA | Prospective cohort studies | 30-55 | 1,037 | 47,382 | Self reported | 7.00 | Postmenopause | WHR | 0.76-0.79 | NA | 1.02 (0.81-1.28) |  |
| 1999 | Huang | USA | Prospective cohort studies | 30-55 | 1,037 | 47,382 | Self reported | 7.00 | Postmenopause | WHR | 0.80-0.83 | NA | 1.34 (1.06-1.68) |  |
| 1999 | Huang | USA | Prospective cohort studies | 30-55 | 1,037 | 47,382 | Self reported | 7.00 | Postmenopause | WHR | ≥0.84 | NA | 1.22 (0.96-1.55) |  |
| 1999 | Sonnenschein | USA | Prospective cohort studies | 35-65 | 259 | 8,416 | Technicians measured | 6.60 | Premenopause | BMI | <21.5 | NA | 1.00 | Age, BMI, age at menarche, age at first birth, history of breast biopsy, and history of BC in mother and sisters. |
| 1999 | Sonnenschein | USA | Prospective cohort studies | 35-65 | 259 | 8,416 | Technicians measured | 6.60 | Premenopause | BMI | 21.5-23.25 | NA | 0.90 (0.54-1.51) |  |
| 1999 | Sonnenschein | USA | Prospective cohort studies | 35-65 | 259 | 8,416 | Technicians measured | 6.60 | Premenopause | BMI | 23.25-26.36 | NA | 0.77 (0.44-1.33) |  |
| 1999 | Sonnenschein | USA | Prospective cohort studies | 35-65 | 259 | 8,416 | Technicians measured | 6.60 | Premenopause | BMI | >26.36 | NA | 0.81 (0.45-1.45) |  |
| 1999 | Sonnenschein | USA | Prospective cohort studies | 35-65 | 259 | 8,416 | Technicians measured | 6.60 | Premenopause | WHR | <0.698 | NA | 1.00 |  |
| 1999 | Sonnenschein | USA | Prospective cohort studies | 35-65 | 259 | 8,416 | Technicians measured | 6.60 | Premenopause | WHR | 0.698-0.735 | NA | 1.22 (0.66-2.25) |  |
| 1999 | Sonnenschein | USA | Prospective cohort studies | 35-65 | 259 | 8,416 | Technicians measured | 6.60 | Premenopause | WHR | 0.735-0.778 | NA | 1.86 (1.06-3.28) |  |
| 1999 | Sonnenschein | USA | Prospective cohort studies | 35-65 | 259 | 8,416 | Technicians measured | 6.60 | Premenopause | WHR | >0.778 | NA | 1.86 (1.01-3.45) |  |
| 1999 | Sonnenschein | USA | Prospective cohort studies | 35-65 | 259 | 8,416 | Technicians measured | 6.60 | Postmenopause | BMI | <22.32 | NA | 1.00 |  |
| 1999 | Sonnenschein | USA | Prospective cohort studies | 35-65 | 259 | 8,416 | Technicians measured | 6.60 | Postmenopause | BMI | 22.32-24.69 | NA | 1.45 (0.84-2.48) |  |
| 1999 | Sonnenschein | USA | Prospective cohort studies | 35-65 | 259 | 8,416 | Technicians measured | 6.60 | Postmenopause | BMI | 24.69-27.46 | NA | 2.29 (1.37-3.82) |  |
| 1999 | Sonnenschein | USA | Prospective cohort studies | 35-65 | 259 | 8,416 | Technicians measured | 6.60 | Postmenopause | BMI | >27.46 | NA | 2.40 (1.42-4.08) |  |
| 1999 | Sonnenschein | USA | Prospective cohort studies | 35-65 | 259 | 8,416 | Technicians measured | 6.60 | Postmenopause | WHR | <0.73 | NA | 1.00 |  |
| 1999 | Sonnenschein | USA | Prospective cohort studies | 35-65 | 259 | 8,416 | Technicians measured | 6.60 | Postmenopause | WHR | 0.73-0.773 | NA | 1.10 (0.68-1.78) |  |
| 1999 | Sonnenschein | USA | Prospective cohort studies | 35-65 | 259 | 8,416 | Technicians measured | 6.60 | Postmenopause | WHR | 0.773-0.817 | NA | 1.22 (0.76-1.96) |  |
| 1999 | Sonnenschein | USA | Prospective cohort studies | 35-65 | 259 | 8,416 | Technicians measured | 6.60 | Postmenopause | WHR | >0.817 | NA | 0.94 (0.56-1.57) |  |
| 2000 | Muti | Italy | Prospective cohort studies | 35–69 | 144 | 720 | Technicians measured | 5.50 | Premenopause | WHR | ≤0.75 | NA | 1.00 | Age, time at blood drawing, recruitment center, recruitment period, and BMI. |
| 2000 | Muti | Italy | Prospective cohort studies | 35–69 | 144 | 720 | Technicians measured | 5.50 | Premenopause | WHR | 0.75-0.8 | NA | 1.60 (0.80-3.20) |  |
| 2000 | Muti | Italy | Prospective cohort studies | 35–69 | 144 | 720 | Technicians measured | 5.50 | Premenopause | WHR | ≥0.84 | NA | 2.20 (1.00-4.80) |  |
| 2000 | Muti | Italy | Prospective cohort studies | 35–69 | 144 | 720 | Technicians measured | 5.50 | Postmenopause | WHR | ≤0.79 | NA | 1.00 |  |
| 2000 | Muti | Italy | Prospective cohort studies | 35–69 | 144 | 720 | Technicians measured | 5.50 | Postmenopause | WHR | 0.79-0.84 | NA | 0.50 (0.20-1.10) |  |
| 2000 | Muti | Italy | Prospective cohort studies | 35–69 | 144 | 720 | Technicians measured | 5.50 | Postmenopause | WHR | ≥0.84 | NA | 1.10 (0.60-2.20) |  |
| 2002 | Sellers | USA | Prospective cohort studies | 55-69 | 1,874 | 37,105 | Self reported | 9.00 | Postmenopause | BMI | ≤22.89 | ER+ | 1.00 | NA. |
| 2002 | Sellers | USA | Prospective cohort studies | 55-69 | 1,874 | 37,105 | Self reported | 9.00 | Postmenopause | BMI | 22.90–25.04 | ER+ | 1.25 (1.01-1.55) |  |
| 2002 | Sellers | USA | Prospective cohort studies | 55-69 | 1,874 | 37,105 | Self reported | 9.00 | Postmenopause | BMI | 25.05–27.43 | ER+ | 1.35 (1.08-1.68) |  |
| 2002 | Sellers | USA | Prospective cohort studies | 55-69 | 1,874 | 37,105 | Self reported | 9.00 | Postmenopause | BMI | 27.44–30.69 | ER+ | 1.79 (1.43-2.23) |  |
| 2002 | Sellers | USA | Prospective cohort studies | 55-69 | 1,874 | 37,105 | Self reported | 9.00 | Postmenopause | BMI | ≥30.70 | ER+ | 2.00 (1.58-2.53) |  |
| 2002 | Sellers | USA | Prospective cohort studies | 55-69 | 1,874 | 37,105 | Self reported | 9.00 | Postmenopause | WHR | ≤0.76 | ER+ | 1.00 |  |
| 2002 | Sellers | USA | Prospective cohort studies | 55-69 | 1,874 | 37,105 | Self reported | 9.00 | Postmenopause | WHR | 0.77–0.80 | ER+ | 0.98 (0.79-1.21) |  |
| 2002 | Sellers | USA | Prospective cohort studies | 55-69 | 1,874 | 37,105 | Self reported | 9.00 | Postmenopause | WHR | 0.81–0.85 | ER+ | 0.91 (0.74-1.12) |  |
| 2002 | Sellers | USA | Prospective cohort studies | 55-69 | 1,874 | 37,105 | Self reported | 9.00 | Postmenopause | WHR | 0.86–0.90 | ER+ | 0.91 (0.73-1.14) |  |
| 2002 | Sellers | USA | Prospective cohort studies | 55-69 | 1,874 | 37,105 | Self reported | 9.00 | Postmenopause | WHR | >0.90 | ER+ | 1.01 (0.82-1.26) |  |
| 2002 | Sellers | USA | Prospective cohort studies | 55-69 | 1,874 | 37,105 | Self reported | 9.00 | Postmenopause | WC | ≤76 | ER+ | 1.00 |  |
| 2002 | Sellers | USA | Prospective cohort studies | 55-69 | 1,874 | 37,105 | Self reported | 9.00 | Postmenopause | WC | 77–82 | ER+ | 1.15 (0.92-1.44) |  |
| 2002 | Sellers | USA | Prospective cohort studies | 55-69 | 1,874 | 37,105 | Self reported | 9.00 | Postmenopause | WC | 83–90 | ER+ | 1.04 (0.81-1.32) |  |
| 2002 | Sellers | USA | Prospective cohort studies | 55-69 | 1,874 | 37,105 | Self reported | 9.00 | Postmenopause | WC | 91–99 | ER+ | 1.03 (0.78-1.35) |  |
| 2002 | Sellers | USA | Prospective cohort studies | 55-69 | 1,874 | 37,105 | Self reported | 9.00 | Postmenopause | WC | >99 | ER+ | 1.05 (0.77-1.44) |  |
| 2002 | Sellers | USA | Prospective cohort studies | 55-69 | 1,874 | 37,105 | Self reported | 9.00 | Postmenopause | BMI | ≤22.89 | ER- | 1.00 |  |
| 2002 | Sellers | USA | Prospective cohort studies | 55-69 | 1,874 | 37,105 | Self reported | 9.00 | Postmenopause | BMI | 22.90–25.04 | ER- | 1.35 (0.84-2.16) |  |
| 2002 | Sellers | USA | Prospective cohort studies | 55-69 | 1,874 | 37,105 | Self reported | 9.00 | Postmenopause | BMI | 25.05–27.43 | ER- | 1.41 (0.86-2.30) |  |
| 2002 | Sellers | USA | Prospective cohort studies | 55-69 | 1,874 | 37,105 | Self reported | 9.00 | Postmenopause | BMI | 27.44–30.69 | ER- | 1.98 (1.21-3.22) |  |
| 2002 | Sellers | USA | Prospective cohort studies | 55-69 | 1,874 | 37,105 | Self reported | 9.00 | Postmenopause | BMI | ≥30.70 | ER- | 1.38 (0.78-2.43) |  |
| 2002 | Sellers | USA | Prospective cohort studies | 55-69 | 1,874 | 37,105 | Self reported | 9.00 | Postmenopause | WHR | ≤0.76 | ER- | 1.00 |  |
| 2002 | Sellers | USA | Prospective cohort studies | 55-69 | 1,874 | 37,105 | Self reported | 9.00 | Postmenopause | WHR | 0.77–0.80 | ER- | 0.95 (0.61-1.48) |  |
| 2002 | Sellers | USA | Prospective cohort studies | 55-69 | 1,874 | 37,105 | Self reported | 9.00 | Postmenopause | WHR | 0.81–0.85 | ER- | 0.59 (0.36-0.95) |  |
| 2002 | Sellers | USA | Prospective cohort studies | 55-69 | 1,874 | 37,105 | Self reported | 9.00 | Postmenopause | WHR | 0.86–0.90 | ER- | 0.98 (0.62-1.55) |  |
| 2002 | Sellers | USA | Prospective cohort studies | 55-69 | 1,874 | 37,105 | Self reported | 9.00 | Postmenopause | WHR | >0.90 | ER- | 0.81 (0.50-1.31) |  |
| 2002 | Sellers | USA | Prospective cohort studies | 55-69 | 1,874 | 37,105 | Self reported | 9.00 | Postmenopause | WC | ≤76 | ER- | 1.00 |  |
| 2002 | Sellers | USA | Prospective cohort studies | 55-69 | 1,874 | 37,105 | Self reported | 9.00 | Postmenopause | WC | 77–82 | ER- | 1.13 (0.70-1.81) |  |
| 2002 | Sellers | USA | Prospective cohort studies | 55-69 | 1,874 | 37,105 | Self reported | 9.00 | Postmenopause | WC | 83–90 | ER- | 0.90 (0.58-1.68) |  |
| 2002 | Sellers | USA | Prospective cohort studies | 55-69 | 1,874 | 37,105 | Self reported | 9.00 | Postmenopause | WC | 91–99 | ER- | 1.00 (0.54-1.85) |  |
| 2002 | Sellers | USA | Prospective cohort studies | 55-69 | 1,874 | 37,105 | Self reported | 9.00 | Postmenopause | WC | >99 | ER- | 1.03 (0.50-2.09) |  |
| 2002 | Sellers | USA | Prospective cohort studies | 55-69 | 1,874 | 37,105 | Self reported | 9.00 | Postmenopause | BMI | ≤22.89 | PR+ | 1.00 |  |
| 2002 | Sellers | USA | Prospective cohort studies | 55-69 | 1,874 | 37,105 | Self reported | 9.00 | Postmenopause | BMI | 22.90–25.04 | PR+ | 1.30 (1.02-1.65) |  |
| 2002 | Sellers | USA | Prospective cohort studies | 55-69 | 1,874 | 37,105 | Self reported | 9.00 | Postmenopause | BMI | 25.05–27.43 | PR+ | 1.48 (1.15-1.89) |  |
| 2002 | Sellers | USA | Prospective cohort studies | 55-69 | 1,874 | 37,105 | Self reported | 9.00 | Postmenopause | BMI | 27.44–30.69 | PR+ | 1.98 (1.55-2.54) |  |
| 2002 | Sellers | USA | Prospective cohort studies | 55-69 | 1,874 | 37,105 | Self reported | 9.00 | Postmenopause | BMI | ≥30.70 | PR+ | 2.24 (1.72-2.91) |  |
| 2002 | Sellers | USA | Prospective cohort studies | 55-69 | 1,874 | 37,105 | Self reported | 9.00 | Postmenopause | WHR | ≤0.76 | PR+ | 1.00 |  |
| 2002 | Sellers | USA | Prospective cohort studies | 55-69 | 1,874 | 37,105 | Self reported | 9.00 | Postmenopause | WHR | 0.77–0.80 | PR+ | 0.98 (0.77-1.25) |  |
| 2002 | Sellers | USA | Prospective cohort studies | 55-69 | 1,874 | 37,105 | Self reported | 9.00 | Postmenopause | WHR | 0.81–0.85 | PR+ | 0.93 (0.74-1.18) |  |
| 2002 | Sellers | USA | Prospective cohort studies | 55-69 | 1,874 | 37,105 | Self reported | 9.00 | Postmenopause | WHR | 0.86–0.90 | PR+ | 0.88 (0.69-1.13) |  |
| 2002 | Sellers | USA | Prospective cohort studies | 55-69 | 1,874 | 37,105 | Self reported | 9.00 | Postmenopause | WHR | >0.90 | PR+ | 1.05 (0.83-1.34) |  |
| 2002 | Sellers | USA | Prospective cohort studies | 55-69 | 1,874 | 37,105 | Self reported | 9.00 | Postmenopause | WC | ≤76 | PR+ | 1.00 |  |
| 2002 | Sellers | USA | Prospective cohort studies | 55-69 | 1,874 | 37,105 | Self reported | 9.00 | Postmenopause | WC | 77–82 | PR+ | 1.17 (0.91-1.50) |  |
| 2002 | Sellers | USA | Prospective cohort studies | 55-69 | 1,874 | 37,105 | Self reported | 9.00 | Postmenopause | WC | 83–90 | PR+ | 1.05 (0.80-1.38) |  |
| 2002 | Sellers | USA | Prospective cohort studies | 55-69 | 1,874 | 37,105 | Self reported | 9.00 | Postmenopause | WC | 91–99 | PR+ | 1.06 (0.78-1.45) |  |
| 2002 | Sellers | USA | Prospective cohort studies | 55-69 | 1,874 | 37,105 | Self reported | 9.00 | Postmenopause | WC | >99 | PR+ | 1.10 (0.78-1.57) |  |
| 2002 | Sellers | USA | Prospective cohort studies | 55-69 | 1,874 | 37,105 | Self reported | 9.00 | Postmenopause | BMI | ≤22.89 | PR- | 1.00 |  |
| 2002 | Sellers | USA | Prospective cohort studies | 55-69 | 1,874 | 37,105 | Self reported | 9.00 | Postmenopause | BMI | 22.90–25.04 | PR- | 1.11 (0.78-1.59) |  |
| 2002 | Sellers | USA | Prospective cohort studies | 55-69 | 1,874 | 37,105 | Self reported | 9.00 | Postmenopause | BMI | 25.05–27.43 | PR- | 0.99 (0.67-1.45) |  |
| 2002 | Sellers | USA | Prospective cohort studies | 55-69 | 1,874 | 37,105 | Self reported | 9.00 | Postmenopause | BMI | 27.44–30.69 | PR- | 1.45 (1.00-2.10) |  |
| 2002 | Sellers | USA | Prospective cohort studies | 55-69 | 1,874 | 37,105 | Self reported | 9.00 | Postmenopause | BMI | ≥30.70 | PR- | 0.96 (0.62-1.49) |  |
| 2002 | Sellers | USA | Prospective cohort studies | 55-69 | 1,874 | 37,105 | Self reported | 9.00 | Postmenopause | WHR | ≤0.76 | PR- | 1.00 |  |
| 2002 | Sellers | USA | Prospective cohort studies | 55-69 | 1,874 | 37,105 | Self reported | 9.00 | Postmenopause | WHR | 0.77–0.80 | PR- | 0.95 (0.67-1.36) |  |
| 2002 | Sellers | USA | Prospective cohort studies | 55-69 | 1,874 | 37,105 | Self reported | 9.00 | Postmenopause | WHR | 0.81–0.85 | PR- | 0.70 (0.48-1.01) |  |
| 2002 | Sellers | USA | Prospective cohort studies | 55-69 | 1,874 | 37,105 | Self reported | 9.00 | Postmenopause | WHR | 0.86–0.90 | PR- | 1.01 (0.70-1.46) |  |
| 2002 | Sellers | USA | Prospective cohort studies | 55-69 | 1,874 | 37,105 | Self reported | 9.00 | Postmenopause | WHR | >0.90 | PR- | 0.88 (0.60-1.30) |  |
| 2002 | Sellers | USA | Prospective cohort studies | 55-69 | 1,874 | 37,105 | Self reported | 9.00 | Postmenopause | WC | ≤76 | PR- | 1.00 |  |
| 2002 | Sellers | USA | Prospective cohort studies | 55-69 | 1,874 | 37,105 | Self reported | 9.00 | Postmenopause | WC | 77–82 | PR- | 1.13 (0.78-1.63) |  |
| 2002 | Sellers | USA | Prospective cohort studies | 55-69 | 1,874 | 37,105 | Self reported | 9.00 | Postmenopause | WC | 83–90 | PR- | 1.01 (0.67-1.52) |  |
| 2002 | Sellers | USA | Prospective cohort studies | 55-69 | 1,874 | 37,105 | Self reported | 9.00 | Postmenopause | WC | 91–99 | PR- | 0.95 (0.58-1.54) |  |
| 2002 | Sellers | USA | Prospective cohort studies | 55-69 | 1,874 | 37,105 | Self reported | 9.00 | Postmenopause | WC | >99 | PR- | 0.98 (0.56-1.72) |  |
| 2004 | Lahmann | 9 European countries | Prospective cohort studies | 18-80 | 1,879 | 176,886 | Technicians measured | 4.70 | Premenopause | BMI | <21.6 | NA | 1.00 | Age, study center, educational attainment, smoking status, alcohol consumption, age at first pregnancy and parity, age at menarche, HRT, and BMI. |
| 2004 | Lahmann | 9 European countries | Prospective cohort studies | 18-80 | 1,879 | 176,886 | Technicians measured | 4.70 | Premenopause | BMI | 21.6-23.5 | NA | 0.95 (0.73-1.23) |  |
| 2004 | Lahmann | 9 European countries | Prospective cohort studies | 18-80 | 1,879 | 176,886 | Technicians measured | 4.70 | Premenopause | BMI | 23.6-25.6 | NA | 0.78 (0.59-1.04) |  |
| 2004 | Lahmann | 9 European countries | Prospective cohort studies | 18-80 | 1,879 | 176,886 | Technicians measured | 4.70 | Premenopause | BMI | 25.7-28.7 | NA | 0.80 (0.59-1.09) |  |
| 2004 | Lahmann | 9 European countries | Prospective cohort studies | 18-80 | 1,879 | 176,886 | Technicians measured | 4.70 | Premenopause | BMI | ≥28.8 | NA | 0.82 (0.59-1.14) |  |
| 2004 | Lahmann | 9 European countries | Prospective cohort studies | 18-80 | 1,879 | 176,886 | Technicians measured | 4.70 | Premenopause | WC | <71 | NA | 1.00 |  |
| 2004 | Lahmann | 9 European countries | Prospective cohort studies | 18-80 | 1,879 | 176,886 | Technicians measured | 4.70 | Premenopause | WC | 71-75.9 | NA | 1.43 (1.11-1.84) |  |
| 2004 | Lahmann | 9 European countries | Prospective cohort studies | 18-80 | 1,879 | 176,886 | Technicians measured | 4.70 | Premenopause | WC | 76-81.4 | NA | 0.93 (0.67-1.31) |  |
| 2004 | Lahmann | 9 European countries | Prospective cohort studies | 18-80 | 1,879 | 176,886 | Technicians measured | 4.70 | Premenopause | WC | 81.5-89.2 | NA | 1.34 (0.92-1.94) |  |
| 2004 | Lahmann | 9 European countries | Prospective cohort studies | 18-80 | 1,879 | 176,886 | Technicians measured | 4.70 | Premenopause | WC | ≥89.3 | NA | 1.81 (1.11-2.97) |  |
| 2004 | Lahmann | 9 European countries | Prospective cohort studies | 18-80 | 1,879 | 176,886 | Technicians measured | 4.70 | Premenopause | HC | <94 | NA | 1.00 |  |
| 2004 | Lahmann | 9 European countries | Prospective cohort studies | 18-80 | 1,879 | 176,886 | Technicians measured | 4.70 | Premenopause | HC | 94-97.9 | NA | 1.08 (0.82-1.43) |  |
| 2004 | Lahmann | 9 European countries | Prospective cohort studies | 18-80 | 1,879 | 176,886 | Technicians measured | 4.70 | Premenopause | HC | 98-102.4 | NA | 1.09 (0.80-1.48) |  |
| 2004 | Lahmann | 9 European countries | Prospective cohort studies | 18-80 | 1,879 | 176,886 | Technicians measured | 4.70 | Premenopause | HC | 102.5-107.9 | NA | 1.44 (1.02-2.02) |  |
| 2004 | Lahmann | 9 European countries | Prospective cohort studies | 18-80 | 1,879 | 176,886 | Technicians measured | 4.70 | Premenopause | HC | ≥108 | NA | 1.70 (1.05-2.77) |  |
| 2004 | Lahmann | 9 European countries | Prospective cohort studies | 18-80 | 1,879 | 176,886 | Technicians measured | 4.70 | Premenopause | WHR | <0.736 | NA | 1.00 |  |
| 2004 | Lahmann | 9 European countries | Prospective cohort studies | 18-80 | 1,879 | 176,886 | Technicians measured | 4.70 | Premenopause | WHR | 0.737-0.770 | NA | 0.88 (0.68-1.14) |  |
| 2004 | Lahmann | 9 European countries | Prospective cohort studies | 18-80 | 1,879 | 176,886 | Technicians measured | 4.70 | Premenopause | WHR | 0.771-0.803 | NA | 0.87 (0.66-1.16) |  |
| 2004 | Lahmann | 9 European countries | Prospective cohort studies | 18-80 | 1,879 | 176,886 | Technicians measured | 4.70 | Premenopause | WHR | 0.804-0.846 | NA | 1.06 (0.79-1.43) |  |
| 2004 | Lahmann | 9 European countries | Prospective cohort studies | 18-80 | 1,879 | 176,886 | Technicians measured | 4.70 | Premenopause | WHR | ≥0.847 | NA | 1.05 (0.74-1.50) |  |
| 2004 | Macinnis | Australia | Prospective cohort studies | 27-75 | 357 | 13,598 | Technicians measured | 9.10 | Postmenopause | BMI (Per5 kg/m2） | Q1 | NA | 1.00 | Age at attendance, country of birth, highest level of education, physical activity, and HRT us. |
| 2004 | Macinnis | Australia | Prospective cohort studies | 27-75 | 357 | 13,598 | Technicians measured | 9.10 | Postmenopause | BMI (Per5 kg/m2） | Q2 | NA | 1.20 (0.90-1.50) |  |
| 2004 | Macinnis | Australia | Prospective cohort studies | 27-75 | 357 | 13,598 | Technicians measured | 9.10 | Postmenopause | BMI (Per5 kg/m2） | Q3 | NA | 1.40 (1.00-1.90) |  |
| 2004 | Macinnis | Australia | Prospective cohort studies | 27-75 | 357 | 13,598 | Technicians measured | 9.10 | Postmenopause | BMI (Per5 kg/m2） | Q4 | NA | NA |  |
| 2004 | Macinnis | Australia | Prospective cohort studies | 27-75 | 357 | 13,598 | Technicians measured | 9.10 | Postmenopause | WC | Q1 | NA | 1.00 |  |
| 2004 | Macinnis | Australia | Prospective cohort studies | 27-75 | 357 | 13,598 | Technicians measured | 9.10 | Postmenopause | WC | Q2 | NA | 1.30 (0.90-1.80) |  |
| 2004 | Macinnis | Australia | Prospective cohort studies | 27-75 | 357 | 13,598 | Technicians measured | 9.10 | Postmenopause | WC | Q3 | NA | 1.20 (0.80-1.70) |  |
| 2004 | Macinnis | Australia | Prospective cohort studies | 27-75 | 357 | 13,598 | Technicians measured | 9.10 | Postmenopause | WC | Q4 | NA | 1.50 (1.10-2.10) |  |
| 2004 | Macinnis | Australia | Prospective cohort studies | 27-75 | 357 | 13,598 | Technicians measured | 9.10 | Postmenopause | HC | Q1 | NA | 1.00 |  |
| 2004 | Macinnis | Australia | Prospective cohort studies | 27-75 | 357 | 13,598 | Technicians measured | 9.10 | Postmenopause | HC | Q2 | NA | 1.30 (0.90-1.80) |  |
| 2004 | Macinnis | Australia | Prospective cohort studies | 27-75 | 357 | 13,598 | Technicians measured | 9.10 | Postmenopause | HC | Q3 | NA | 1.20 (0.90-1.60) |  |
| 2004 | Macinnis | Australia | Prospective cohort studies | 27-75 | 357 | 13,598 | Technicians measured | 9.10 | Postmenopause | HC | Q4 | NA | 1.50 (1.10-2.10) |  |
| 2004 | Macinnis | Australia | Prospective cohort studies | 27-75 | 357 | 13,598 | Technicians measured | 9.10 | Postmenopause | WHR | Q1 | NA | 1.00 |  |
| 2004 | Macinnis | Australia | Prospective cohort studies | 27-75 | 357 | 13,598 | Technicians measured | 9.10 | Postmenopause | WHR | Q2 | NA | 1.00 (0.70-1.30) |  |
| 2004 | Macinnis | Australia | Prospective cohort studies | 27-75 | 357 | 13,598 | Technicians measured | 9.10 | Postmenopause | WHR | Q3 | NA | 1.10 (0.80-1.50) |  |
| 2004 | Macinnis | Australia | Prospective cohort studies | 27-75 | 357 | 13,598 | Technicians measured | 9.10 | Postmenopause | WHR | Q4 | NA | 1.20 (0.80-1.60) |  |
| 2004 | Macinnis | Australia | Prospective cohort studies | 27-75 | 357 | 13,598 | Technicians measured | 9.10 | Postmenopause | BMI (Per5 kg/m2） | NA | ER+ | 1.25 (1.05-1.49) | Age, country of birth, exercise, HRT use, and highest level of education. |
| 2004 | Macinnis | Australia | Prospective cohort studies | 27-75 | 357 | 13,598 | Technicians measured | 9.10 | Postmenopause | WC | NA | ER+ | 1.24 (1.06-1.46) |  |
| 2004 | Macinnis | Australia | Prospective cohort studies | 27-75 | 357 | 13,598 | Technicians measured | 9.10 | Postmenopause | HC | NA | ER+ | 1.29 (1.08-1.54) |  |
| 2004 | Macinnis | Australia | Prospective cohort studies | 27-75 | 357 | 13,598 | Technicians measured | 9.10 | Postmenopause | WHR | NA | ER+ | 1.19 (0.88-1.61) |  |
| 2004 | Macinnis | Australia | Prospective cohort studies | 27-75 | 357 | 13,598 | Technicians measured | 9.10 | Postmenopause | BMI (Per5 kg/m2） | NA | ER- | 0.82 (0.55-1.24) |  |
| 2004 | Macinnis | Australia | Prospective cohort studies | 27-75 | 357 | 13,598 | Technicians measured | 9.10 | Postmenopause | WC | NA | ER- | 0.79 (0.58-1.07) |  |
| 2004 | Macinnis | Australia | Prospective cohort studies | 27-75 | 357 | 13,598 | Technicians measured | 9.10 | Postmenopause | HC | NA | ER- | 0.84 (0。6-1.16) |  |
| 2004 | Macinnis | Australia | Prospective cohort studies | 27-75 | 357 | 13,598 | Technicians measured | 9.10 | Postmenopause | HC | NA | ER- | 0.69 (0.41-1.16) |  |
| 2004 | Macinnis | Australia | Prospective cohort studies | 27-75 | 357 | 13,598 | Technicians measured | 9.10 | Postmenopause | BMI (Per5 kg/m2） | NA | PR+ | 1.17 (0.95-1.44) |  |
| 2004 | Macinnis | Australia | Prospective cohort studies | 27-75 | 357 | 13,598 | Technicians measured | 9.10 | Postmenopause | WC | NA | PR+ | 1.18 (0.99-1.40) |  |
| 2004 | Macinnis | Australia | Prospective cohort studies | 27-75 | 357 | 13,598 | Technicians measured | 9.10 | Postmenopause | HC | NA | PR+ | 1.21 (1.00-1.48) |  |
| 2004 | Macinnis | Australia | Prospective cohort studies | 27-75 | 357 | 13,598 | Technicians measured | 9.10 | Postmenopause | WHR | NA | PR+ | 1.13 (0.82-1.57) |  |
| 2004 | Macinnis | Australia | Prospective cohort studies | 27-75 | 357 | 13,598 | Technicians measured | 9.10 | Postmenopause | BMI (Per5 kg/m2） | NA | PR- | 1.11 (0.86-1.45) |  |
| 2004 | Macinnis | Australia | Prospective cohort studies | 27-75 | 357 | 13,598 | Technicians measured | 9.10 | Postmenopause | WC | NA | PR- | 1.05 (0.82-1.33) |  |
| 2004 | Macinnis | Australia | Prospective cohort studies | 27-75 | 357 | 13,598 | Technicians measured | 9.10 | Postmenopause | HC | NA | PR- | 1.12 (0.86-1.47) |  |
| 2004 | Macinnis | Australia | Prospective cohort studies | 27-75 | 357 | 13,598 | Technicians measured | 9.10 | Postmenopause | WHR | NA | PR- | 0.93 (0.58-1.49) |  |
| 2006 | Krebs | USA | Prospective cohort studies | ≥65 | 456 | 7,523 | Technicians measured | 11.30 | Postmenopause | BMI | <23 | NA | 1.00 | Age, current and past estrogen use, hip bone mineral density, benign breast disease, family history of BC, walking for exercise, education, live births, age at menarche, and current and past smoking status. |
| 2006 | Krebs | USA | Prospective cohort studies | ≥65 | 456 | 7,523 | Technicians measured | 11.30 | Postmenopause | BMI | 23-25.6 | NA | 0.82 (0.58-1.15) |  |
| 2006 | Krebs | USA | Prospective cohort studies | ≥65 | 456 | 7,523 | Technicians measured | 11.30 | Postmenopause | BMI | 25.7-28.9 | NA | 1.01 (0.72-1.41) |  |
| 2006 | Krebs | USA | Prospective cohort studies | ≥65 | 456 | 7,523 | Technicians measured | 11.30 | Postmenopause | BMI | >28.9 | NA | 1.29 (0.92-1.81) |  |
| 2006 | Krebs | USA | Prospective cohort studies | ≥65 | 456 | 7,523 | Technicians measured | 11.30 | Postmenopause | WC | <75.7 | NA | 1.00 |  |
| 2006 | Krebs | USA | Prospective cohort studies | ≥65 | 456 | 7,523 | Technicians measured | 11.30 | Postmenopause | WC | 75.8-82.9 | NA | 0.95 (0.67-1.36) |  |
| 2006 | Krebs | USA | Prospective cohort studies | ≥65 | 456 | 7,523 | Technicians measured | 11.30 | Postmenopause | WC | 83-91.2 | NA | 1.24 (0.88-1.73) |  |
| 2006 | Krebs | USA | Prospective cohort studies | ≥65 | 456 | 7,523 | Technicians measured | 11.30 | Postmenopause | WC | >91.2 | NA | 1.40 (0.98-1.98) |  |
| 2006 | Krebs | USA | Prospective cohort studies | ≥65 | 456 | 7,523 | Technicians measured | 11.30 | Postmenopause | WHR | <0.78 | NA | 1.00 |  |
| 2006 | Krebs | USA | Prospective cohort studies | ≥65 | 456 | 7,523 | Technicians measured | 11.30 | Postmenopause | WHR | 0.78-0.83 | NA | 1.25 (0.90-1.75) |  |
| 2006 | Krebs | USA | Prospective cohort studies | ≥65 | 456 | 7,523 | Technicians measured | 11.30 | Postmenopause | WHR | 0.84-0.88 | NA | 1.12 (0.79-1.58) |  |
| 2006 | Krebs | USA | Prospective cohort studies | ≥65 | 456 | 7,523 | Technicians measured | 11.30 | Postmenopause | WHR | >0.88 | NA | 1.37 (0.98-1.92) |  |
| 2006 | Tehard | France | Prospective cohort studies | 45-70 | 1,135 | 69,116 | Self reported | 3.6 | Premenopausal | BMI | < 18.5 | NA | 1.15 (0.47-2.79) | Age at first birth, first-degree relatives, age at menarche, parity, history of benign breast disease, alcohol consumption, number of years of education, marital status married and physical activity, and BMI. |
| 2006 | Tehard | France | Prospective cohort studies | 45-70 | 1,135 | 69,116 | Self reported | 3.6 | Premenopausal | BMI | 18.5-25 | NA | 1.00 |  |
| 2006 | Tehard | France | Prospective cohort studies | 45-70 | 1,135 | 69,116 | Self reported | 3.6 | Premenopausal | BMI | 25-30 | NA | 0.84 (0.56-1.27) |  |
| 2006 | Tehard | France | Prospective cohort studies | 45-70 | 1,135 | 69,116 | Self reported | 3.6 | Premenopausal | BMI | ≥30 | NA | 0.26 (0.06-1.00) |  |
| 2006 | Tehard | France | Prospective cohort studies | 45-70 | 1,135 | 69,116 | Self reported | 3.6 | Premenopausal | WC | <69 | NA | 1.00 |  |
| 2006 | Tehard | France | Prospective cohort studies | 45-70 | 1,135 | 69,116 | Self reported | 3.6 | Premenopausal | WC | 69-74 | NA | 0.83 (0.58-1.21) |  |
| 2006 | Tehard | France | Prospective cohort studies | 45-70 | 1,135 | 69,116 | Self reported | 3.6 | Premenopausal | WC | 74-79 | NA | 0.98 (0.65-1.48) |  |
| 2006 | Tehard | France | Prospective cohort studies | 45-70 | 1,135 | 69,116 | Self reported | 3.6 | Premenopausal | WC | ≥79 | NA | 0.66 (0.38-1.15) |  |
| 2006 | Tehard | France | Prospective cohort studies | 45-70 | 1,135 | 69,116 | Self reported | 3.6 | Premenopausal | HC | <90 | NA | 1.00 |  |
| 2006 | Tehard | France | Prospective cohort studies | 45-70 | 1,135 | 69,116 | Self reported | 3.6 | Premenopausal | HC | 90-95 | NA | 1.16 (0.78-1.72) |  |
| 2006 | Tehard | France | Prospective cohort studies | 45-70 | 1,135 | 69,116 | Self reported | 3.6 | Premenopausal | HC | 95-100 | NA | 1.29 (0.84-1.98) |  |
| 2006 | Tehard | France | Prospective cohort studies | 45-70 | 1,135 | 69,116 | Self reported | 3.6 | Premenopausal | HC | ≥100 | NA | 1.35 (0.79-2.32) |  |
| 2006 | Tehard | France | Prospective cohort studies | 45-70 | 1,135 | 69,116 | Self reported | 3.6 | Premenopausal | WHR | <0.74 | NA | 1.00 |  |
| 2006 | Tehard | France | Prospective cohort studies | 45-70 | 1,135 | 69,116 | Self reported | 3.6 | Premenopausal | WHR | 0.74-0.78 | NA | 1.05 (0.73-1.49) |  |
| 2006 | Tehard | France | Prospective cohort studies | 45-70 | 1,135 | 69,116 | Self reported | 3.6 | Premenopausal | WHR | 0.78-0.82 | NA | 0.87 (0.59-1.27) |  |
| 2006 | Tehard | France | Prospective cohort studies | 45-70 | 1,135 | 69,116 | Self reported | 3.6 | Premenopausal | WHR | >0.82 | NA | 0.68 (0.43-1.06) |  |
| 2006 | Tehard | France | Prospective cohort studies | 45-70 | 1,135 | 69,116 | Self reported | 3.6 | Postmenopause | BMI | < 18.5 | NA | 0.49 (0.22-1.10) |  |
| 2006 | Tehard | France | Prospective cohort studies | 45-70 | 1,135 | 69,116 | Self reported | 3.6 | Postmenopause | BMI | 18.5-25 | NA | 1.00 |  |
| 2006 | Tehard | France | Prospective cohort studies | 45-70 | 1,135 | 69,116 | Self reported | 3.6 | Postmenopause | BMI | 25-30 | NA | 1.07 (0.89-1.30) |  |
| 2006 | Tehard | France | Prospective cohort studies | 45-70 | 1,135 | 69,116 | Self reported | 3.6 | Postmenopause | BMI | ≥30 | NA | 1.44 (1.04-1.99) |  |
| 2006 | Tehard | France | Prospective cohort studies | 45-70 | 1,135 | 69,116 | Self reported | 3.6 | Postmenopause | WC | <69 | NA | 1.00 |  |
| 2006 | Tehard | France | Prospective cohort studies | 45-70 | 1,135 | 69,116 | Self reported | 3.6 | Postmenopause | WC | 69-74 | NA | 1.17 (0.91-1.49) |  |
| 2006 | Tehard | France | Prospective cohort studies | 45-70 | 1,135 | 69,116 | Self reported | 3.6 | Postmenopause | WC | 74-79 | NA | 1.21 (0.94-1.56) |  |
| 2006 | Tehard | France | Prospective cohort studies | 45-70 | 1,135 | 69,116 | Self reported | 3.6 | Postmenopause | WC | ≥79 | NA | 1.01 (0.73-1.39) |  |
| 2006 | Tehard | France | Prospective cohort studies | 45-70 | 1,135 | 69,116 | Self reported | 3.6 | Postmenopause | HC | <90 | NA | 1.00 |  |
| 2006 | Tehard | France | Prospective cohort studies | 45-70 | 1,135 | 69,116 | Self reported | 3.6 | Postmenopause | HC | 90-95 | NA | 1.12 (0.88-1.43) |  |
| 2006 | Tehard | France | Prospective cohort studies | 45-70 | 1,135 | 69,116 | Self reported | 3.6 | Postmenopause | HC | 95-100 | NA | 1.07 (0.84-1.37) |  |
| 2006 | Tehard | France | Prospective cohort studies | 45-70 | 1,135 | 69,116 | Self reported | 3.6 | Postmenopause | HC | ≥100 | NA | 1.01 (0.75-1.37) |  |
| 2006 | Tehard | France | Prospective cohort studies | 45-70 | 1,135 | 69,116 | Self reported | 3.6 | Postmenopause | WHR | <0.74 | NA | 1.00 |  |
| 2006 | Tehard | France | Prospective cohort studies | 45-70 | 1,135 | 69,116 | Self reported | 3.6 | Postmenopause | WHR | 0.74-0.78 | NA | 1.10 (0.88-1.39) |  |
| 2006 | Tehard | France | Prospective cohort studies | 45-70 | 1,135 | 69,116 | Self reported | 3.6 | Postmenopause | WHR | 0.78-0.82 | NA | 1.08 (0.87-1.35) |  |
| 2006 | Tehard | France | Prospective cohort studies | 45-70 | 1,135 | 69,116 | Self reported | 3.6 | Postmenopause | WHR | >-0.82 | NA | 0.95 (0.75-1.21) |  |
| 2006 | Wu | China | Prospective cohort studies | NR | 104 | 11,889 | Technicians measured | 10.3 | NA | BMI | < 21.6 | NA | 1.00 | Age at enrollment and WHR for analyses of height, weight, age at enrollment and BMI. |
| 2006 | Wu | China | Prospective cohort studies | NR | 104 | 11,889 | Technicians measured | 10.3 | NA | BMI | 21.6–23.6 | NA | 1.20 (0.70-2.20) |  |
| 2006 | Wu | China | Prospective cohort studies | NR | 104 | 11,889 | Technicians measured | 10.3 | NA | BMI | 23.7–26.2 | NA | 1.20 (0.60-2.10) |  |
| 2006 | Wu | China | Prospective cohort studies | NR | 104 | 11,889 | Technicians measured | 10.3 | NA | BMI | >26.2 | NA | 1.90 (1.00-3.40) |  |
| 2006 | Wu | China | Prospective cohort studies | NR | 104 | 11,889 | Technicians measured | 10.3 | NA | WC | <71 | NA | 1.00 |  |
| 2006 | Wu | China | Prospective cohort studies | NR | 104 | 11,889 | Technicians measured | 10.3 | NA | WC | 71–75 | NA | 1.10 (0.60-2.10) |  |
| 2006 | Wu | China | Prospective cohort studies | NR | 104 | 11,889 | Technicians measured | 10.3 | NA | WC | 76–83 | NA | 1.00 (0.50-2.00) |  |
| 2006 | Wu | China | Prospective cohort studies | NR | 104 | 11,889 | Technicians measured | 10.3 | NA | WC | >83 | NA | 1.20 (0.50-2.70) |  |
| 2006 | Wu | China | Prospective cohort studies | NR | 104 | 11,889 | Technicians measured | 10.3 | NA | HC | <90 | NA | 1.00 |  |
| 2006 | Wu | China | Prospective cohort studies | NR | 104 | 11,889 | Technicians measured | 10.3 | NA | HC | 90-94 | NA | 2.40 (1.20-4.80) |  |
| 2006 | Wu | China | Prospective cohort studies | NR | 104 | 11,889 | Technicians measured | 10.3 | NA | HC | 95-100 | NA | 2.00 (1.10-4.20) |  |
| 2006 | Wu | China | Prospective cohort studies | NR | 104 | 11,889 | Technicians measured | 10.3 | NA | HC | >100 | NA | 2.90 (1.10-6.70) |  |
| 2006 | Wu | China | Prospective cohort studies | NR | 104 | 11,889 | Technicians measured | 10.3 | NA | WHR | <0.77 | NA | 1.00 |  |
| 2006 | Wu | China | Prospective cohort studies | NR | 104 | 11,889 | Technicians measured | 10.3 | NA | WHR | 0.77-0.79 | NA | 0.70 (0.40-1.30) |  |
| 2006 | Wu | China | Prospective cohort studies | NR | 104 | 11,889 | Technicians measured | 10.3 | NA | WHR | 0.80-0.85 | NA | 0.90 (0.50-1.50) |  |
| 2006 | Wu | China | Prospective cohort studies | NR | 104 | 11,889 | Technicians measured | 10.3 | NA | WHR | >0.85 | NA | 0.60 (0.30-1.20) |  |
| 2007 | Palmer | African American | Prospective cohort studies | 21-69 | 1,062 | 59,000 | Self reported | 10.00 | Premenopause | BMI | <25 | NA | 1.00 | Age, age at menarche, BMI at age 18 years, parity, age at first birth, vigorous activity, education, and family history of BC. |
| 2007 | Palmer | African American | Prospective cohort studies | 21-69 | 1,062 | 59,000 | Self reported | 10.00 | Premenopause | BMI | 25-29 | NA | 0.92 (0.74-1.15) |  |
| 2007 | Palmer | African American | Prospective cohort studies | 21-69 | 1,062 | 59,000 | Self reported | 10.00 | Premenopause | BMI | 30-34 | NA | 0.97 (0.74-1.28) |  |
| 2007 | Palmer | African American | Prospective cohort studies | 21-69 | 1,062 | 59,000 | Self reported | 10.00 | Premenopause | BMI | ≥35 | NA | 0.87 (0.62-1.21) |  |
| 2007 | Palmer | African American | Prospective cohort studies | 21-69 | 1,062 | 59,000 | Self reported | 10.00 | Premenopause | WC | <71 | NA | 1.00 |  |
| 2007 | Palmer | African American | Prospective cohort studies | 21-69 | 1,062 | 59,000 | Self reported | 10.00 | Premenopause | WC | 71-75.9 | NA | 1.13 (0.85-1.50) |  |
| 2007 | Palmer | African American | Prospective cohort studies | 21-69 | 1,062 | 59,000 | Self reported | 10.00 | Premenopause | WC | 76-81.4 | NA | 1.34 (0.98-1.85) |  |
| 2007 | Palmer | African American | Prospective cohort studies | 21-69 | 1,062 | 59,000 | Self reported | 10.00 | Premenopause | WC | 81.5-89.2 | NA | 1.20 (0.89-1.62) |  |
| 2007 | Palmer | African American | Prospective cohort studies | 21-69 | 1,062 | 59,000 | Self reported | 10.00 | Premenopause | WC | ≥89.3 | NA | 1.04 (0.73-1.48) |  |
| 2007 | Palmer | African American | Prospective cohort studies | 21-69 | 1,062 | 59,000 | Self reported | 10.00 | Premenopause | WHR | <0.71 | NA | 1.00 |  |
| 2007 | Palmer | African American | Prospective cohort studies | 21-69 | 1,062 | 59,000 | Self reported | 10.00 | Premenopause | WHR | 0.71-0.75 | NA | 1.17 (0.87-1.58) |  |
| 2007 | Palmer | African American | Prospective cohort studies | 21-69 | 1,062 | 59,000 | Self reported | 10.00 | Premenopause | WHR | 0.76-0.8 | NA | 1.15 (0.85-1.56) |  |
| 2007 | Palmer | African American | Prospective cohort studies | 21-69 | 1,062 | 59,000 | Self reported | 10.00 | Premenopause | WHR | 0.81-0.86 | NA | 1.27 (0.94-1.72) |  |
| 2007 | Palmer | African American | Prospective cohort studies | 21-69 | 1,062 | 59,000 | Self reported | 10.00 | Premenopause | WHR | ≥0.87 | NA | 1.19 (0.87-1.64) |  |
| 2007 | Palmer | African American | Prospective cohort studies | 21-69 | 1,062 | 59,000 | Self reported | 10.00 | Postmenopause | BMI | <25 | NA | 1.00 |  |
| 2007 | Palmer | African American | Prospective cohort studies | 21-69 | 1,062 | 59,000 | Self reported | 10.00 | Postmenopause | BMI | 25-29 | NA | 0.75 (0.58-0.97) |  |
| 2007 | Palmer | African American | Prospective cohort studies | 21-69 | 1,062 | 59,000 | Self reported | 10.00 | Postmenopause | BMI | 30-34 | NA | 0.92 (0.69-1.21) |  |
| 2007 | Palmer | African American | Prospective cohort studies | 21-69 | 1,062 | 59,000 | Self reported | 10.00 | Postmenopause | BMI | ≥35 | NA | 0.99 (0.72-1.36) |  |
| 2007 | Palmer | African American | Prospective cohort studies | 21-69 | 1,062 | 59,000 | Self reported | 10.00 | Postmenopause | WC | <71 | NA | 1.00 |  |
| 2007 | Palmer | African American | Prospective cohort studies | 21-69 | 1,062 | 59,000 | Self reported | 10.00 | Postmenopause | WC | 71-75.9 | NA | 1.00 (0.70-1.42) |  |
| 2007 | Palmer | African American | Prospective cohort studies | 21-69 | 1,062 | 59,000 | Self reported | 10.00 | Postmenopause | WC | 76-81.4 | NA | 0.88 (0.60-1.29) |  |
| 2007 | Palmer | African American | Prospective cohort studies | 21-69 | 1,062 | 59,000 | Self reported | 10.00 | Postmenopause | WC | 81.5-89.2 | NA | 1.00 (0.71-1.42) |  |
| 2007 | Palmer | African American | Prospective cohort studies | 21-69 | 1,062 | 59,000 | Self reported | 10.00 | Postmenopause | WC | ≥89.3 | NA | 1.05 (0.73-1.51) |  |
| 2007 | Palmer | African American | Prospective cohort studies | 21-69 | 1,062 | 59,000 | Self reported | 10.00 | Postmenopause | WHR | <0.71 | NA | 1.00 |  |
| 2007 | Palmer | African American | Prospective cohort studies | 21-69 | 1,062 | 59,000 | Self reported | 10.00 | Postmenopause | WHR | 0.71-0.75 | NA | 0.73 (0.52-1.03) |  |
| 2007 | Palmer | African American | Prospective cohort studies | 21-69 | 1,062 | 59,000 | Self reported | 10.00 | Postmenopause | WHR | 0.76-0.8 | NA | 0.98 (0.72-1.34) |  |
| 2007 | Palmer | African American | Prospective cohort studies | 21-69 | 1,062 | 59,000 | Self reported | 10.00 | Postmenopause | WHR | 0.81-0.86 | NA | 1.01 (0.74-1.38) |  |
| 2007 | Palmer | African American | Prospective cohort studies | 21-69 | 1,062 | 59,000 | Self reported | 10.00 | Postmenopause | WHR | ≥0.87 | NA | 0.99 (0.72-1.37) |  |
| 2010 | Harris | USA | Prospective cohort studies | NR | 620 | 45,799 | Self reported | 11.00 | Postmenopause | WC | <68.58 | NA | 1.00 | BMI, height, history of benign breast disease, family history of BC, age at menarche, age at first birth, parity, oral contraceptive use, alcohol consumption, and physical activity. |
| 2010 | Harris | USA | Prospective cohort studies | NR | 620 | 45,799 | Self reported | 11.00 | Postmenopause | WC | 68.58-73.03 | NA | 1.06 (0.82-1.38) |  |
| 2010 | Harris | USA | Prospective cohort studies | NR | 620 | 45,799 | Self reported | 11.00 | Postmenopause | WC | 73.66-78.11 | NA | 1.19 (0.90-1.56) |  |
| 2010 | Harris | USA | Prospective cohort studies | NR | 620 | 45,799 | Self reported | 11.00 | Postmenopause | WC | 78.74-86.36 | NA | 1.36 (1.02-1.82) |  |
| 2010 | Harris | USA | Prospective cohort studies | NR | 620 | 45,799 | Self reported | 11.00 | Postmenopause | WC | ≥86.995 | NA | 1.27 (0.88-1.84) |  |
| 2010 | Harris | USA | Prospective cohort studies | NR | 620 | 45,799 | Self reported | 11.00 | Postmenopause | HC | <92.075 | NA | 1.00 |  |
| 2010 | Harris | USA | Prospective cohort studies | NR | 620 | 45,799 | Self reported | 11.00 | Postmenopause | HC | 92.075-95.89 | NA | 1.16 (0.90-1.51) |  |
| 2010 | Harris | USA | Prospective cohort studies | NR | 620 | 45,799 | Self reported | 11.00 | Postmenopause | HC | 96.52-99.70 | NA | 1.13 (0.87-1.47) |  |
| 2010 | Harris | USA | Prospective cohort studies | NR | 620 | 45,799 | Self reported | 11.00 | Postmenopause | HC | 100.33-106.68 | NA | 1.25 (0.93-1.66) |  |
| 2010 | Harris | USA | Prospective cohort studies | NR | 620 | 45,799 | Self reported | 11.00 | Postmenopause | HC | ≥107.32 | NA | 1.17 (0.78-1.74) |  |
| 2010 | Harris | USA | Prospective cohort studies | NR | 620 | 45,799 | Self reported | 11.00 | Postmenopause | WHR | <0.73 | NA | 1.00 |  |
| 2010 | Harris | USA | Prospective cohort studies | NR | 620 | 45,799 | Self reported | 11.00 | Postmenopause | WHR | 0.73-0.75 | NA | 0.85 (0.66-1.10) |  |
| 2010 | Harris | USA | Prospective cohort studies | NR | 620 | 45,799 | Self reported | 11.00 | Postmenopause | WHR | 0.76-0.78 | NA | 1.01 (0.79-1.30) |  |
| 2010 | Harris | USA | Prospective cohort studies | NR | 620 | 45,799 | Self reported | 11.00 | Postmenopause | WHR | 0.79-0.83 | NA | 1.07 (0.83-1.36) |  |
| 2010 | Harris | USA | Prospective cohort studies | NR | 620 | 45,799 | Self reported | 11.00 | Postmenopause | WHR | ≥0.84 | NA | 1.14 (0.88-1.48) |  |
| 2010 | Harris | USA | Prospective cohort studies | NR | 620 | 45,799 | Self reported | 11.00 | Postmenopause | BMI | <20.5 | NA | 1.00 |  |
| 2010 | Harris | USA | Prospective cohort studies | NR | 620 | 45,799 | Self reported | 11.00 | Postmenopause | BMI | 20.6-22 | NA | 0.99 (0.77-1.27) |  |
| 2010 | Harris | USA | Prospective cohort studies | NR | 620 | 45,799 | Self reported | 11.00 | Postmenopause | BMI | 22.1-23.9 | NA | 0.96 (0.74-1.24) |  |
| 2010 | Harris | USA | Prospective cohort studies | NR | 620 | 45,799 | Self reported | 11.00 | Postmenopause | BMI | 24-27.4 | NA | 0.98 (0.73-1.29) |  |
| 2010 | Harris | USA | Prospective cohort studies | NR | 620 | 45,799 | Self reported | 11.00 | Postmenopause | BMI | ≥27.5 | NA | 0.81 (0.53-1.21) |  |
| 2010 | Harris | USA | Prospective cohort studies | NR | 620 | 45,799 | Self reported | 11.00 | Postmenopause | WC | <68.58 | ER+ | 1.00 | BMI, age, height, history of benign breast disease, family history of BC, age at menarche, age at first birth, parity, oral contraceptive use, alcohol consumption, and physical activity. |
| 2010 | Harris | USA | Prospective cohort studies | NR | 620 | 45,799 | Self reported | 11.00 | Postmenopause | WC | 68.58-73.03 | ER+ | 1.11 (0.80-1.53) |  |
| 2010 | Harris | USA | Prospective cohort studies | NR | 620 | 45,799 | Self reported | 11.00 | Postmenopause | WC | 73.66-78.11 | ER+ | 1.23 (0.88-1.73) |  |
| 2010 | Harris | USA | Prospective cohort studies | NR | 620 | 45,799 | Self reported | 11.00 | Postmenopause | WC | 78.74-86.36 | ER+ | 1.27 (0.88-1.83) |  |
| 2010 | Harris | USA | Prospective cohort studies | NR | 620 | 45,799 | Self reported | 11.00 | Postmenopause | WC | ≥86.995 | ER+ | 1.32 (0.83-2.11) |  |
| 2010 | Harris | USA | Prospective cohort studies | NR | 620 | 45,799 | Self reported | 11.00 | Postmenopause | HC | <92.075 | ER+ | 1.00 |  |
| 2010 | Harris | USA | Prospective cohort studies | NR | 620 | 45,799 | Self reported | 11.00 | Postmenopause | HC | 92.075-95.89 | ER+ | 0.94 (0.67-1.31) |  |
| 2010 | Harris | USA | Prospective cohort studies | NR | 620 | 45,799 | Self reported | 11.00 | Postmenopause | HC | 96.52-99.70 | ER+ | 1.10 (0.80-1.51) |  |
| 2010 | Harris | USA | Prospective cohort studies | NR | 620 | 45,799 | Self reported | 11.00 | Postmenopause | HC | 100.33-106.68 | ER+ | 1.11 (0.78-1.59) |  |
| 2010 | Harris | USA | Prospective cohort studies | NR | 620 | 45,799 | Self reported | 11.00 | Postmenopause | HC | ≥107.32 | ER+ | 1.11 (0.68-1.82) |  |
| 2010 | Harris | USA | Prospective cohort studies | NR | 620 | 45,799 | Self reported | 11.00 | Postmenopause | WHR | <0.73 | ER+ | 1.00 |  |
| 2010 | Harris | USA | Prospective cohort studies | NR | 620 | 45,799 | Self reported | 11.00 | Postmenopause | WHR | 0.73-0.75 | ER+ | 0.89 (0.65-1.21) |  |
| 2010 | Harris | USA | Prospective cohort studies | NR | 620 | 45,799 | Self reported | 11.00 | Postmenopause | WHR | 0.76-0.78 | ER+ | 0.96 (0.70-1.32) |  |
| 2010 | Harris | USA | Prospective cohort studies | NR | 620 | 45,799 | Self reported | 11.00 | Postmenopause | WHR | 0.79-0.83 | ER+ | 1.00 (0.74-1.36) |  |
| 2010 | Harris | USA | Prospective cohort studies | NR | 620 | 45,799 | Self reported | 11.00 | Postmenopause | WHR | ≥0.84 | ER+ | 1.07 (0.77-1.48) |  |
| 2010 | Harris | USA | Prospective cohort studies | NR | 620 | 45,799 | Self reported | 11.00 | Postmenopause | WC | <68.58 | ER- | 1.00 |  |
| 2010 | Harris | USA | Prospective cohort studies | NR | 620 | 45,799 | Self reported | 11.00 | Postmenopause | WC | 68.58-73.03 | ER- | 1.89 (0.95-3.78) |  |
| 2010 | Harris | USA | Prospective cohort studies | NR | 620 | 45,799 | Self reported | 11.00 | Postmenopause | WC | 73.66-78.11 | ER- | 1.94 (0.94-3.99) |  |
| 2010 | Harris | USA | Prospective cohort studies | NR | 620 | 45,799 | Self reported | 11.00 | Postmenopause | WC | 78.74-86.36 | ER- | 2.94 (1.43-6.04) |  |
| 2010 | Harris | USA | Prospective cohort studies | NR | 620 | 45,799 | Self reported | 11.00 | Postmenopause | WC | ≥86.995 | ER- | 2.75 (1.15-6.54) |  |
| 2010 | Harris | USA | Prospective cohort studies | NR | 620 | 45,799 | Self reported | 11.00 | Postmenopause | HC | <92.075 | ER- | 1.00 |  |
| 2010 | Harris | USA | Prospective cohort studies | NR | 620 | 45,799 | Self reported | 11.00 | Postmenopause | HC | 92.075-95.89 | ER- | 2.98 (1.56-5.73) |  |
| 2010 | Harris | USA | Prospective cohort studies | NR | 620 | 45,799 | Self reported | 11.00 | Postmenopause | HC | 96.52-99.70 | ER- | 2.30 (1.16-4.56) |  |
| 2010 | Harris | USA | Prospective cohort studies | NR | 620 | 45,799 | Self reported | 11.00 | Postmenopause | HC | 100.33-106.68 | ER- | 2.15 (1.03-4.46) |  |
| 2010 | Harris | USA | Prospective cohort studies | NR | 620 | 45,799 | Self reported | 11.00 | Postmenopause | HC | ≥107.32 | ER- | 2.40 (0.95-6.08) |  |
| 2010 | Harris | USA | Prospective cohort studies | NR | 620 | 45,799 | Self reported | 11.00 | Postmenopause | WHR | <0.73 | ER- | 1.00 |  |
| 2010 | Harris | USA | Prospective cohort studies | NR | 620 | 45,799 | Self reported | 11.00 | Postmenopause | WHR | 0.73-0.75 | ER- | 0.95 (0.50-1.79) |  |
| 2010 | Harris | USA | Prospective cohort studies | NR | 620 | 45,799 | Self reported | 11.00 | Postmenopause | WHR | 0.76-0.78 | ER- | 1.38 (0.77-2.48) |  |
| 2010 | Harris | USA | Prospective cohort studies | NR | 620 | 45,799 | Self reported | 11.00 | Postmenopause | WHR | 0.79-0.83 | ER- | 1.35 (0.76-2.41) |  |
| 2010 | Harris | USA | Prospective cohort studies | NR | 620 | 45,799 | Self reported | 11.00 | Postmenopause | WHR | ≥0.84 | ER- | 1.95 (1.10-3.46) |  |
| 2011 | Phipps | USA | Prospective cohort studies | 50-79 | 2,917 | 155,723 | Technicians measured | 7.90 | Postmenopause | BMI | <23.75 | NA | 1.00 | Age, education, income, family history of BC, race, recreational physical activity level, history of mammography (at baseline), and mammography during follow-up. |
| 2011 | Phipps | USA | Prospective cohort studies | 50-79 | 2,917 | 155,723 | Technicians measured | 7.90 | Postmenopause | BMI | 23.75-26.89 | NA | 1.19 (1.05-1.35) |  |
| 2011 | Phipps | USA | Prospective cohort studies | 50-79 | 2,917 | 155,723 | Technicians measured | 7.90 | Postmenopause | BMI | 26.90-31.04 | NA | 1.17 (1.03-1.33) |  |
| 2011 | Phipps | USA | Prospective cohort studies | 50-79 | 2,917 | 155,723 | Technicians measured | 7.90 | Postmenopause | BMI | >31.05 | NA | 1.39 (1.22-1.58) |  |
| 2011 | Phipps | USA | Prospective cohort studies | 50-79 | 2,917 | 155,723 | Technicians measured | 7.90 | Postmenopause | WHR | <0.758 | NA | 1.00 |  |
| 2011 | Phipps | USA | Prospective cohort studies | 50-79 | 2,917 | 155,723 | Technicians measured | 7.90 | Postmenopause | WHR | 0.758-0.80 | NA | 0.93 (0.82-1.05) |  |
| 2011 | Phipps | USA | Prospective cohort studies | 50-79 | 2,917 | 155,723 | Technicians measured | 7.90 | Postmenopause | WHR | 0.81-0.86 | NA | 1.04 (0.91-1.18) |  |
| 2011 | Phipps | USA | Prospective cohort studies | 50-79 | 2,917 | 155,723 | Technicians measured | 7.90 | Postmenopause | WHR | ≥0.86 | NA | 1.06 (0.93-1.21) |  |
| 2011 | Phipps | USA | Prospective cohort studies | 50-79 | 2,917 | 155,723 | Technicians measured | 7.90 | Postmenopause | WC | <76 | NA | 1.00 |  |
| 2011 | Phipps | USA | Prospective cohort studies | 50-79 | 2,917 | 155,723 | Technicians measured | 7.90 | Postmenopause | WC | 76-84.4 | NA | 1.14 (0.99-1.31) |  |
| 2011 | Phipps | USA | Prospective cohort studies | 50-79 | 2,917 | 155,723 | Technicians measured | 7.90 | Postmenopause | WC | 84.5-94.5 | NA | 1.14 (0.96-1.35) |  |
| 2011 | Phipps | USA | Prospective cohort studies | 50-79 | 2,917 | 155,723 | Technicians measured | 7.90 | Postmenopause | WC | ≥95 | NA | 1.34 (1.09-1.64) |  |
| 2011 | Phipps | USA | Prospective cohort studies | 50-79 | 2,917 | 155,723 | Technicians measured | 7.90 | Postmenopause | HC | <98 | NA | 1.00 |  |
| 2011 | Phipps | USA | Prospective cohort studies | 50-79 | 2,917 | 155,723 | Technicians measured | 7.90 | Postmenopause | HC | 98-104.4 | NA | 1.19 (1.03-1.37) |  |
| 2011 | Phipps | USA | Prospective cohort studies | 50-79 | 2,917 | 155,723 | Technicians measured | 7.90 | Postmenopause | HC | 104.5-112.9 | NA | 1.27 (1.08-1.51) |  |
| 2011 | Phipps | USA | Prospective cohort studies | 50-79 | 2,917 | 155,723 | Technicians measured | 7.90 | Postmenopause | HC | ≥113 | NA | 1.28 (1.03-1.58) |  |
| 2011 | Phipps | USA | Prospective cohort studies | 50-79 | 2,917 | 155,723 | Technicians measured | 7.90 | Postmenopause | BMI | <23.75 | Triple-negative | 1.00 |  |
| 2011 | Phipps | USA | Prospective cohort studies | 50-79 | 2,917 | 155,723 | Technicians measured | 7.90 | Postmenopause | BMI | 23.75-26.89 | Triple-negative | 0.99 (0.67-1.46) |  |
| 2011 | Phipps | USA | Prospective cohort studies | 50-79 | 2,917 | 155,723 | Technicians measured | 7.90 | Postmenopause | BMI | 26.90-31.04 | Triple-negative | 1.21 (0.83-1.77) |  |
| 2011 | Phipps | USA | Prospective cohort studies | 50-79 | 2,917 | 155,723 | Technicians measured | 7.90 | Postmenopause | BMI | >31.05 | Triple-negative | 1.35 (0.92-1.99) |  |
| 2011 | Phipps | USA | Prospective cohort studies | 50-79 | 2,917 | 155,723 | Technicians measured | 7.90 | Postmenopause | WHR | <0.758 | Triple-negative | 1.00 |  |
| 2011 | Phipps | USA | Prospective cohort studies | 50-79 | 2,917 | 155,723 | Technicians measured | 7.90 | Postmenopause | WHR | 0.758-0.80 | Triple-negative | 1.13 (0.79-1.62) |  |
| 2011 | Phipps | USA | Prospective cohort studies | 50-79 | 2,917 | 155,723 | Technicians measured | 7.90 | Postmenopause | WHR | 0.80-0.86 | Triple-negative | 0.99 (0.68-1.46) |  |
| 2011 | Phipps | USA | Prospective cohort studies | 50-79 | 2,917 | 155,723 | Technicians measured | 7.90 | Postmenopause | WHR | ≥0.86 | Triple-negative | 0.99 (0.66-1.49) |  |
| 2011 | Phipps | USA | Prospective cohort studies | 50-79 | 2,917 | 155,723 | Technicians measured | 7.90 | Postmenopause | WC | <76 | Triple-negative | 1.00 |  |
| 2011 | Phipps | USA | Prospective cohort studies | 50-79 | 2,917 | 155,723 | Technicians measured | 7.90 | Postmenopause | WC | 76-84.4 | Triple-negative | 0.61 (0.40-1.95) |  |
| 2011 | Phipps | USA | Prospective cohort studies | 50-79 | 2,917 | 155,723 | Technicians measured | 7.90 | Postmenopause | WC | 84.5-94.5 | Triple-negative | 0.66 (0.40-1.08) |  |
| 2011 | Phipps | USA | Prospective cohort studies | 50-79 | 2,917 | 155,723 | Technicians measured | 7.90 | Postmenopause | WC | ≥95 | Triple-negative | 0.66 (0.37-1.20) |  |
| 2011 | Phipps | USA | Prospective cohort studies | 50-79 | 2,917 | 155,723 | Technicians measured | 7.90 | Postmenopause | HC | <98 | Triple-negative | 1.00 |  |
| 2011 | Phipps | USA | Prospective cohort studies | 50-79 | 2,917 | 155,723 | Technicians measured | 7.90 | Postmenopause | HC | 98-104.4 | Triple-negative | 0.87 (0.56-1.33) |  |
| 2011 | Phipps | USA | Prospective cohort studies | 50-79 | 2,917 | 155,723 | Technicians measured | 7.90 | Postmenopause | HC | 104.5-112.9 | Triple-negative | 0.87 (0.52-1.43) |  |
| 2011 | Phipps | USA | Prospective cohort studies | 50-79 | 2,917 | 155,723 | Technicians measured | 7.90 | Postmenopause | HC | ≥113 | Triple-negative | 0.81 (0.44-1.50) |  |
| 2012 | Canchola | USA | Prospective cohort studies | 56-70 | 2,321 | 35,529 | Self reported | 10.10 | Postmenopause | BMI | <25 | ER+PR+ | 1.00 | Age at baseline, age at menarche, parity, age at first full-term pregnancy, history of benign breast biopsy, family history of BC, alcohol consumption, HRT, and height. |
| 2012 | Canchola | USA | Prospective cohort studies | 56-70 | 2,321 | 35,529 | Self reported | 10.10 | Postmenopause | BMI | 25-29 | ER+PR+ | 1.13 (1.00-1.28) |  |
| 2012 | Canchola | USA | Prospective cohort studies | 56-70 | 2,321 | 35,529 | Self reported | 10.10 | Postmenopause | BMI | ≥30 | ER+PR+ | 1.20 (1.03-1.40) |  |
| 2012 | Canchola | USA | Prospective cohort studies | 56-70 | 2,321 | 35,529 | Self reported | 10.10 | Postmenopause | WC | <76.2 | ER+PR+ | 1.00 |  |
| 2012 | Canchola | USA | Prospective cohort studies | 56-70 | 2,321 | 35,529 | Self reported | 10.10 | Postmenopause | WC | 76.2-81.28 | ER+PR+ | 1.31 (1.07-1.60) |  |
| 2012 | Canchola | USA | Prospective cohort studies | 56-70 | 2,321 | 35,529 | Self reported | 10.10 | Postmenopause | WC | 83.82-88.9 | ER+PR+ | 1.31 (1.06-1.62) |  |
| 2012 | Canchola | USA | Prospective cohort studies | 56-70 | 2,321 | 35,529 | Self reported | 10.10 | Postmenopause | WC | ≥91.44 | ER+PR+ | 1.33 (1.09-1.62) |  |
| 2012 | Canchola | USA | Prospective cohort studies | 56-70 | 2,321 | 35,529 | Self reported | 10.10 | Postmenopause | BMI | <25 | ER+ER- | 1.00 |  |
| 2012 | Canchola | USA | Prospective cohort studies | 56-70 | 2,321 | 35,529 | Self reported | 10.10 | Postmenopause | BMI | 25-29 | ER+ER- | 0.90 (0.69-1.18) |  |
| 2012 | Canchola | USA | Prospective cohort studies | 56-70 | 2,321 | 35,529 | Self reported | 10.10 | Postmenopause | BMI | ≥30 | ER+ER- | 0.84 (0.58-1.21) |  |
| 2012 | Canchola | USA | Prospective cohort studies | 56-70 | 2,321 | 35,529 | Self reported | 10.10 | Postmenopause | WC | <76.2 | ER+ER- | 1.00 |  |
| 2012 | Canchola | USA | Prospective cohort studies | 56-70 | 2,321 | 35,529 | Self reported | 10.10 | Postmenopause | WC | 76.2-81.28 | ER+ER- | 1.17 (0.77-1.79) |  |
| 2012 | Canchola | USA | Prospective cohort studies | 56-70 | 2,321 | 35,529 | Self reported | 10.10 | Postmenopause | WC | 83.82-88.9 | ER+ER- | 1.15 (0.74-1.79) |  |
| 2012 | Canchola | USA | Prospective cohort studies | 56-70 | 2,321 | 35,529 | Self reported | 10.10 | Postmenopause | WC | ≥91.44 | ER+ER- | 1.02 (0.67-1.57) |  |
| 2012 | Canchola | USA | Prospective cohort studies | 56-70 | 2,321 | 35,529 | Self reported | 10.10 | Postmenopause | BMI | <25 | ER-PR- | 1.00 |  |
| 2012 | Canchola | USA | Prospective cohort studies | 56-70 | 2,321 | 35,529 | Self reported | 10.10 | Postmenopause | BMI | 25-29 | ER-PR- | 1.13 (0.87-1.47) |  |
| 2012 | Canchola | USA | Prospective cohort studies | 56-70 | 2,321 | 35,529 | Self reported | 10.10 | Postmenopause | BMI | ≥30 | ER-PR- | 0.77 (0.53-1.12) |  |
| 2012 | Canchola | USA | Prospective cohort studies | 56-70 | 2,321 | 35,529 | Self reported | 10.10 | Postmenopause | WC | <76.2 | ER-PR- | 34.00 (1.00-0.00) |  |
| 2012 | Canchola | USA | Prospective cohort studies | 56-70 | 2,321 | 35,529 | Self reported | 10.10 | Postmenopause | WC | 76.2-81.28 | ER-PR- | 1.11 (0.70-1.76) |  |
| 2012 | Canchola | USA | Prospective cohort studies | 56-70 | 2,321 | 35,529 | Self reported | 10.10 | Postmenopause | WC | 83.82-88.9 | ER-PR- | 1.32 (0.82-2.10) |  |
| 2012 | Canchola | USA | Prospective cohort studies | 56-70 | 2,321 | 35,529 | Self reported | 10.10 | Postmenopause | WC | ≥91.44 | ER-PR- | 1.10 (0.70-1.73) |  |
| 2012 | Fagherazzi | France | Prospective cohort studies | 40-65 | 1,887 | 63,726 | Self reported | 5.4/10.1 | Premenopause | HC | <93 | ER+PR+ | 1.00 | Age, BMI, height, education, physical activity, energy intake, smoking, alcohol, nulliparity, age at first full-term pregnancy, breastfeeding, use of oral contraceptive, HRT use, age at menarche, family history of BC, history of benign breast disease, and mammography during follow up. |
| 2012 | Fagherazzi | France | Prospective cohort studies | 40-65 | 1,887 | 63,726 | Self reported | 5.4/10.1 | Premenopause | HC | 93-99 | ER+PR+ | 1.29 (0.91-1.83) |  |
| 2012 | Fagherazzi | France | Prospective cohort studies | 40-65 | 1,887 | 63,726 | Self reported | 5.4/10.1 | Premenopause | HC | ≥99 | ER+PR+ | 1.65 (1.04-2.62) |  |
| 2012 | Fagherazzi | France | Prospective cohort studies | 40-65 | 1,887 | 63,726 | Self reported | 5.4/10.1 | Premenopause | WC | <71 | ER+PR+ | 1.00 |  |
| 2012 | Fagherazzi | France | Prospective cohort studies | 40-65 | 1,887 | 63,726 | Self reported | 5.4/10.1 | Premenopause | WC | 71-77 | ER+PR+ | 1.24 (0.89-1.72) |  |
| 2012 | Fagherazzi | France | Prospective cohort studies | 40-65 | 1,887 | 63,726 | Self reported | 5.4/10.1 | Premenopause | WC | ≥77 | ER+PR+ | 0.71 (0.44-1.13) |  |
| 2012 | Fagherazzi | France | Prospective cohort studies | 40-65 | 1,887 | 63,726 | Self reported | 5.4/10.1 | Premenopause | WHR | <0.75 | ER+PR+ | 1.00 |  |
| 2012 | Fagherazzi | France | Prospective cohort studies | 40-65 | 1,887 | 63,726 | Self reported | 5.4/10.1 | Premenopause | WHR | 0.75-0.80 | ER+PR+ | 1.09 (0.80-1.50) |  |
| 2012 | Fagherazzi | France | Prospective cohort studies | 40-65 | 1,887 | 63,726 | Self reported | 5.4/10.1 | Premenopause | WHR | ≥0.8 | ER+PR+ | 0.70 (0.48-1.02) |  |
| 2012 | Fagherazzi | France | Prospective cohort studies | 40-65 | 1,887 | 63,726 | Self reported | 5.4/10.1 | Premenopause | BMI | <20 | ER+PR+ | 1.00 |  |
| 2012 | Fagherazzi | France | Prospective cohort studies | 40-65 | 1,887 | 63,726 | Self reported | 5.4/10.1 | Premenopause | BMI | 20-22.4 | ER+PR+ | 0.78 (0.54-1.13) |  |
| 2012 | Fagherazzi | France | Prospective cohort studies | 40-65 | 1,887 | 63,726 | Self reported | 5.4/10.1 | Premenopause | BMI | 22.5-24.9 | ER+PR+ | 0.73 (0.49-1.09) |  |
| 2012 | Fagherazzi | France | Prospective cohort studies | 40-65 | 1,887 | 63,726 | Self reported | 5.4/10.1 | Premenopause | BMI | 25-29.9 | ER+PR+ | 0.60 (0.38-0.96) |  |
| 2012 | Fagherazzi | France | Prospective cohort studies | 40-65 | 1,887 | 63,726 | Self reported | 5.4/10.1 | Premenopause | BMI | ≥30 | ER+PR+ | 0.40 (0.16-1.00) |  |
| 2012 | Fagherazzi | France | Prospective cohort studies | 40-65 | 1,887 | 63,726 | Self reported | 5.4/10.1 | Postmenopause | HC | <93 | ER+PR+ | 1.00 |  |
| 2012 | Fagherazzi | France | Prospective cohort studies | 40-65 | 1,887 | 63,726 | Self reported | 5.4/10.1 | Postmenopause | HC | 93-99 | ER+PR+ | 1.17 (0.97-1.40) |  |
| 2012 | Fagherazzi | France | Prospective cohort studies | 40-65 | 1,887 | 63,726 | Self reported | 5.4/10.1 | Postmenopause | HC | ≥99 | ER+PR+ | 1.07 (0.86-1.34) |  |
| 2012 | Fagherazzi | France | Prospective cohort studies | 40-65 | 1,887 | 63,726 | Self reported | 5.4/10.1 | Postmenopause | WC | <71 | ER+PR+ | 1.00 |  |
| 2012 | Fagherazzi | France | Prospective cohort studies | 40-65 | 1,887 | 63,726 | Self reported | 5.4/10.1 | Postmenopause | WC | 71-77 | ER+PR+ | 1.05 (0.87-1.27) |  |
| 2012 | Fagherazzi | France | Prospective cohort studies | 40-65 | 1,887 | 63,726 | Self reported | 5.4/10.1 | Postmenopause | WC | ≥77 | ER+PR+ | 1.01 (0.82-1.25) |  |
| 2012 | Fagherazzi | France | Prospective cohort studies | 40-65 | 1,887 | 63,726 | Self reported | 5.4/10.1 | Postmenopause | WHR | <0.75 | ER+PR+ | 1.00 |  |
| 2012 | Fagherazzi | France | Prospective cohort studies | 40-65 | 1,887 | 63,726 | Self reported | 5.4/10.1 | Postmenopause | WHR | 0.75-0.80 | ER+PR+ | 0.96 (0.81-1.15) |  |
| 2012 | Fagherazzi | France | Prospective cohort studies | 40-65 | 1,887 | 63,726 | Self reported | 5.4/10.1 | Postmenopause | WHR | ≥0.8 | ER+PR+ | 0.94 (0.79-1.13) |  |
| 2012 | Fagherazzi | France | Prospective cohort studies | 40-65 | 1,887 | 63,726 | Self reported | 5.4/10.1 | Postmenopause | BMI | <20 | ER+PR+ | 1.00 |  |
| 2012 | Fagherazzi | France | Prospective cohort studies | 40-65 | 1,887 | 63,726 | Self reported | 5.4/10.1 | Postmenopause | BMI | 20-22.4 | ER+PR+ | 1.11 (0.87-1.42) |  |
| 2012 | Fagherazzi | France | Prospective cohort studies | 40-65 | 1,887 | 63,726 | Self reported | 5.4/10.1 | Postmenopause | BMI | 22.5-24.9 | ER+PR+ | 1.23 (0.96-1.56) |  |
| 2012 | Fagherazzi | France | Prospective cohort studies | 40-65 | 1,887 | 63,726 | Self reported | 5.4/10.1 | Postmenopause | BMI | 25-29.9 | ER+PR+ | 1.37 (1.07-1.76) |  |
| 2012 | Fagherazzi | France | Prospective cohort studies | 40-65 | 1,887 | 63,726 | Self reported | 5.4/10.1 | Postmenopause | BMI | ≥30 | ER+PR+ | 1.63 (1.17-2.28) |  |
| 2012 | Fagherazzi | France | Prospective cohort studies | 40-65 | 1,887 | 63,726 | Self reported | 5.4/10.1 | Premenopause | HC | <93 | ER-PR- | 1.00 |  |
| 2012 | Fagherazzi | France | Prospective cohort studies | 40-65 | 1,887 | 63,726 | Self reported | 5.4/10.1 | Premenopause | HC | 93-99 | ER-PR- | 2.76 (1.28-5.96) |  |
| 2012 | Fagherazzi | France | Prospective cohort studies | 40-65 | 1,887 | 63,726 | Self reported | 5.4/10.1 | Premenopause | HC | ≥99 | ER-PR- | 3.13 (1.19-8.27) |  |
| 2012 | Fagherazzi | France | Prospective cohort studies | 40-65 | 1,887 | 63,726 | Self reported | 5.4/10.1 | Premenopause | WC | <71 | ER-PR- | 1.00 |  |
| 2012 | Fagherazzi | France | Prospective cohort studies | 40-65 | 1,887 | 63,726 | Self reported | 5.4/10.1 | Premenopause | WC | 71-77 | ER-PR- | 1.37 (0.67-2.79) |  |
| 2012 | Fagherazzi | France | Prospective cohort studies | 40-65 | 1,887 | 63,726 | Self reported | 5.4/10.1 | Premenopause | WC | ≥77 | ER-PR- | 1.10 (0.45-2.68) |  |
| 2012 | Fagherazzi | France | Prospective cohort studies | 40-65 | 1,887 | 63,726 | Self reported | 5.4/10.1 | Premenopause | WHR | <0.75 | ER-PR- | 1.00 |  |
| 2012 | Fagherazzi | France | Prospective cohort studies | 40-65 | 1,887 | 63,726 | Self reported | 5.4/10.1 | Premenopause | WHR | 0.75-0.80 | ER-PR- | 0.58 (0.29-1.17) |  |
| 2012 | Fagherazzi | France | Prospective cohort studies | 40-65 | 1,887 | 63,726 | Self reported | 5.4/10.1 | Premenopause | WHR | ≥0.8 | ER-PR- | 0.72 (0.37-1.42) |  |
| 2012 | Fagherazzi | France | Prospective cohort studies | 40-65 | 1,887 | 63,726 | Self reported | 5.4/10.1 | Premenopause | BMI | <20 | ER-PR- | 1.00 |  |
| 2012 | Fagherazzi | France | Prospective cohort studies | 40-65 | 1,887 | 63,726 | Self reported | 5.4/10.1 | Premenopause | BMI | 20-22.4 | ER-PR- | 0.93 (0.40-2.17) |  |
| 2012 | Fagherazzi | France | Prospective cohort studies | 40-65 | 1,887 | 63,726 | Self reported | 5.4/10.1 | Premenopause | BMI | 22.5-24.9 | ER-PR- | 1.40 (0.60-3.27) |  |
| 2012 | Fagherazzi | France | Prospective cohort studies | 40-65 | 1,887 | 63,726 | Self reported | 5.4/10.1 | Premenopause | BMI | 25-29.9 | ER-PR- | 1.18 (0.45-3.11) |  |
| 2012 | Fagherazzi | France | Prospective cohort studies | 40-65 | 1,887 | 63,726 | Self reported | 5.4/10.1 | Premenopause | BMI | ≥30 | ER-PR- | 1.45 (0.38-5.59) |  |
| 2012 | Fagherazzi | France | Prospective cohort studies | 40-65 | 1,887 | 63,726 | Self reported | 5.4/10.1 | Postmenopause | HC | <93 | ER-PR- | 1.00 |  |
| 2012 | Fagherazzi | France | Prospective cohort studies | 40-65 | 1,887 | 63,726 | Self reported | 5.4/10.1 | Postmenopause | HC | 93-99 | ER-PR- | 1.07 (0.76-1.51) |  |
| 2012 | Fagherazzi | France | Prospective cohort studies | 40-65 | 1,887 | 63,726 | Self reported | 5.4/10.1 | Postmenopause | HC | ≥99 | ER-PR- | 1.11 (0.72-1.71) |  |
| 2012 | Fagherazzi | France | Prospective cohort studies | 40-65 | 1,887 | 63,726 | Self reported | 5.4/10.1 | Postmenopause | WC | <71 | ER-PR- | 1.00 |  |
| 2012 | Fagherazzi | France | Prospective cohort studies | 40-65 | 1,887 | 63,726 | Self reported | 5.4/10.1 | Postmenopause | WC | 71-77 | ER-PR- | 0.90 (0.63-1.27) |  |
| 2012 | Fagherazzi | France | Prospective cohort studies | 40-65 | 1,887 | 63,726 | Self reported | 5.4/10.1 | Postmenopause | WC | ≥77 | ER-PR- | 0.85 (0.57-1.27) |  |
| 2012 | Fagherazzi | France | Prospective cohort studies | 40-65 | 1,887 | 63,726 | Self reported | 5.4/10.1 | Postmenopause | WHR | <0.75 | ER-PR- | 1.00 |  |
| 2012 | Fagherazzi | France | Prospective cohort studies | 40-65 | 1,887 | 63,726 | Self reported | 5.4/10.1 | Postmenopause | WHR | 0.75-0.80 | ER-PR- | 0.84 (0.60-1.18) |  |
| 2012 | Fagherazzi | France | Prospective cohort studies | 40-65 | 1,887 | 63,726 | Self reported | 5.4/10.1 | Postmenopause | WHR | ≥0.8 | ER-PR- | 0.91 (0.65-1.27) |  |
| 2012 | Fagherazzi | France | Prospective cohort studies | 40-65 | 1,887 | 63,726 | Self reported | 5.4/10.1 | Postmenopause | BMI | <20 | ER-PR- | 1.00 |  |
| 2012 | Fagherazzi | France | Prospective cohort studies | 40-65 | 1,887 | 63,726 | Self reported | 5.4/10.1 | Postmenopause | BMI | 20-22.4 | ER-PR- | 1.20 (0.77-1.87) |  |
| 2012 | Fagherazzi | France | Prospective cohort studies | 40-65 | 1,887 | 63,726 | Self reported | 5.4/10.1 | Postmenopause | BMI | 22.5-24.9 | ER-PR- | 1.00 (0.63-1.58) |  |
| 2012 | Fagherazzi | France | Prospective cohort studies | 40-65 | 1,887 | 63,726 | Self reported | 5.4/10.1 | Postmenopause | BMI | 25-29.9 | ER-PR- | 1.02 (0.63-1.65) |  |
| 2012 | Fagherazzi | France | Prospective cohort studies | 40-65 | 1,887 | 63,726 | Self reported | 5.4/10.1 | Postmenopause | BMI | ≥30 | ER-PR- | 0.77 (0.36-1.66) |  |
| 2013 | Fagherazzi | France | Prospective cohort studies | 40-65 | 7,247 | 63,798 | Self reported | 12.00 | Premenopausal | WC | <71 | NA | 1.00 | BMI, smoking, education, diabetes, alcohol intake, previous history of coronary heart disease, physical activity, dietary intake, use of oral contraceptives and use of hormone, height, and HRT (only for post-menopausal women). |
| 2013 | Fagherazzi | France | Prospective cohort studies | 40-65 | 7,247 | 63,798 | Self reported | 12.00 | Premenopausal | WC | 71-76 | NA | 1.12 (0.91-1.37) |  |
| 2013 | Fagherazzi | France | Prospective cohort studies | 40-65 | 7,247 | 63,798 | Self reported | 12.00 | Premenopausal | WC | 76-81 | NA | 1.16 (0.93-1.45) |  |
| 2013 | Fagherazzi | France | Prospective cohort studies | 40-65 | 7,247 | 63,798 | Self reported | 12.00 | Premenopausal | WC | 81-88 | NA | 1.03 (0.78-1.30) |  |
| 2013 | Fagherazzi | France | Prospective cohort studies | 40-65 | 7,247 | 63,798 | Self reported | 12.00 | Premenopausal | WC | ≥88 | NA | 0.98 (0.71-1.35) |  |
| 2013 | Fagherazzi | France | Prospective cohort studies | 40-65 | 7,247 | 63,798 | Self reported | 12.00 | Premenopausal | HC | <91 | NA | 1.00 |  |
| 2013 | Fagherazzi | France | Prospective cohort studies | 40-65 | 7,247 | 63,798 | Self reported | 12.00 | Premenopausal | HC | 91-95 | NA | 1.21 (0.98-1.50) |  |
| 2013 | Fagherazzi | France | Prospective cohort studies | 40-65 | 7,247 | 63,798 | Self reported | 12.00 | Premenopausal | HC | 95-100 | NA | 1.12 (0.89-1.41) |  |
| 2013 | Fagherazzi | France | Prospective cohort studies | 40-65 | 7,247 | 63,798 | Self reported | 12.00 | Premenopausal | HC | 100-105 | NA | 1.28 (1.00-1.64) |  |
| 2013 | Fagherazzi | France | Prospective cohort studies | 40-65 | 7,247 | 63,798 | Self reported | 12.00 | Premenopausal | HC | ≥105 | NA | 1.33 (0.98-1.80) |  |
| 2013 | Fagherazzi | France | Prospective cohort studies | 40-65 | 7,247 | 63,798 | Self reported | 12.00 | Premenopausal | BMI | <20.7 | NA | 0.96 (0.77-1.19) |  |
| 2013 | Fagherazzi | France | Prospective cohort studies | 40-65 | 7,247 | 63,798 | Self reported | 12.00 | Premenopausal | BMI | 20.7-22.4 | NA | 0.81 (0.65-1.01) |  |
| 2013 | Fagherazzi | France | Prospective cohort studies | 40-65 | 7,247 | 63,798 | Self reported | 12.00 | Premenopausal | BMI | 22.4-23.9 | NA | 1.00 |  |
| 2013 | Fagherazzi | France | Prospective cohort studies | 40-65 | 7,247 | 63,798 | Self reported | 12.00 | Premenopausal | BMI | 23.9-26.3 | NA | 0.78 (0.62-0.99) |  |
| 2013 | Fagherazzi | France | Prospective cohort studies | 40-65 | 7,247 | 63,798 | Self reported | 12.00 | Premenopausal | BMI | ≥26.3 | NA | 0.65 (0.50-0.85) |  |
| 2013 | Fagherazzi | France | Prospective cohort studies | 40-65 | 7,247 | 63,798 | Self reported | 12.00 | Postmenopause | WC | <71 | NA | 1.00 |  |
| 2013 | Fagherazzi | France | Prospective cohort studies | 40-65 | 7,247 | 63,798 | Self reported | 12.00 | Postmenopause | WC | 71-76 | NA | 1.06 (0.97-1.15) |  |
| 2013 | Fagherazzi | France | Prospective cohort studies | 40-65 | 7,247 | 63,798 | Self reported | 12.00 | Postmenopause | WC | 76-81 | NA | 1.13 (1.03-1.23) |  |
| 2013 | Fagherazzi | France | Prospective cohort studies | 40-65 | 7,247 | 63,798 | Self reported | 12.00 | Postmenopause | WC | 81-88 | NA | 1.06 (0.96-1.16) |  |
| 2013 | Fagherazzi | France | Prospective cohort studies | 40-65 | 7,247 | 63,798 | Self reported | 12.00 | Postmenopause | WC | ≥88 | NA | 1.21 (1.09-1.35) |  |
| 2013 | Fagherazzi | France | Prospective cohort studies | 40-65 | 7,247 | 63,798 | Self reported | 12.00 | Postmenopause | HC | <91 | NA | 1.00 |  |
| 2013 | Fagherazzi | France | Prospective cohort studies | 40-65 | 7,247 | 63,798 | Self reported | 12.00 | Postmenopause | HC | 91-95 | NA | 1.01 (0.92-1.11) |  |
| 2013 | Fagherazzi | France | Prospective cohort studies | 40-65 | 7,247 | 63,798 | Self reported | 12.00 | Postmenopause | HC | 95-100 | NA | 1.04 (0.96-1.14) |  |
| 2013 | Fagherazzi | France | Prospective cohort studies | 40-65 | 7,247 | 63,798 | Self reported | 12.00 | Postmenopause | HC | 100-105 | NA | 1.06 (0.96-1.16) |  |
| 2013 | Fagherazzi | France | Prospective cohort studies | 40-65 | 7,247 | 63,798 | Self reported | 12.00 | Postmenopause | HC | ≥105 | NA | 1.11 (0.99-2.24) |  |
| 2013 | Fagherazzi | France | Prospective cohort studies | 40-65 | 7,247 | 63,798 | Self reported | 12.00 | Postmenopause | BMI | <20.7 | NA | 1.05 (0.96-1.14) |  |
| 2013 | Fagherazzi | France | Prospective cohort studies | 40-65 | 7,247 | 63,798 | Self reported | 12.00 | Postmenopause | BMI | 20.7-22.4 | NA | 1.01 (0.93-1.10) |  |
| 2013 | Fagherazzi | France | Prospective cohort studies | 40-65 | 7,247 | 63,798 | Self reported | 12.00 | Postmenopause | BMI | 22.4-23.9 | NA | 1.00 |  |
| 2013 | Fagherazzi | France | Prospective cohort studies | 40-65 | 7,247 | 63,798 | Self reported | 12.00 | Postmenopause | BMI | 23.9-26.3 | NA | 1.02 (0.94-1.11) |  |
| 2013 | Fagherazzi | France | Prospective cohort studies | 40-65 | 7,247 | 63,798 | Self reported | 12.00 | Postmenopause | BMI | ≥26.3 | NA | 1.02 (0.93-1.12) |  |
| 2014 | Catsburg | Canada | Prospective cohort studies | NR | 1,097 | 3,320 | Self reported | 11.40 | Premenopause | BMI | <18.5 | NA | 0.79 (0.39-1.61) | Age, alcohol, physical activity, use of oral contraceptives, HRT, number of live births, age at menarche, age at first live birth, family history of BC. |
| 2014 | Catsburg | Canada | Prospective cohort studies | NR | 1,097 | 3,320 | Self reported | 11.40 | Premenopause | BMI | 18.5-24.99 | NA | 1.00 |  |
| 2014 | Catsburg | Canada | Prospective cohort studies | NR | 1,097 | 3,320 | Self reported | 11.40 | Premenopause | BMI | 24.5-29.99 | NA | 1.06 (0.82-1.38) |  |
| 2014 | Catsburg | Canada | Prospective cohort studies | NR | 1,097 | 3,320 | Self reported | 11.40 | Premenopause | BMI | ≥30 | NA | 0.97 (0.66-1.43) |  |
| 2014 | Catsburg | Canada | Prospective cohort studies | NR | 1,097 | 3,320 | Self reported | 11.40 | Premenopause | WC | <73 | NA | 1.00 |  |
| 2014 | Catsburg | Canada | Prospective cohort studies | NR | 1,097 | 3,320 | Self reported | 11.40 | Premenopause | WC | 73-78.7 | NA | 1.11 (0.83-1.49) |  |
| 2014 | Catsburg | Canada | Prospective cohort studies | NR | 1,097 | 3,320 | Self reported | 11.40 | Premenopause | WC | 78.7-84.8 | NA | 1.08 (0.80-1.47) |  |
| 2014 | Catsburg | Canada | Prospective cohort studies | NR | 1,097 | 3,320 | Self reported | 11.40 | Premenopause | WC | 84.8-92.7 | NA | 1.50 (1.06-2.12) |  |
| 2014 | Catsburg | Canada | Prospective cohort studies | NR | 1,097 | 3,320 | Self reported | 11.40 | Premenopause | WC | >92.7 | NA | 0.84 (0.59-1.21) |  |
| 2014 | Catsburg | Canada | Prospective cohort studies | NR | 1,097 | 3,320 | Self reported | 11.40 | Premenopause | WHR | <0.76 | NA | 1.00 |  |
| 2014 | Catsburg | Canada | Prospective cohort studies | NR | 1,097 | 3,320 | Self reported | 11.40 | Premenopause | WHR | 0.76-0.79 | NA | 0.94 (0.71-1.25) |  |
| 2014 | Catsburg | Canada | Prospective cohort studies | NR | 1,097 | 3,320 | Self reported | 11.40 | Premenopause | WHR | 0.79-0.83 | NA | 1.02 (0.76-1.39) |  |
| 2014 | Catsburg | Canada | Prospective cohort studies | NR | 1,097 | 3,320 | Self reported | 11.40 | Premenopause | WHR | 0.83-0.88 | NA | 0.88 (0.62-1.24) |  |
| 2014 | Catsburg | Canada | Prospective cohort studies | NR | 1,097 | 3,320 | Self reported | 11.40 | Premenopause | WHR | >0.88 | NA | 0.74 (0.49-1.11) |  |
| 2014 | Catsburg | Canada | Prospective cohort studies | NR | 1,097 | 3,320 | Self reported | 11.40 | Postmenopause | BMI | <18.5 | NA | 1.40 (0.75-2.62) |  |
| 2014 | Catsburg | Canada | Prospective cohort studies | NR | 1,097 | 3,320 | Self reported | 11.40 | Postmenopause | BMI | 18.5-24.99 | NA | 1.00 |  |
| 2014 | Catsburg | Canada | Prospective cohort studies | NR | 1,097 | 3,320 | Self reported | 11.40 | Postmenopause | BMI | 24.5-29.99 | NA | 1.20 (0.99-1.52) |  |
| 2014 | Catsburg | Canada | Prospective cohort studies | NR | 1,097 | 3,320 | Self reported | 11.40 | Postmenopause | BMI | ≥30 | NA | 1.24 (0.90-1.71) |  |
| 2014 | Catsburg | Canada | Prospective cohort studies | NR | 1,097 | 3,320 | Self reported | 11.40 | Postmenopause | WC | <73 | NA | 1.00 |  |
| 2014 | Catsburg | Canada | Prospective cohort studies | NR | 1,097 | 3,320 | Self reported | 11.40 | Postmenopause | WC | 73-78.7 | NA | 1.20 (0.84-1.71) |  |
| 2014 | Catsburg | Canada | Prospective cohort studies | NR | 1,097 | 3,320 | Self reported | 11.40 | Postmenopause | WC | 78.7-84.8 | NA | 1.28 (0.91-1.78) |  |
| 2014 | Catsburg | Canada | Prospective cohort studies | NR | 1,097 | 3,320 | Self reported | 11.40 | Postmenopause | WC | 84.8-92.7 | NA | 1.29 (0.93-1.80) |  |
| 2014 | Catsburg | Canada | Prospective cohort studies | NR | 1,097 | 3,320 | Self reported | 11.40 | Postmenopause | WC | >92.7 | NA | 1.30 (0.92-1.82) |  |
| 2014 | Catsburg | Canada | Prospective cohort studies | NR | 1,097 | 3,320 | Self reported | 11.40 | Postmenopause | WHR | <0.76 | NA | 1.00 |  |
| 2014 | Catsburg | Canada | Prospective cohort studies | NR | 1,097 | 3,320 | Self reported | 11.40 | Postmenopause | WHR | 0.76-0.79 | NA | 0.84 (0.60-1.17) |  |
| 2014 | Catsburg | Canada | Prospective cohort studies | NR | 1,097 | 3,320 | Self reported | 11.40 | Postmenopause | WHR | 0.79-0.83 | NA | 0.86 (0.62-1.18) |  |
| 2014 | Catsburg | Canada | Prospective cohort studies | NR | 1,097 | 3,320 | Self reported | 11.40 | Postmenopause | WHR | 0.83-0.88 | NA | 0.93 (0.67-1.28) |  |
| 2014 | Catsburg | Canada | Prospective cohort studies | NR | 1,097 | 3,320 | Self reported | 11.40 | Postmenopause | WHR | >0.88 | NA | 1.08 (0.78-1.49) |  |
| 2014 | Gaudet | USA | Prospective cohort studies | NR | 1,088 | 28,965 | Self reported | 11.58 | Postmenopause | WC | 39-74 | NA | 1.00 | Age, height, education, parity, age at first birth, smoking and alcohol use, race, family history of breast cancer, oral contraceptive use, diabetes, age at menopause, exercise, benign breast disease, recent mammography screening, and HRT. |
| 2014 | Gaudet | USA | Prospective cohort studies | NR | 1,088 | 28,965 | Self reported | 11.58 | Postmenopause | WC | 75-79 | NA | 0.82 (0.65-1.05) |  |
| 2014 | Gaudet | USA | Prospective cohort studies | NR | 1,088 | 28,965 | Self reported | 11.58 | Postmenopause | WC | 80-87 | NA | 0.76 (0.62-0.94) |  |
| 2014 | Gaudet | USA | Prospective cohort studies | NR | 1,088 | 28,965 | Self reported | 11.58 | Postmenopause | WC | 88-96 | NA | 0.96 (0.77-1.19) |  |
| 2014 | Gaudet | USA | Prospective cohort studies | NR | 1,088 | 28,965 | Self reported | 11.58 | Postmenopause | WC | 97-139 | NA | 0.85 (0.65-1.12) |  |
| 2014 | Gaudet | USA | Prospective cohort studies | NR | 1,088 | 28,965 | Self reported | 11.58 | Postmenopause | BMI | <25 | NA | 1.00 |  |
| 2014 | Gaudet | USA | Prospective cohort studies | NR | 1,088 | 28,965 | Self reported | 11.58 | Postmenopause | BMI | 25-29.9 | NA | 1.26 (1.07-1.48) |  |
| 2014 | Gaudet | USA | Prospective cohort studies | NR | 1,088 | 28,965 | Self reported | 11.58 | Postmenopause | BMI | ≥30 | NA | 1.40 (1.10-1.78) |  |
| 2014 | Gaudet | USA | Prospective cohort studies | NR | 1,088 | 28,965 | Self reported | 11.58 | Postmenopause | WC | <79 | ER+ | 1.00 |  |
| 2014 | Gaudet | USA | Prospective cohort studies | NR | 1,088 | 28,965 | Self reported | 11.58 | Postmenopause | WC | 80-87 | ER+ | 0.82 (0.67-1.02) |  |
| 2014 | Gaudet | USA | Prospective cohort studies | NR | 1,088 | 28,965 | Self reported | 11.58 | Postmenopause | WC | ≥88 | ER+ | 1.09 (0.95-1.15) |  |
| 2014 | Gaudet | USA | Prospective cohort studies | NR | 1,088 | 28,965 | Self reported | 11.58 | Postmenopause | BMI | <25 | ER+ | 1.00 |  |
| 2014 | Gaudet | USA | Prospective cohort studies | NR | 1,088 | 28,965 | Self reported | 11.58 | Postmenopause | BMI | 25-29.9 | ER+ | 1.28 (1.06-1.54) |  |
| 2014 | Gaudet | USA | Prospective cohort studies | NR | 1,088 | 28,965 | Self reported | 11.58 | Postmenopause | BMI | ≥30 | ER+ | 1.41 (1.08-1.85) |  |
| 2014 | Gaudet | USA | Prospective cohort studies | NR | 1,088 | 28,965 | Self reported | 11.58 | Postmenopause | WC | <79 | ER- | 1.00 |  |
| 2014 | Gaudet | USA | Prospective cohort studies | NR | 1,088 | 28,965 | Self reported | 11.58 | Postmenopause | WC | 80-87 | ER- | 1.07 (0.68-1.69) |  |
| 2014 | Gaudet | USA | Prospective cohort studies | NR | 1,088 | 28,965 | Self reported | 11.58 | Postmenopause | WC | ≥88 | ER- | 0.75 (0.48-1.17) |  |
| 2014 | Gaudet | USA | Prospective cohort studies | NR | 1,088 | 28,965 | Self reported | 11.58 | Postmenopause | BMI | <25 | ER- | 1.00 |  |
| 2014 | Gaudet | USA | Prospective cohort studies | NR | 1,088 | 28,965 | Self reported | 11.58 | Postmenopause | BMI | 25-29.9 | ER- | 0.96 (0.65-1.43) |  |
| 2014 | Gaudet | USA | Prospective cohort studies | NR | 1,088 | 28,965 | Self reported | 11.58 | Postmenopause | BMI | ≥30 | ER- | 0.61 (0.35-1.08) |  |
| 2015 | White | USA | Prospective cohort studies | 35-74 | 2,009 | 50,884 | Technicians measured | 5.40 | Postmenopause | BMI | <18.5 | ER+PR+ | 0.61 (0.25-1.48) | Age, race, education, age at menarche, breastfeeding history, age at first birth, parity, HRT, smoking history, alcohol consumption, and physical activity. |
| 2015 | White | USA | Prospective cohort studies | 35-74 | 2,009 | 50,884 | Technicians measured | 5.40 | Postmenopause | BMI | 18.5-24.9 | ER+PR+ | 1.00 |  |
| 2015 | White | USA | Prospective cohort studies | 35-74 | 2,009 | 50,884 | Technicians measured | 5.40 | Postmenopause | BMI | 25-29 | ER+PR+ | 1.45 (1.23-1.71) |  |
| 2015 | White | USA | Prospective cohort studies | 35-74 | 2,009 | 50,884 | Technicians measured | 5.40 | Postmenopause | BMI | 30-34.9 | ER+PR+ | 1.42 (1.16-1.75) |  |
| 2015 | White | USA | Prospective cohort studies | 35-74 | 2,009 | 50,884 | Technicians measured | 5.40 | Postmenopause | BMI | >35 | ER+PR+ | 1.49 (1.18-1.88) |  |
| 2015 | White | USA | Prospective cohort studies | 35-74 | 2,009 | 50,884 | Technicians measured | 5.40 | Postmenopause | WC | ≤80 | ER+PR+ | 1.00 |  |
| 2015 | White | USA | Prospective cohort studies | 35-74 | 2,009 | 50,884 | Technicians measured | 5.40 | Postmenopause | WC | 81-88 | ER+PR+ | 1.63 (1.36-1.96) |  |
| 2015 | White | USA | Prospective cohort studies | 35-74 | 2,009 | 50,884 | Technicians measured | 5.40 | Postmenopause | WC | >88 | ER+PR+ | 1.53 (1.29-1.81) |  |
| 2015 | White | USA | Prospective cohort studies | 35-74 | 2,009 | 50,884 | Technicians measured | 5.40 | Postmenopause | WHR | <0.75 | ER+PR+ | 1.00 |  |
| 2015 | White | USA | Prospective cohort studies | 35-74 | 2,009 | 50,884 | Technicians measured | 5.40 | Postmenopause | WHR | 0.75-0.79 | ER+PR+ | 1.08 (0.88-1.32) |  |
| 2015 | White | USA | Prospective cohort studies | 35-74 | 2,009 | 50,884 | Technicians measured | 5.40 | Postmenopause | WHR | 0.8-0.85 | ER+PR+ | 1.12 (0.93-1.35) |  |
| 2015 | White | USA | Prospective cohort studies | 35-74 | 2,009 | 50,884 | Technicians measured | 5.40 | Postmenopause | WHR | ≥0.86 | ER+PR+ | 1.41 (1.16-1.71) |  |
| 2015 | White | USA | Prospective cohort studies | 35-74 | 2,009 | 50,884 | Technicians measured | 5.40 | Postmenopause | BMI | <18.5 | ER-/PR- | 2.65 (1.06-6.58) |  |
| 2015 | White | USA | Prospective cohort studies | 35-74 | 2,009 | 50,884 | Technicians measured | 5.40 | Postmenopause | BMI | 18.5-24.9 | ER-/PR- | 1.00 |  |
| 2015 | White | USA | Prospective cohort studies | 35-74 | 2,009 | 50,884 | Technicians measured | 5.40 | Postmenopause | BMI | 25-29 | ER-/PR- | 0.98 (0.68-1.41) |  |
| 2015 | White | USA | Prospective cohort studies | 35-74 | 2,009 | 50,884 | Technicians measured | 5.40 | Postmenopause | BMI | 30-34.9 | ER-/PR- | 1.15 (0.75-1.76) |  |
| 2015 | White | USA | Prospective cohort studies | 35-74 | 2,009 | 50,884 | Technicians measured | 5.40 | Postmenopause | BMI | >35 | ER-/PR- | 0.97 (0.58-1.62) |  |
| 2015 | White | USA | Prospective cohort studies | 35-74 | 2,009 | 50,884 | Technicians measured | 5.40 | Postmenopause | WC | ≤80 | ER-/PR- | 1.00 |  |
| 2015 | White | USA | Prospective cohort studies | 35-74 | 2,009 | 50,884 | Technicians measured | 5.40 | Postmenopause | WC | 81-88 | ER-/PR- | 1.28 (0.87-1.89) |  |
| 2015 | White | USA | Prospective cohort studies | 35-74 | 2,009 | 50,884 | Technicians measured | 5.40 | Postmenopause | WC | >88 | ER-/PR- | 1.18 (0.83-1.67) |  |
| 2015 | White | USA | Prospective cohort studies | 35-74 | 2,009 | 50,884 | Technicians measured | 5.40 | Postmenopause | WHR | <0.75 | ER-/PR- | 1.00 |  |
| 2015 | White | USA | Prospective cohort studies | 35-74 | 2,009 | 50,884 | Technicians measured | 5.40 | Postmenopause | WHR | 0.75-0.79 | ER-/PR- | 1.07 (0.69-1.66) |  |
| 2015 | White | USA | Prospective cohort studies | 35-74 | 2,009 | 50,884 | Technicians measured | 5.40 | Postmenopause | WHR | 0.8-0.85 | ER-/PR- | 1.16 (0.77-1.73) |  |
| 2015 | White | USA | Prospective cohort studies | 35-74 | 2,009 | 50,884 | Technicians measured | 5.40 | Postmenopause | WHR | ≥0.86 | ER-/PR- | 1.46 (0.97-2.21) |  |
| 2015 | White | USA | Prospective cohort studies | 35-74 | 2,009 | 50,884 | Technicians measured | 5.40 | Postmenopause | WC | ≤80 | NA | 1.00 |  |
| 2015 | White | USA | Prospective cohort studies | 35-74 | 2,009 | 50,884 | Technicians measured | 5.40 | Postmenopause | WC | 81-88 | NA | 1.16 (1.01-1.35) |  |
| 2015 | White | USA | Prospective cohort studies | 35-74 | 2,009 | 50,884 | Technicians measured | 5.40 | Postmenopause | WC | >88 | NA | 1.30 (1.10-1.54) |  |
| 2015 | White | USA | Prospective cohort studies | 35-74 | 2,009 | 50,884 | Technicians measured | 5.40 | Postmenopause | WHR | <0.75 | NA | 1.00 |  |
| 2015 | White | USA | Prospective cohort studies | 35-74 | 2,009 | 50,884 | Technicians measured | 5.40 | Postmenopause | WHR | 0.75-0.79 | NA | 0.97 (0.82-1.13) |  |
| 2015 | White | USA | Prospective cohort studies | 35-74 | 2,009 | 50,884 | Technicians measured | 5.40 | Postmenopause | WHR | 0.8-0.85 | NA | 1.06 (0.91-1.23) |  |
| 2015 | White | USA | Prospective cohort studies | 35-74 | 2,009 | 50,884 | Technicians measured | 5.40 | Postmenopause | WHR | ≥0.86 | NA | 1.28 (1.10-1.49) |  |
| 2015 | White | USA | Prospective cohort studies | 35-74 | 2,009 | 50,884 | Technicians measured | 5.40 | Premenopause | WC | ≤80 | NA | 1.00 |  |
| 2015 | White | USA | Prospective cohort studies | 35-74 | 2,009 | 50,884 | Technicians measured | 5.40 | Premenopause | WC | 81-88 | NA | 1.56 (1.19-2.04) |  |
| 2015 | White | USA | Prospective cohort studies | 35-74 | 2,009 | 50,884 | Technicians measured | 5.40 | Premenopause | WC | >88 | NA | 1.30 (0.91-1.87) |  |
| 2015 | White | USA | Prospective cohort studies | 35-74 | 2,009 | 50,884 | Technicians measured | 5.40 | Premenopause | WHR | <0.75 | NA | 1.00 |  |
| 2015 | White | USA | Prospective cohort studies | 35-74 | 2,009 | 50,884 | Technicians measured | 5.40 | Premenopause | WHR | 0.75-0.79 | NA | 1.11 (0.84-1.46) |  |
| 2015 | White | USA | Prospective cohort studies | 35-74 | 2,009 | 50,884 | Technicians measured | 5.40 | Premenopause | WHR | 0.8-0.85 | NA | 1.10 (0.84-1.45) |  |
| 2015 | White | USA | Prospective cohort studies | 35-74 | 2,009 | 50,884 | Technicians measured | 5.40 | Premenopause | WHR | ≥0.86 | NA | 1.26 (0.91-1.73) |  |
| 2015 | Bellocco | Swedish | Prospective cohort studies | 16-84 | 609 | 19,196 | Self reported | 13.20 | Postmenopause | BMI | 18.5-25 | NA | 1.00 | Age at enrollment, cigarette smoking status, alcohol drinking, use of vitamin and mineral supplements, education level, contraceptive pill use, HRT, age at menarche, number of children, age at first full-term pregnancy, and childlessness. |
| 2015 | Bellocco | Swedish | Prospective cohort studies | 16-84 | 609 | 19,196 | Self reported | 13.20 | Postmenopause | BMI | 25-30 | NA | 1.20 (0.97-1.48) |  |
| 2015 | Bellocco | Swedish | Prospective cohort studies | 16-84 | 609 | 19,196 | Self reported | 13.20 | Postmenopause | BMI | ≥30 | NA | 1.58 (1.16-2.16) |  |
| 2015 | Bellocco | Swedish | Prospective cohort studies | 16-84 | 609 | 19,196 | Self reported | 13.20 | Postmenopause | WC | <80 | NA | 1.00 |  |
| 2015 | Bellocco | Swedish | Prospective cohort studies | 16-84 | 609 | 19,196 | Self reported | 13.20 | Postmenopause | WC | 80-88 | NA | 1.10 (0.87-1.40) |  |
| 2015 | Bellocco | Swedish | Prospective cohort studies | 16-84 | 609 | 19,196 | Self reported | 13.20 | Postmenopause | WC | ≥88 | NA | 1.27 (0.99-1.63) |  |
| 2015 | Bellocco | Swedish | Prospective cohort studies | 16-84 | 609 | 19,196 | Self reported | 13.20 | Postmenopause | WHR | ≤0.8 | NA | 1.00 |  |
| 2015 | Bellocco | Swedish | Prospective cohort studies | 16-84 | 609 | 19,196 | Self reported | 13.20 | Postmenopause | WHR | >0.8 | NA | 1.15 (0.94-1.41) |  |
| 2015 | Harding, | Australian | Prospective cohort studies | NR | 1,323 | 15,336 | Technicians measured | 16.00 | NA | BMI | NA | NA | 1.06 (1.01-1.12) | Baseline smoking status, education, and study cohort using age as the timescale. |
| 2015 | Harding, | Australian | Prospective cohort studies | NR | 1,323 | 15,336 | Technicians measured | 16.00 | NA | WC | NA | NA | 1.06 (1.01-1.12) |  |
| 2015 | Harding, | Australian | Prospective cohort studies | NR | 1,323 | 15,336 | Technicians measured | 16.00 | NA | HC | NA | NA | 1.09 (1.03-1.15) |  |
| 2015 | Harding, | Australian | Prospective cohort studies | NR | 1,323 | 15,336 | Technicians measured | 16.00 | NA | WHR | NA | NA | 1.01 (0.95-1.07) |  |
| 2016 | Liu | China | Prospective cohort studies | 40-70 | 174 | 68,253 | Technicians measured | 15.10 | Premenopause | WHR | 0.79-0.83 | NA | 1.04 (0.97-1.10) | Education, total energy intake, total vegetable and fruit intake, total meat intake, leisure-time physical activity, alcohol consumption, HRT, menopausal status, spouse smoking exposure, parity, family history of cancer, and BMI. |
| 2016 | Liu | China | Prospective cohort studies | 40-70 | 174 | 68,253 | Technicians measured | 15.10 | Postmenopause | WHR | 0.79-0.83 | NA | 1.05 (0.96-1.15) |  |
| 2016 | Liu | China | Prospective cohort studies | 40-70 | 174 | 68,253 | Technicians measured | 15.10 | NA | WHR | 0.79-0.83 | NA | 1.02 (0.94-1.11) |  |
| 2016 | Liu | China | Prospective cohort studies | 40-70 | 174 | 68,253 | Technicians measured | 15.10 | Premenopause | BMI | <18.5, ≥18.5 | NA | 1.02 (0.89-1.17) |  |
| 2016 | Liu | China | Prospective cohort studies | 40-70 | 174 | 68,253 | Technicians measured | 15.10 | Postmenopause | BMI | <18.5, ≥18.5 | NA | 1.45 (1.28-1.63) |  |
| 2016 | Liu | China | Prospective cohort studies | 40-70 | 174 | 68,253 | Technicians measured | 15.10 | NA | BMI | <18.5, ≥18.5 | NA | 1.23 (1.12-1.34) |  |
| 2018 | Al-Ajmi | England | Prospective cohort studies | 39-71 | 3,378 | 273,467 | Technicians measured | 6.20 | Premenopausal | BMI | 18.5–24.9 | NA | 1.00 | Age, family history of BC, and deprivation score. |
| 2018 | Al-Ajmi | England | Prospective cohort studies | 39-71 | 3,378 | 273,467 | Technicians measured | 6.90 | Premenopausal | BMI | 25–29.9 | NA | 0.84 (0.70-1.00) |  |
| 2018 | Al-Ajmi | England | Prospective cohort studies | 39-71 | 3,378 | 273,467 | Technicians measured | 6.90 | Premenopausal | BMI | ≥30 | NA | 0.73 (0.59-0.92) |  |
| 2018 | Al-Ajmi | England | Prospective cohort studies | 39-71 | 3,378 | 273,467 | Technicians measured | 6.90 | Premenopausal | WHR | ≤0.80 | NA | 1.00 |  |
| 2018 | Al-Ajmi | England | Prospective cohort studies | 39-71 | 3,378 | 273,467 | Technicians measured | 6.90 | Premenopausal | WHR | 0.81–0.85 | NA | 0.83 (0.68-1.01) |  |
| 2018 | Al-Ajmi | England | Prospective cohort studies | 39-71 | 3,378 | 273,467 | Technicians measured | 6.90 | Premenopausal | WHR | >0.85 | NA | 0.74 (0.60-0.92) |  |
| 2018 | Al-Ajmi | England | Prospective cohort studies | 39-71 | 3,378 | 273,467 | Technicians measured | 6.90 | Postmenopause | BMI | 18.5–24.9 | NA | 1.00 |  |
| 2018 | Al-Ajmi | England | Prospective cohort studies | 39-71 | 3,378 | 273,467 | Technicians measured | 6.90 | Postmenopause | BMI | 25–29.9 | NA | 1.10 (0.99-1.23) |  |
| 2018 | Al-Ajmi | England | Prospective cohort studies | 39-71 | 3,378 | 273,467 | Technicians measured | 6.90 | Postmenopause | BMI | ≥30 | NA | 1.24 (1.10-1.40) |  |
| 2018 | Al-Ajmi | England | Prospective cohort studies | 39-71 | 3,378 | 273,467 | Technicians measured | 6.90 | Postmenopause | WHR | ≤0.80 | NA | 1.00 |  |
| 2018 | Al-Ajmi | England | Prospective cohort studies | 39-71 | 3,378 | 273,467 | Technicians measured | 6.90 | Postmenopause | WHR | 0.81–0.85 | NA | 1.01 (0.90-1.14) |  |
| 2018 | Al-Ajmi | England | Prospective cohort studies | 39-71 | 3,378 | 273,467 | Technicians measured | 6.90 | Postmenopause | WHR | >0.85 | NA | 1.07 (0.96-1.20) |  |
| 2019 | Taleban | Isfahan | Prospective cohort studies | ＞18 | 1,191 | 7,893 | Technicians measured | NA | Premenopause | BMI | <18.5 | NA | 1.06 (0.14-5.74) | Age, age of menarche, age at first live birth, number of live births, years of schooling, family history of BC, oral contraceptive pill usage, HRT, alcohol consumption, and smoking. |
| 2019 | Taleban | Isfahan | Prospective cohort studies | ＞18 | 1,191 | 7,893 | Technicians measured | NA | Premenopause | BMI | 18-25 | NA | 1.00 |  |
| 2019 | Taleban | Isfahan | Prospective cohort studies | ＞18 | 1,191 | 7,893 | Technicians measured | NA | Premenopause | BMI | 25-30 | NA | 1.01 (0.73-1.38) |  |
| 2019 | Taleban | Isfahan | Prospective cohort studies | ＞18 | 1,191 | 7,893 | Technicians measured | NA | Premenopause | BMI | ≥30 | NA | 0.58 (0.37-0.89) |  |
| 2019 | Taleban | Isfahan | Prospective cohort studies | ＞18 | 1,191 | 7,893 | Technicians measured | NA | Premenopause | WHR | <0.77 | NA | 1.00 |  |
| 2019 | Taleban | Isfahan | Prospective cohort studies | ＞18 | 1,191 | 7,893 | Technicians measured | NA | Premenopause | WHR | 0.77-0.81 | NA | 0.80 (0.53-1.18) |  |
| 2019 | Taleban | Isfahan | Prospective cohort studies | ＞18 | 1,191 | 7,893 | Technicians measured | NA | Premenopause | WHR | 0.81-0.85 | NA | 1.13 (0.78-1.63) |  |
| 2019 | Taleban | Isfahan | Prospective cohort studies | ＞18 | 1,191 | 7,893 | Technicians measured | NA | Premenopause | WHR | ≥0.85 | NA | 1.03 (0.69-1.53) |  |
| 2019 | Taleban | Isfahan | Prospective cohort studies | ＞18 | 1,191 | 7,893 | Technicians measured | NA | Postmenopause | BMI | <18.5 | NA | 1.51 (0.81-2.30) |  |
| 2019 | Taleban | Isfahan | Prospective cohort studies | ＞18 | 1,191 | 7,893 | Technicians measured | NA | Postmenopause | BMI | 18-25 | NA | 1.00 |  |
| 2019 | Taleban | Isfahan | Prospective cohort studies | ＞18 | 1,191 | 7,893 | Technicians measured | NA | Postmenopause | BMI | 25-30 | NA | 0.90 (0.77-1.03) |  |
| 2019 | Taleban | Isfahan | Prospective cohort studies | ＞18 | 1,191 | 7,893 | Technicians measured | NA | Postmenopause | BMI | ≥30 | NA | 0.80 (0.68-0.94) |  |
| 2019 | Taleban | Isfahan | Prospective cohort studies | ＞18 | 1,191 | 7,893 | Technicians measured | NA | Postmenopause | WHR | <0.77 | NA | 1.00 |  |
| 2019 | Taleban | Isfahan | Prospective cohort studies | ＞18 | 1,191 | 7,893 | Technicians measured | NA | Postmenopause | WHR | 0.77-0.81 | NA | 1.06 (0.87-1.27) |  |
| 2019 | Taleban | Isfahan | Prospective cohort studies | ＞18 | 1,191 | 7,893 | Technicians measured | NA | Postmenopause | WHR | 0.81-0.85 | NA | 0.93 (0.77-1.13) |  |
| 2019 | Taleban | Isfahan | Prospective cohort studies | ＞18 | 1,191 | 7,893 | Technicians measured | NA | Postmenopause | WHR | ≥0.85 | NA | 1.26 (1.05-1.49) |  |
| 2021 | Pader | Canada | Prospective cohort studies | 35-74 | 609 | 2,436 | Self-report | NA | NA | BMI | < 25 | NA | 1.00 | Age at menarche, alcohol frequency in past 12 months, smoking status, income, education, total number of pregnancies, physical activity, fruit and vegetable consumption, and family history of BC. |
| 2021 | Pader | Canada | Prospective cohort studies | 35-74 | 609 | 2,436 | Self-report | NA | NA | BMI | 25–29.99 | NA | 0.80 (0.64-0.99) |  |
| 2021 | Pader | Canada | Prospective cohort studies | 35-74 | 609 | 2,436 | Self-report | NA | NA | BMI | ≥ 30 | NA | 0.71 (0.54-0.92) |  |
| 2021 | Pader | Canada | Prospective cohort studies | 35-74 | 609 | 2,436 | Self-report | NA | NA | WC | < 88 | NA | 1.00 |  |
| 2021 | Pader | Canada | Prospective cohort studies | 35-74 | 609 | 2,436 | Self-report | NA | NA | WC | ≥ 88 | NA | 1.40 (1.14-1.70) |  |
| 2021 | Pader | Canada | Prospective cohort studies | 35-74 | 609 | 2,436 | Self-report | NA | NA | WHR | < 0.83 | NA | 1.00 |  |
| 2021 | Pader | Canada | Prospective cohort studies | 35-74 | 609 | 2,436 | Self-report | NA | NA | WHR | ≥ 0.83 | NA | 1.30 (1.05-1.57) |  |
| 2021 | Park | Korean | Prospective cohort studies | ≥40 | 57,626 | 6,272,367 | Self reported | 6.20 | Premenopausal | BMI | < 18.5 | NA | 1.03 (0.95-1.11) | Age, income, smoking status, alcohol drinking, regular physical activity, parity, duration of breastfeeding, duration of oral contraceptive use, age at menarche, age at menopause, and duration of HRT. |
| 2021 | Park | Korean | Prospective cohort studies | ≥40 | 57,626 | 6,272,367 | Self reported | 6.20 | Premenopausal | BMI | 18.5–22.9 | NA | 1.00 |  |
| 2021 | Park | Korean | Prospective cohort studies | ≥40 | 57,626 | 6,272,367 | Self reported | 6.20 | Premenopausal | BMI | 23–24.9 | NA | 0.98 (0.95-1.02) |  |
| 2021 | Park | Korean | Prospective cohort studies | ≥40 | 57,626 | 6,272,367 | Self reported | 6.20 | Premenopausal | BMI | 25–29.9 | NA | 0.93 (0.89-0.97) |  |
| 2021 | Park | Korean | Prospective cohort studies | ≥40 | 57,626 | 6,272,367 | Self reported | 6.20 | Premenopausal | BMI | ≥ 30 | NA | 0.87 (0.80-0.95) |  |
| 2021 | Park | Korean | Prospective cohort studies | ≥40 | 57,626 | 6,272,367 | Self reported | 6.20 | Premenopausal | WC | < 65 | NA | 1.03 (0.97-1.09) |  |
| 2021 | Park | Korean | Prospective cohort studies | ≥40 | 57,626 | 6,272,367 | Self reported | 6.20 | Premenopausal | WC | 65–74.9 | NA | 1.00 |  |
| 2021 | Park | Korean | Prospective cohort studies | ≥40 | 57,626 | 6,272,367 | Self reported | 6.20 | Premenopausal | WC | 75–84.9 | NA | 0.98 (0.95-1.02) |  |
| 2021 | Park | Korean | Prospective cohort studies | ≥40 | 57,626 | 6,272,367 | Self reported | 6.20 | Premenopausal | WC | 85–94.9 | NA | 0.91 (0.86-0.96) |  |
| 2021 | Park | Korean | Prospective cohort studies | ≥40 | 57,626 | 6,272,367 | Self reported | 6.20 | Premenopausal | WC | ≥ 95 | NA | 0.90 (0.80-1.00) |  |
| 2021 | Park | Korean | Prospective cohort studies | ≥40 | 57,626 | 6,272,367 | Self reported | 6.20 | Postmenopause | BMI | < 18.5 | NA | 0.83 (0.76-0.90) |  |
| 2021 | Park | Korean | Prospective cohort studies | ≥40 | 57,626 | 6,272,367 | Self reported | 6.20 | Postmenopause | BMI | 18.5–22.9 | NA | 1.00 |  |
| 2021 | Park | Korean | Prospective cohort studies | ≥40 | 57,626 | 6,272,367 | Self reported | 6.20 | Postmenopause | BMI | 23–24.9 | NA | 1.11 (1.08-1.14) |  |
| 2021 | Park | Korean | Prospective cohort studies | ≥40 | 57,626 | 6,272,367 | Self reported | 6.20 | Postmenopause | BMI | 25–29.9 | NA | 1.27 (1.24-1.30) |  |
| 2021 | Park | Korean | Prospective cohort studies | ≥40 | 57,626 | 6,272,367 | Self reported | 6.20 | Postmenopause | BMI | ≥ 30 | NA | 1.52 (1.45-1.59) |  |
| 2021 | Park | Korean | Prospective cohort studies | ≥40 | 57,626 | 6,272,367 | Self reported | 6.20 | Postmenopause | WC | < 65 | NA | 0.93 (0.87-1.00) |  |
| 2021 | Park | Korean | Prospective cohort studies | ≥40 | 57,626 | 6,272,367 | Self reported | 6.20 | Postmenopause | WC | 65–74.9 | NA | 1.00 |  |
| 2021 | Park | Korean | Prospective cohort studies | ≥40 | 57,626 | 6,272,367 | Self reported | 6.20 | Postmenopause | WC | 75–84.9 | NA | 1.14 (1.11-1.17) |  |
| 2021 | Park | Korean | Prospective cohort studies | ≥40 | 57,626 | 6,272,367 | Self reported | 6.20 | Postmenopause | WC | 85–94.9 | NA | 1.31 (1.27-1.35) |  |
| 2021 | Park | Korean | Prospective cohort studies | ≥40 | 57,626 | 6,272,367 | Self reported | 6.20 | Postmenopause | WC | ≥ 95 | NA | 1.47 (1.40-1.55) |  |
| 2021 | Arthu | England | Prospective cohort studies | 40-70 | 1,051 | 149,928 | Technicians measured | 7.00 | Postmenopause | BMI | ≤ 21.4 | NA | 1.00 | Age at enrollment, education, age at menarche, age at first full-term birth and parity combined, HRT status, age at menopause, height, physical activity, alcohol intake, and smoking. |
| 2021 | Arthu | England | Prospective cohort studies | 40-70 | 1,051 | 149,928 | Technicians measured | 7.00 | Postmenopause | BMI | 21.5–22.6 | NA | 1.27 (1.04-1.55) |  |
| 2021 | Arthu | England | Prospective cohort studies | 40-70 | 1,051 | 149,928 | Technicians measured | 7.00 | Postmenopause | BMI | 22.7–23.5 | NA | 1.30 (1.06-1.59) |  |
| 2021 | Arthu | England | Prospective cohort studies | 40-70 | 1,051 | 149,928 | Technicians measured | 7.00 | Postmenopause | BMI | 23.6–24.2 | NA | 1.38 (1.13-1.69) |  |
| 2021 | Arthu | England | Prospective cohort studies | 40-70 | 1,051 | 149,928 | Technicians measured | 7.00 | Postmenopause | BMI | >24.2 | NA | 1.56 (1.28-1.90) |  |
| 2021 | Arthu | England | Prospective cohort studies | 40-70 | 1,051 | 149,928 | Technicians measured | 7.00 | Postmenopause | WC | ≤ 70 | NA | 1.00 |  |
| 2021 | Arthu | England | Prospective cohort studies | 40-70 | 1,051 | 149,928 | Technicians measured | 7.00 | Postmenopause | WC | 71–74 | NA | 1.04 (0.86-1.25) |  |
| 2021 | Arthu | England | Prospective cohort studies | 40-70 | 1,051 | 149,928 | Technicians measured | 7.00 | Postmenopause | WC | 75–77 | NA | 1.20 (0.99-1.46) |  |
| 2021 | Arthu | England | Prospective cohort studies | 40-70 | 1,051 | 149,928 | Technicians measured | 7.00 | Postmenopause | WC | 78–80.3 | NA | 1.20 (0.98-1.48) |  |
| 2021 | Arthu | England | Prospective cohort studies | 40-70 | 1,051 | 149,928 | Technicians measured | 7.00 | Postmenopause | WC | >80.3 | NA | 1.32 (1.09-1.60) |  |
| 2021 | Arthu | England | Prospective cohort studies | 40-70 | 1,051 | 149,928 | Technicians measured | 7.00 | Postmenopause | WHR | ≤ 0.74 | NA | 1.00 |  |
| 2021 | Arthu | England | Prospective cohort studies | 40-70 | 1,051 | 149,928 | Technicians measured | 7.00 | Postmenopause | WHR | 0.75–0.77 | NA | 1.08 (0.88-1.32) |  |
| 2021 | Arthu | England | Prospective cohort studies | 40-70 | 1,051 | 149,928 | Technicians measured | 7.00 | Postmenopause | WHR | 0.78–0.80 | NA | 1.15 (0.94-1.40) |  |
| 2021 | Arthu | England | Prospective cohort studies | 40-70 | 1,051 | 149,928 | Technicians measured | 7.00 | Postmenopause | WHR | 0.81–0.83 | NA | 1.12 (0.92-1.36) |  |
| 2021 | Arthu | England | Prospective cohort studies | 40-70 | 1,051 | 149,928 | Technicians measured | 7.00 | Postmenopause | WHR | >0.83 | NA | 1.21 (0.99-1.47) |  |
| 2021 | Houghton | USA | Prospective cohort studies | 25-55 | 6,129 | 238,129 | Self-report | 24–30 | Premenopausal | WC | Q1 | NA | 1.00 | Age at menarche, height, parity/age at first birth, family history of BC benign breast disease diagnosis， alcohol intake, physical activity, and smoking. |
| 2021 | Houghton | USA | Prospective cohort studies | 25-55 | 6,129 | 238,129 | Self-report | 24–30 | Premenopausal | WC | Q2 | NA | 0.98 (0.82-1.19) |  |
| 2021 | Houghton | USA | Prospective cohort studies | 25-55 | 6,129 | 238,129 | Self-report | 24–30 | Premenopausal | WC | Q3 | NA | 1.11 (0.91-1.36) |  |
| 2021 | Houghton | USA | Prospective cohort studies | 25-55 | 6,129 | 238,129 | Self-report | 24–30 | Premenopausal | WC | Q4 | NA | 1.34 (1.09-1.64) |  |
| 2021 | Houghton | USA | Prospective cohort studies | 25-55 | 6,129 | 238,129 | Self-report | 24–30 | Premenopausal | WC | Q5 | NA | 1.12 (0.86-1.45) |  |
| 2021 | Houghton | USA | Prospective cohort studies | 25-55 | 6,129 | 238,129 | Self-report | 24–30 | Premenopausal | HC | Q1 | NA | 1.00 |  |
| 2021 | Houghton | USA | Prospective cohort studies | 25-55 | 6,129 | 238,129 | Self-report | 24–30 | Premenopausal | HC | Q2 | NA | 1.09 (0.91-1.30) |  |
| 2021 | Houghton | USA | Prospective cohort studies | 25-55 | 6,129 | 238,129 | Self-report | 24–30 | Premenopausal | HC | Q3 | NA | 1.03 (0.83-1.27) |  |
| 2021 | Houghton | USA | Prospective cohort studies | 25-55 | 6,129 | 238,129 | Self-report | 24–30 | Premenopausal | HC | Q4 | NA | 1.11 (0.89-1.37) |  |
| 2021 | Houghton | USA | Prospective cohort studies | 25-55 | 6,129 | 238,129 | Self-report | 24–30 | Premenopausal | HC | Q5 | NA | 1.03 (0.78-1.36) |  |
| 2021 | Houghton | USA | Prospective cohort studies | 25-55 | 6,129 | 238,129 | Self-report | 24–30 | Premenopausal | WHR | Q1 | NA | 1.00 |  |
| 2021 | Houghton | USA | Prospective cohort studies | 25-55 | 6,129 | 238,129 | Self-report | 24–30 | Premenopausal | WHR | Q2 | NA | 0.97 (0.80-1.18) |  |
| 2021 | Houghton | USA | Prospective cohort studies | 25-55 | 6,129 | 238,129 | Self-report | 24–30 | Premenopausal | WHR | Q3 | NA | 1.13 (0.94-1.37) |  |
| 2021 | Houghton | USA | Prospective cohort studies | 25-55 | 6,129 | 238,129 | Self-report | 24–30 | Premenopausal | WHR | Q4 | NA | 1.13 (0.93-1.37) |  |
| 2021 | Houghton | USA | Prospective cohort studies | 25-55 | 6,129 | 238,129 | Self-report | 24–30 | Premenopausal | WHR | Q5 | NA | 1.27 (1.04-1.54) |  |
| 2021 | Houghton | USA | Prospective cohort studies | 25-55 | 6,129 | 238,129 | Self-report | 24–30 | Premenopausal | BMI | Q1 | NA | 1.00 |  |
| 2021 | Houghton | USA | Prospective cohort studies | 25-55 | 6,129 | 238,129 | Self-report | 24–30 | Premenopausal | BMI | Q2 | NA | 0.90 (0.75-1.08) |  |
| 2021 | Houghton | USA | Prospective cohort studies | 25-55 | 6,129 | 238,129 | Self-report | 24–30 | Premenopausal | BMI | Q3 | NA | 0.95 (0.80-1.14) |  |
| 2021 | Houghton | USA | Prospective cohort studies | 25-55 | 6,129 | 238,129 | Self-report | 24–30 | Premenopausal | BMI | Q4 | NA | 0.88 (0.73-1.06) |  |
| 2021 | Houghton | USA | Prospective cohort studies | 25-55 | 6,129 | 238,129 | Self-report | 24–30 | Premenopausal | BMI | Q5 | NA | 0.75 (0.61-0.91) |  |
| 2021 | Houghton | USA | Prospective cohort studies | 25-55 | 6,129 | 238,129 | Self-report | 24–30 | Postmenopause | WC | Q1 | NA | 1.00 |  |
| 2021 | Houghton | USA | Prospective cohort studies | 25-55 | 6,129 | 238,129 | Self-report | 24–30 | Postmenopause | WC | Q2 | NA | 1.27 (1.07-1.51) |  |
| 2021 | Houghton | USA | Prospective cohort studies | 25-55 | 6,129 | 238,129 | Self-report | 24–30 | Postmenopause | WC | Q3 | NA | 1.16 (0.98-1.37) |  |
| 2021 | Houghton | USA | Prospective cohort studies | 25-55 | 6,129 | 238,129 | Self-report | 24–30 | Postmenopause | WC | Q4 | NA | 1.28 (1.09-1.51) |  |
| 2021 | Houghton | USA | Prospective cohort studies | 25-55 | 6,129 | 238,129 | Self-report | 24–30 | Postmenopause | WC | Q5 | NA | 1.38 (1.15-1.64) |  |
| 2021 | Houghton | USA | Prospective cohort studies | 25-55 | 6,129 | 238,129 | Self-report | 24–30 | Postmenopause | HC | Q1 | NA | 1.00 |  |
| 2021 | Houghton | USA | Prospective cohort studies | 25-55 | 6,129 | 238,129 | Self-report | 24–30 | Postmenopause | HC | Q2 | NA | 1.14 (0.99-1.31) |  |
| 2021 | Houghton | USA | Prospective cohort studies | 25-55 | 6,129 | 238,129 | Self-report | 24–30 | Postmenopause | HC | Q3 | NA | 1.04 (0.89-1.20) |  |
| 2021 | Houghton | USA | Prospective cohort studies | 25-55 | 6,129 | 238,129 | Self-report | 24–30 | Postmenopause | HC | Q4 | NA | 1.05 (0.91-1.21) |  |
| 2021 | Houghton | USA | Prospective cohort studies | 25-55 | 6,129 | 238,129 | Self-report | 24–30 | Postmenopause | HC | Q5 | NA | 1.10 (0.93-1.30) |  |
| 2021 | Houghton | USA | Prospective cohort studies | 25-55 | 6,129 | 238,129 | Self-report | 24–30 | Postmenopause | WHR | Q1 | NA | 1.00 |  |
| 2021 | Houghton | USA | Prospective cohort studies | 25-55 | 6,129 | 238,129 | Self-report | 24–30 | Postmenopause | WHR | Q2 | NA | 1.02 (0.87-1.19) |  |
| 2021 | Houghton | USA | Prospective cohort studies | 25-55 | 6,129 | 238,129 | Self-report | 24–30 | Postmenopause | WHR | Q3 | NA | 0.99 (0.85-1.15) |  |
| 2021 | Houghton | USA | Prospective cohort studies | 25-55 | 6,129 | 238,129 | Self-report | 24–30 | Postmenopause | WHR | Q4 | NA | 1.11 (0.97-1.29) |  |
| 2021 | Houghton | USA | Prospective cohort studies | 25-55 | 6,129 | 238,129 | Self-report | 24–30 | Postmenopause | WHR | Q5 | NA | 1.15 (1.00-1.32) |  |
| 2021 | Houghton | USA | Prospective cohort studies | 25-55 | 6,129 | 238,129 | Self-report | 24–30 | Postmenopause | BMI | Q1 | NA | 1.00 |  |
| 2021 | Houghton | USA | Prospective cohort studies | 25-55 | 6,129 | 238,129 | Self-report | 24–30 | Postmenopause | BMI | Q2 | NA | 1.06 (0.91-1.22) |  |
| 2021 | Houghton | USA | Prospective cohort studies | 25-55 | 6,129 | 238,129 | Self-report | 24–30 | Postmenopause | BMI | Q3 | NA | 1.11 (0.96-1.27) |  |
| 2021 | Houghton | USA | Prospective cohort studies | 25-55 | 6,129 | 238,129 | Self-report | 24–30 | Postmenopause | BMI | Q4 | NA | 1.17 (1.02-1.34) |  |
| 2021 | Houghton | USA | Prospective cohort studies | 25-55 | 6,129 | 238,129 | Self-report | 24–30 | Postmenopause | BMI | Q5 | NA | 1.44 (1.26-1.65) |  |
| 2021 | Houghton | USA | Prospective cohort studies | 25-55 | 6,129 | 238,129 | Self-report | 24–30 | Pre-Pre | WC | NA | ER+PR+ | 1.13 (0.87-1.47) |  |
| 2021 | Houghton | USA | Prospective cohort studies | 25-55 | 6,129 | 238,129 | Self-report | 24–30 | Pre-Pre | WC | NA | ER+PR- | 0.99 (0.83-1.18) |  |
| 2021 | Houghton | USA | Prospective cohort studies | 25-55 | 6,129 | 238,129 | Self-report | 24–30 | Pre-Pre | WC | NA | ER-PR- | 1.27 (1.09-1.46) |  |
| 2021 | Houghton | USA | Prospective cohort studies | 25-55 | 6,129 | 238,129 | Self-report | 24–30 | Pre-Pre | WC | NA | Luminal A | 0.46 (0.22-0.96) |  |
| 2021 | Houghton | USA | Prospective cohort studies | 25-55 | 6,129 | 238,129 | Self-report | 24–30 | Pre-Pre | WC | NA | Luminal B | 0.85 (0.61-1.18) |  |
| 2021 | Houghton | USA | Prospective cohort studies | 25-55 | 6,129 | 238,129 | Self-report | 24–30 | Pre-Pre | WC | NA | HER2+ | 0.81 (0.62-1.06) |  |
| 2021 | Houghton | USA | Prospective cohort studies | 25-55 | 6,129 | 238,129 | Self-report | 24–30 | Pre-Pre | WC | NA | Basal-like | 1.99 (1.35-2.94) |  |
| 2021 | Houghton | USA | Prospective cohort studies | 25-55 | 6,129 | 238,129 | Self-report | 24–30 | Pre-Pre | HC | NA | ER+PR+ | 1.00 (0.75-1.34) |  |
| 2021 | Houghton | USA | Prospective cohort studies | 25-55 | 6,129 | 238,129 | Self-report | 24–30 | Pre-Pre | HC | NA | ER+PR- | 0.88 (0.68-1.13) |  |
| 2021 | Houghton | USA | Prospective cohort studies | 25-55 | 6,129 | 238,129 | Self-report | 24–30 | Pre-Pre | HC | NA | ER-PR- | 0.98 (0.71-1.35) |  |
| 2021 | Houghton | USA | Prospective cohort studies | 25-55 | 6,129 | 238,129 | Self-report | 24–30 | Pre-Pre | HC | NA | Luminal A | 0.89 (0.73-1.09) |  |
| 2021 | Houghton | USA | Prospective cohort studies | 25-55 | 6,129 | 238,129 | Self-report | 24–30 | Pre-Pre | HC | NA | Luminal B | 1.14 (0.95-1.36) |  |
| 2021 | Houghton | USA | Prospective cohort studies | 25-55 | 6,129 | 238,129 | Self-report | 24–30 | Pre-Pre | HC | NA | HER2+ | 1.12 (0.75-1.69) |  |
| 2021 | Houghton | USA | Prospective cohort studies | 25-55 | 6,129 | 238,129 | Self-report | 24–30 | Pre-Pre | HC | NA | Basal-like | 1.09 (0.84-1.42) |  |
| 2021 | Houghton | USA | Prospective cohort studies | 25-55 | 6,129 | 238,129 | Self-report | 24–30 | Pre-Pre | WHR | NA | ER+PR+ | 1.24 (0.98-1.58) |  |
| 2021 | Houghton | USA | Prospective cohort studies | 25-55 | 6,129 | 238,129 | Self-report | 24–30 | Pre-Pre | WHR | NA | ER+PR- | 1.95 (0.91-4.21) |  |
| 2021 | Houghton | USA | Prospective cohort studies | 25-55 | 6,129 | 238,129 | Self-report | 24–30 | Pre-Pre | WHR | NA | ER-PR- | 0.80 (0.48-1.33) |  |
| 2021 | Houghton | USA | Prospective cohort studies | 25-55 | 6,129 | 238,129 | Self-report | 24–30 | Pre-Pre | WHR | NA | Luminal A | 0.79 (0.48-1.27) |  |
| 2021 | Houghton | USA | Prospective cohort studies | 25-55 | 6,129 | 238,129 | Self-report | 24–30 | Pre-Pre | WHR | NA | Luminal B | 3.58 (1.94-6.63) |  |
| 2021 | Houghton | USA | Prospective cohort studies | 25-55 | 6,129 | 238,129 | Self-report | 24–30 | Pre-Pre | WHR | NA | HER2+ | 2.05 (0.88-4.74) |  |
| 2021 | Houghton | USA | Prospective cohort studies | 25-55 | 6,129 | 238,129 | Self-report | 24–30 | Pre-Pre | WHR | NA | Basal-like | 0.90 (0.53-1.53) |  |
| 2021 | Houghton | USA | Prospective cohort studies | 25-55 | 6,129 | 238,129 | Self-report | 24–30 | Pre-Post | WC | NA | ER+PR+ | 0.97 (0.73-1.29) |  |
| 2021 | Houghton | USA | Prospective cohort studies | 25-55 | 6,129 | 238,129 | Self-report | 24–30 | Pre-Post | WC | NA | ER+PR- | 1.07 (0.88-1.30) |  |
| 2021 | Houghton | USA | Prospective cohort studies | 25-55 | 6,129 | 238,129 | Self-report | 24–30 | Pre-Post | WC | NA | ER-PR- | 1.11 (0.94-1.31) |  |
| 2021 | Houghton | USA | Prospective cohort studies | 25-55 | 6,129 | 238,129 | Self-report | 24–30 | Pre-Post | WC | NA | Luminal A | 0.65 (0.33-1.25) |  |
| 2021 | Houghton | USA | Prospective cohort studies | 25-55 | 6,129 | 238,129 | Self-report | 24–30 | Pre-Post | WC | NA | Luminal B | 0.79 (0.56-1.13) |  |
| 2021 | Houghton | USA | Prospective cohort studies | 25-55 | 6,129 | 238,129 | Self-report | 24–30 | Pre-Post | WC | NA | HER2+ | 0.82 (0.63-1.08) |  |
| 2021 | Houghton | USA | Prospective cohort studies | 25-55 | 6,129 | 238,129 | Self-report | 24–30 | Pre-Post | WC | NA | Basal-like | 1.50 (1.00-2.26) |  |
| 2021 | Houghton | USA | Prospective cohort studies | 25-55 | 6,129 | 238,129 | Self-report | 24–30 | Pre-Post | HC | NA | ER+PR+ | 1.02 (0.75-1.39) |  |
| 2021 | Houghton | USA | Prospective cohort studies | 25-55 | 6,129 | 238,129 | Self-report | 24–30 | Pre-Post | HC | NA | ER+PR- | 0.80 (0.62-1.04) |  |
| 2021 | Houghton | USA | Prospective cohort studies | 25-55 | 6,129 | 238,129 | Self-report | 24–30 | Pre-Post | HC | NA | ER-PR- | 0.76 (0.54-1.08) |  |
| 2021 | Houghton | USA | Prospective cohort studies | 25-55 | 6,129 | 238,129 | Self-report | 24–30 | Pre-Post | HC | NA | Luminal A | 0.98 (0.78-1.22) |  |
| 2021 | Houghton | USA | Prospective cohort studies | 25-55 | 6,129 | 238,129 | Self-report | 24–30 | Pre-Post | HC | NA | Luminal B | 1.01 (0.83-1.23) |  |
| 2021 | Houghton | USA | Prospective cohort studies | 25-55 | 6,129 | 238,129 | Self-report | 24–30 | Pre-Post | HC | NA | HER2+ | 0.95 (0.62-1.45) |  |
| 2021 | Houghton | USA | Prospective cohort studies | 25-55 | 6,129 | 238,129 | Self-report | 24–30 | Pre-Post | HC | NA | Basal-like | 1.27 (0.96-1.68) |  |
| 2021 | Houghton | USA | Prospective cohort studies | 25-55 | 6,129 | 238,129 | Self-report | 24–30 | Pre-Post | WHR | NA | ER+PR+ | 0.96 (0.74-1.25) |  |
| 2021 | Houghton | USA | Prospective cohort studies | 25-55 | 6,129 | 238,129 | Self-report | 24–30 | Pre-Post | WHR | NA | ER+PR- | 1.39 (0.63-3.08) |  |
| 2021 | Houghton | USA | Prospective cohort studies | 25-55 | 6,129 | 238,129 | Self-report | 24–30 | Pre-Post | WHR | NA | ER-PR- | 0.94 (0.55-1.60) |  |
| 2021 | Houghton | USA | Prospective cohort studies | 25-55 | 6,129 | 238,129 | Self-report | 24–30 | Pre-Post | WHR | NA | Luminal A | 0.75 (0.45-1.23) |  |
| 2021 | Houghton | USA | Prospective cohort studies | 25-55 | 6,129 | 238,129 | Self-report | 24–30 | Pre-Post | WHR | NA | Luminal B | 1.83 (0.94-3.54) |  |
| 2021 | Houghton | USA | Prospective cohort studies | 25-55 | 6,129 | 238,129 | Self-report | 24–30 | Pre-Post | WHR | NA | HER2+ | 1.54 (0.63-3.77) |  |
| 2021 | Houghton | USA | Prospective cohort studies | 25-55 | 6,129 | 238,129 | Self-report | 24–30 | Pre-Post | WHR | NA | Basal-like | 0.69 (0.40-1.18) |  |
| 2021 | Houghton | USA | Prospective cohort studies | 25-55 | 6,129 | 238,129 | Self-report | 24–30 | Post-Post | WC | NA | ER+PR+ | 1.11 (0.93-1.32) |  |
| 2021 | Houghton | USA | Prospective cohort studies | 25-55 | 6,129 | 238,129 | Self-report | 24–30 | Post-Post | WC | NA | ER+PR- | 0.95 (0.83-1.08) |  |
| 2021 | Houghton | USA | Prospective cohort studies | 25-55 | 6,129 | 238,129 | Self-report | 24–30 | Post-Post | WC | NA | ER-PR- | 1.13 (1.03-1.24) |  |
| 2021 | Houghton | USA | Prospective cohort studies | 25-55 | 6,129 | 238,129 | Self-report | 24–30 | Post-Post | WC | NA | Luminal A | 0.48 (0.25-0.92) |  |
| 2021 | Houghton | USA | Prospective cohort studies | 25-55 | 6,129 | 238,129 | Self-report | 24–30 | Post-Post | WC | NA | Luminal B | 1.01 (0.76-1.35) |  |
| 2021 | Houghton | USA | Prospective cohort studies | 25-55 | 6,129 | 238,129 | Self-report | 24–30 | Post-Post | WC | NA | HER2+ | 0.86 (0.69-1.07) |  |
| 2021 | Houghton | USA | Prospective cohort studies | 25-55 | 6,129 | 238,129 | Self-report | 24–30 | Post-Post | WC | NA | Basal-like | 1.50 (1.13-2.01) |  |
| 2021 | Houghton | USA | Prospective cohort studies | 25-55 | 6,129 | 238,129 | Self-report | 24–30 | Post-Post | HC | NA | ER+PR+ | 1.05 (0.81-1.34) |  |
| 2021 | Houghton | USA | Prospective cohort studies | 25-55 | 6,129 | 238,129 | Self-report | 24–30 | Post-Post | HC | NA | ER+PR- | 0.94 (0.77-1.15) |  |
| 2021 | Houghton | USA | Prospective cohort studies | 25-55 | 6,129 | 238,129 | Self-report | 24–30 | Post-Post | HC | NA | ER-PR- | 1.08 (0.86-1.35) |  |
| 2021 | Houghton | USA | Prospective cohort studies | 25-55 | 6,129 | 238,129 | Self-report | 24–30 | Post-Post | HC | NA | Luminal A | 0.92 (0.79-1.07) |  |
| 2021 | Houghton | USA | Prospective cohort studies | 25-55 | 6,129 | 238,129 | Self-report | 24–30 | Post-Post | HC | NA | Luminal B | 1.05 (0.93-1.18) |  |
| 2021 | Houghton | USA | Prospective cohort studies | 25-55 | 6,129 | 238,129 | Self-report | 24–30 | Post-Post | HC | NA | HER2+ | 1.05 (0.76-1.44) |  |
| 2021 | Houghton | USA | Prospective cohort studies | 25-55 | 6,129 | 238,129 | Self-report | 24–30 | Post-Post | HC | NA | Basal-like | 0.93 (0.74-1.17) |  |
| 2021 | Houghton | USA | Prospective cohort studies | 25-55 | 6,129 | 238,129 | Self-report | 24–30 | Post-Post | WHR | NA | ER+PR+ | 1.21 (1.02-1.43) |  |
| 2021 | Houghton | USA | Prospective cohort studies | 25-55 | 6,129 | 238,129 | Self-report | 24–30 | Post-Post | WHR | NA | ER+PR- | 1.33 (0.70-2.53) |  |
| 2021 | Houghton | USA | Prospective cohort studies | 25-55 | 6,129 | 238,129 | Self-report | 24–30 | Post-Post | WHR | NA | ER-PR- | 0.85 (0.53-1.38) |  |
| 2021 | Houghton | USA | Prospective cohort studies | 25-55 | 6,129 | 238,129 | Self-report | 24–30 | Post-Post | WHR | NA | Luminal A | 0.81 (0.54-1.22) |  |
| 2021 | Houghton | USA | Prospective cohort studies | 25-55 | 6,129 | 238,129 | Self-report | 24–30 | Post-Post | WHR | NA | Luminal B | 2.14 (1.43-3.20) |  |
| 2021 | Houghton | USA | Prospective cohort studies | 25-55 | 6,129 | 238,129 | Self-report | 24–30 | Post-Post | WHR | NA | HER2+ | 1.65 (0.90-2.99) |  |
| 2021 | Houghton | USA | Prospective cohort studies | 25-55 | 6,129 | 238,129 | Self-report | 24–30 | Post-Post | WHR | NA | Basal-like | 1.08 (0.69-1.68) |  |
| 2022 | Lofterød | Oslo | Prospective cohort studies | 15–75 | 557 | 13,802 | Technicians measured | 7.70 | NA | BMI | ≤24.6 | ER+/PR+ | 1.00 | Age and year at diagnosis, BMI when studying differences in BC subtype by ethnicity, whereas age and year at diagnosis, BMI, subtype of BC, comorbidity at the time of BC diagnosis, pre-diagnostic physical activity, and level of education. |
| 2022 | Lofterød | Oslo | Prospective cohort studies | 15–75 | 557 | 13,802 | Technicians measured | 7.70 | NA | BMI | >24.6 | ER+/PR+ | 1.11 (0.68-1.83) |  |
| 2022 | Lofterød | Oslo | Prospective cohort studies | 15–75 | 557 | 13,802 | Technicians measured | 7.70 | NA | WHR | ≤0.79 | ER+/PR+ | 1.00 |  |
| 2022 | Lofterød | Oslo | Prospective cohort studies | 15–75 | 557 | 13,802 | Technicians measured | 7.70 | NA | WHR | >0.79 | ER+/PR+ | 1.85 (1.05-2.15) |  |
| 2022 | Lofterød | Oslo | Prospective cohort studies | 15–75 | 557 | 13,802 | Technicians measured | 7.70 | NA | BMI | ≤24.6 | HER2+ | 1.00 |  |
| 2022 | Lofterød | Oslo | Prospective cohort studies | 15–75 | 557 | 13,802 | Technicians measured | 7.70 | NA | BMI | >24.6 | HER2+ | 3.39 (0.82-14.00) |  |
| 2022 | Lofterød | Oslo | Prospective cohort studies | 15–75 | 557 | 13,802 | Technicians measured | 7.70 | NA | WHR | ≤0.79 | HER2+ | 1.00 |  |
| 2022 | Lofterød | Oslo | Prospective cohort studies | 15–75 | 557 | 13,802 | Technicians measured | 7.70 | NA | WHR | >0.79 | HER2+ | 0.56 (0.11-2.95) |  |
| 2022 | Lofterød | Oslo | Prospective cohort studies | 15–75 | 557 | 13,802 | Technicians measured | 7.70 | NA | BMI | ≤24.6 | Triple-negative | 1.00 |  |
| 2022 | Lofterød | Oslo | Prospective cohort studies | 15–75 | 557 | 13,802 | Technicians measured | 7.70 | NA | BMI | >24.6 | Triple-negative | 0.84 (0.31-2.25) |  |
| 2022 | Lofterød | Oslo | Prospective cohort studies | 15–75 | 557 | 13,802 | Technicians measured | 7.70 | NA | WHR | ≤0.79 | Triple-negative | 1.00 |  |
| 2022 | Lofterød | Oslo | Prospective cohort studies | 15–75 | 557 | 13,802 | Technicians measured | 7.70 | NA | WHR | >0.79 | Triple-negative | 1.10 (0.34-3.52) |  |
| 2022 | Lofterød | Oslo | Prospective cohort studies | 15–75 | 557 | 13,802 | Technicians measured | 7.70 | NA | BMI | ≤24.6 | NA | 1.00 |  |
| 2022 | Lofterød | Oslo | Prospective cohort studies | 15–75 | 557 | 13,802 | Technicians measured | 7.70 | NA | BMI | >24.6 | NA | 1.15 (0.75-1.76) |  |
| 2022 | Lofterød | Oslo | Prospective cohort studies | 15–75 | 557 | 13,802 | Technicians measured | 7.70 | NA | WHR | ≤0.79 | NA | 1.00 |  |
| 2022 | Lofterød | Oslo | Prospective cohort studies | 15–75 | 557 | 13,802 | Technicians measured | 7.70 | NA | WHR | >0.79 | NA | 1.46 (0.90-2.36) |  |

Abbreviations: RR, Relative risk; 95% CI, 95% confidence interval; BMI: body mass index; WC: waist circumference; HC: hip circumference; WHR: waist-hip-ratio; HRT: hormone replacement therapy; NA, not available; ER:estrogen receptor; PR: progesterone receptor; BC: breast cancer; ER+ : estrogen receptor-positive; ER-:Triple-: estrogen receptor-negative ; Triple-negative: ER−/PR−/HER2−; No.: number.
