# Supplementary material for: The relationship between central obesity and risk of breast cancer: a dose–response meta-analysis of 7,989,315 women
Source: Front Nutr. 2023 Nov 9;10:1236393. doi: 10.3389/fnut.2023.1236393 (PMC10665573; doi:10.3389/fnut.2023.1236393)
Supplement: Supplementary file 5 [file Table_5.DOCX]

**Supplementary Table 5. The quality of included case-cohort studies assessed by the Newcastle Ottawa Scale（n=26）**

|  | **Selection** | | | | **Comparability** | **Outcome** | | | **Total stars** |
| --- | --- | --- | --- | --- | --- | --- | --- | --- | --- |
| **Study** | **Case definition** | **Representativeness** | **Control selection** | **Control definition** | **Comparability of cohorts on the basis of the design or analysis** | **Ascertainment of exposure** | **Same method of ascertainment for cases and controls** | **Non-Response rate** |  |
| **Petrek，1993** | 1 | 0 | 1 | 1 | 2 | 0 | 1 | 1 | 7 |
| **Männistö，1996** | 1 | 0 | 1 | 1 | 2 | 0 | 1 | 0 | 6 |
| **Franceschi，1996** | 1 | 1 | 1 | 1 | 2 | 1 | 1 | 0 | 8 |
| **Swanson，1996** | 1 | 1 | 1 | 0 | 2 | 1 | 1 | 1 | 8 |
| **Ng，1997** | 1 | 0 | 0 | 1 | 2 | 1 | 1 | 0 | 6 |
| **Shu，2001** | 1 | 1 | 1 | 1 | 2 | 1 | 1 | 1 | 9 |
| **Friedenreich，2002** | 1 | 1 | 1 | 1 | 2 | 1 | 1 | 0 | 8 |
| **Adebamowo，2003** | 1 | 0 | 0 | 1 | 2 | 1 | 1 | 0 | 6 |
| **Okobia，2006** | 1 | 0 | 1 | 1 | 2 | 1 | 1 | 0 | 7 |
| **Okobia，2006** | 1 | 0 | 1 | 1 | 2 | 1 | 1 | 0 | 7 |
| **Mathew，2008** | 1 | 1 | 1 | 1 | 2 | 1 | 1 | 1 | 9 |
| **Shin，2009** | 1 | 1 | 1 | 1 | 2 | 1 | 1 | 1 | 9 |
| **John，2011** | 1 | 1 | 1 | 1 | 2 | 1 | 1 | 1 | 9 |
| **Ogundiran，2011** | 1 | 0 | 1 | 1 | 2 | 1 | 1 | 1 | 8 |
| **Amadou，2014** | 1 | 1 | 1 | 1 | 2 | 1 | 1 | 1 | 9 |
| **Nagrani，2016** | 1 | 0 | 1 | 1 | 2 | 1 | 1 | 0 | 7 |
| **Pacholczak，2016** | 1 | 1 | 0 | 1 | 2 | 1 | 1 | 0 | 7 |
| **Wang，2017** | 1 | 1 | 1 | 1 | 1 | 1 | 1 | 0 | 7 |
| **Leon Guerrero,2017** | 1 | 1 | 1 | 1 | 2 | 1 | 1 | 0 | 8 |
| **Godinho-Mota，2018** | 1 | 0 | 1 | 1 | 0 | 1 | 1 | 1 | 6 |
| **Kops，2018** | 1 | 1 | 1 | 1 | 2 | 1 | 1 | 1 | 9 |
| **Xiang，2019** | 1 | 0 | 1 | 1 | 2 | 1 | 1 | 0 | 7 |
| **His，2020** | 1 | 1 | 1 | 0 | 2 | 1 | 1 | 0 | 7 |
| **Khalis，2020** | 1 | 1 | 0 | 1 | 2 | 1 | 1 | 0 | 7 |
| **Benefield，2021** | 1 | 1 | 1 | 1 | 2 | 0 | 1 | 0 | 7 |
| **Ramírez-Marrero，2022** | 1 | 0 | 0 | 1 | 2 | 1 | 1 | 0 | 6 |
