# Supplementary material for: The relationship between central obesity and risk of breast cancer: a dose–response meta-analysis of 7,989,315 women
Source: Front Nutr. 2023 Nov 9;10:1236393. doi: 10.3389/fnut.2023.1236393 (PMC10665573; doi:10.3389/fnut.2023.1236393)
Supplement: Supplementary file 6 [file Table_6.DOCX]

**Supplementary Table 6. The quality of included prospective studies assessed by the Newcastle Ottawa Scale（n=31）**

|  | **Selection** | | | | **Comparability** | **Outcome** | | | **Total stars** |
| --- | --- | --- | --- | --- | --- | --- | --- | --- | --- |
| **Study** | **Representativeness**  **of exposed cohort** | **Selection of the non-exposed cohort** | **Ascertainment of exposure** | **Demonstration that outcome of interest was not present at start study** | **Comparability of cohorts on the basis of the design or analysis** | **Assessment of outcome** | **Was follow-up enough for outcomes to occur** | **Adequacy of follow up of cohorts** |  |
| **Folsom, 1990** | 1 | 1 | 0 | 1 | 2 | 1 | 0 | 0 | 6 |
| **Schapira，1990** | 1 | 0 | 1 | 1 | 2 | 1 | 0 | 1 | 7 |
| **Kaaks, 1998** | 0 | 1 | 1 | 0 | 2 | 1 | 1 | 0 | 6 |
| **Sonnenschein, 1999** | 0 | 1 | 1 | 1 | 2 | 1 | 1 | 0 | 7 |
| **Huang, 1999** | 0 | 1 | 1 | 1 | 2 | 1 | 1 | 1 | 8 |
| **Muti，2000** | 1 | 1 | 1 | 1 | 2 | 1 | 1 | 1 | 9 |
| **Sellers, 2002** | 1 | 1 | 1 | 1 | 1 | 1 | 1 | 0 | 7 |
| **Lahmann, 2004** | 1 | 1 | 1 | 1 | 2 | 1 | 0 | 0 | 7 |
| **Macinnis, 2004** | 1 | 1 | 1 | 1 | 2 | 1 | 1 | 1 | 9 |
| **Krebs, 2006** | 0 | 1 | 1 | 1 | 2 | 1 | 1 | 1 | 8 |
| **Tehard, 2006** | 0 | 1 | 0 | 1 | 2 | 1 | 0 | 1 | 6 |
| **Wu, 2006** | 1 | 1 | 1 | 1 | 2 | 1 | 1 | 1 | 9 |
| **Palmer, 2007** | 0 | 1 | 1 | 0 | 2 | 1 | 1 | 1 | 7 |
| **Harris, 2011** | 0 | 1 | 0 | 1 | 2 | 0 | 1 | 0 | 5 |
| **Phipps, 2011** | 0 | 1 | 1 | 1 | 2 | 1 | 1 | 1 | 8 |
| **Canchola, 2012** | 0 | 1 | 1 | 1 | 2 | 1 | 1 | 0 | 7 |
| **Fagherazzi, 2012** | 0 | 1 | 0 | 0 | 2 | 1 | 1 | 1 | 6 |
| **Fagherazzi, 2013** | 0 | 1 | 0 | 1 | 2 | 1 | 1 | 1 | 7 |
| **Catsburg，2014** | 1 | 1 | 1 | 1 | 2 | 0 | 1 | 0 | 7 |
| **Gaudet, 2014** | 1 | 1 | 0 | 1 | 2 | 1 | 1 | 1 | 8 |
| **White, 2015** | 0 | 1 | 1 | 1 | 2 | 1 | 1 | 1 | 8 |
| **Harding, 2015** | 1 | 1 | 1 | 1 | 2 | 1 | 1 | 0 | 8 |
| **Bellocco, 2016** | 1 | 1 | 0 | 1 | 2 | 1 | 1 | 1 | 8 |
| **Liu, 2016** | 0 | 1 | 1 | 0 | 2 | 1 | 1 | 1 | 7 |
| **Al Ajmi, 2018** | 1 | 1 | 1 | 0 | 2 | 1 | 1 | 0 | 7 |
| **Taleban，2019** | 1 | 1 | 1 | 1 | 2 | 1 | 0 | 1 | 8 |
| **Pader，2021** | 1 | 1 | 0 | 1 | 2 | 1 | 1 | 1 | 8 |
| **Park, 2021** | 1 | 1 | 0 | 1 | 2 | 1 | 1 | 0 | 7 |
| **Arthur, 2021** | 1 | 1 | 1 | 1 | 2 | 1 | 0 | 1 | 8 |
| **Houghton, 2021** | 0 | 1 | 0 | 1 | 2 | 1 | 1 | 1 | 7 |
| **Lofterød, 2022** | 1 | 1 | 1 | 1 | 2 | 1 | 1 | 0 | 8 |
