# Supplementary figures and images for: The relationship between central obesity and risk of breast cancer: a dose–response meta-analysis of 7,989,315 women
Source: Front Nutr. 2023 Nov 9;10:1236393. doi: 10.3389/fnut.2023.1236393 (PMC10665573; doi:10.3389/fnut.2023.1236393)

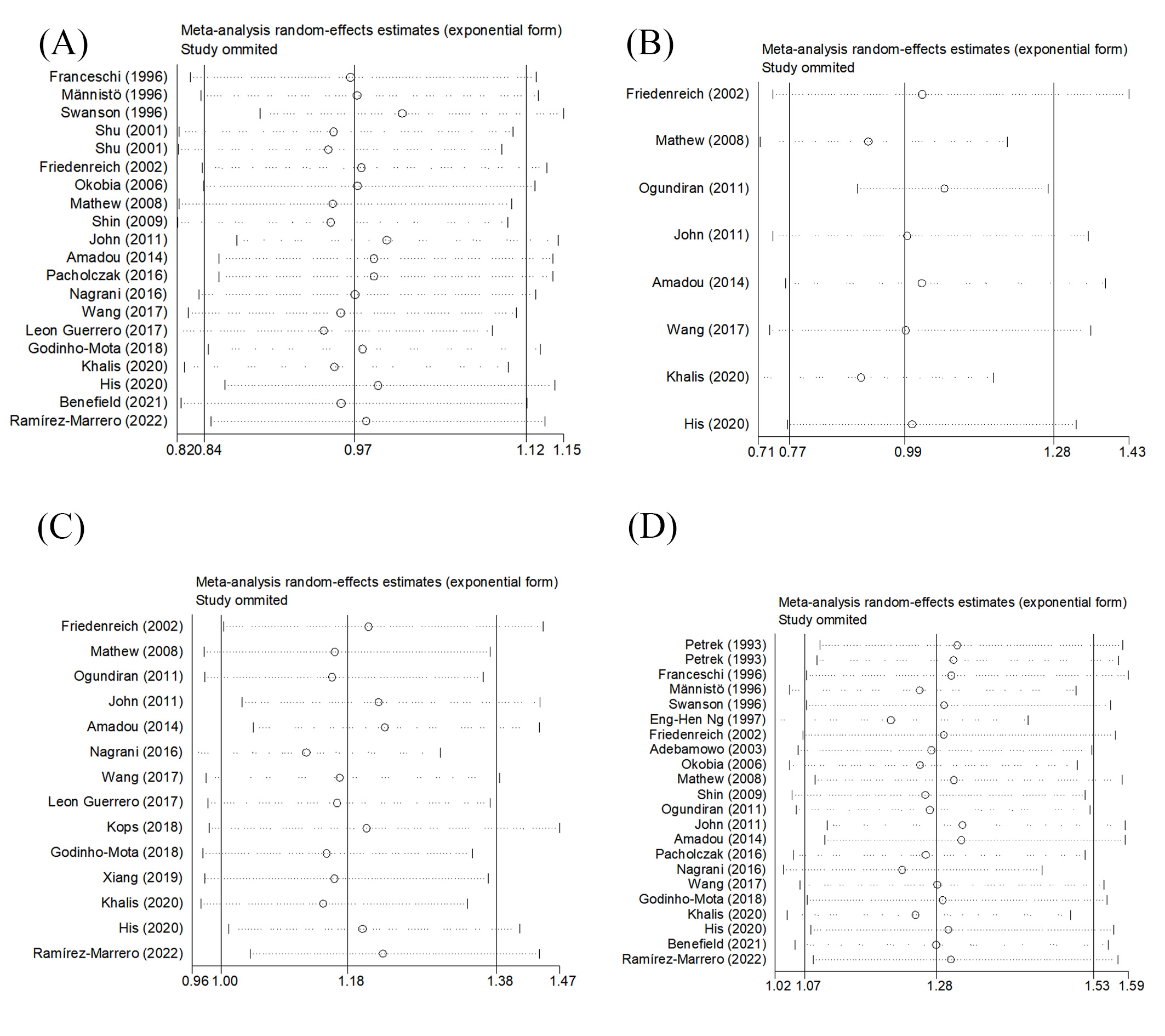

Supplement: Supplementary file 7 [file Image_1.jpg]

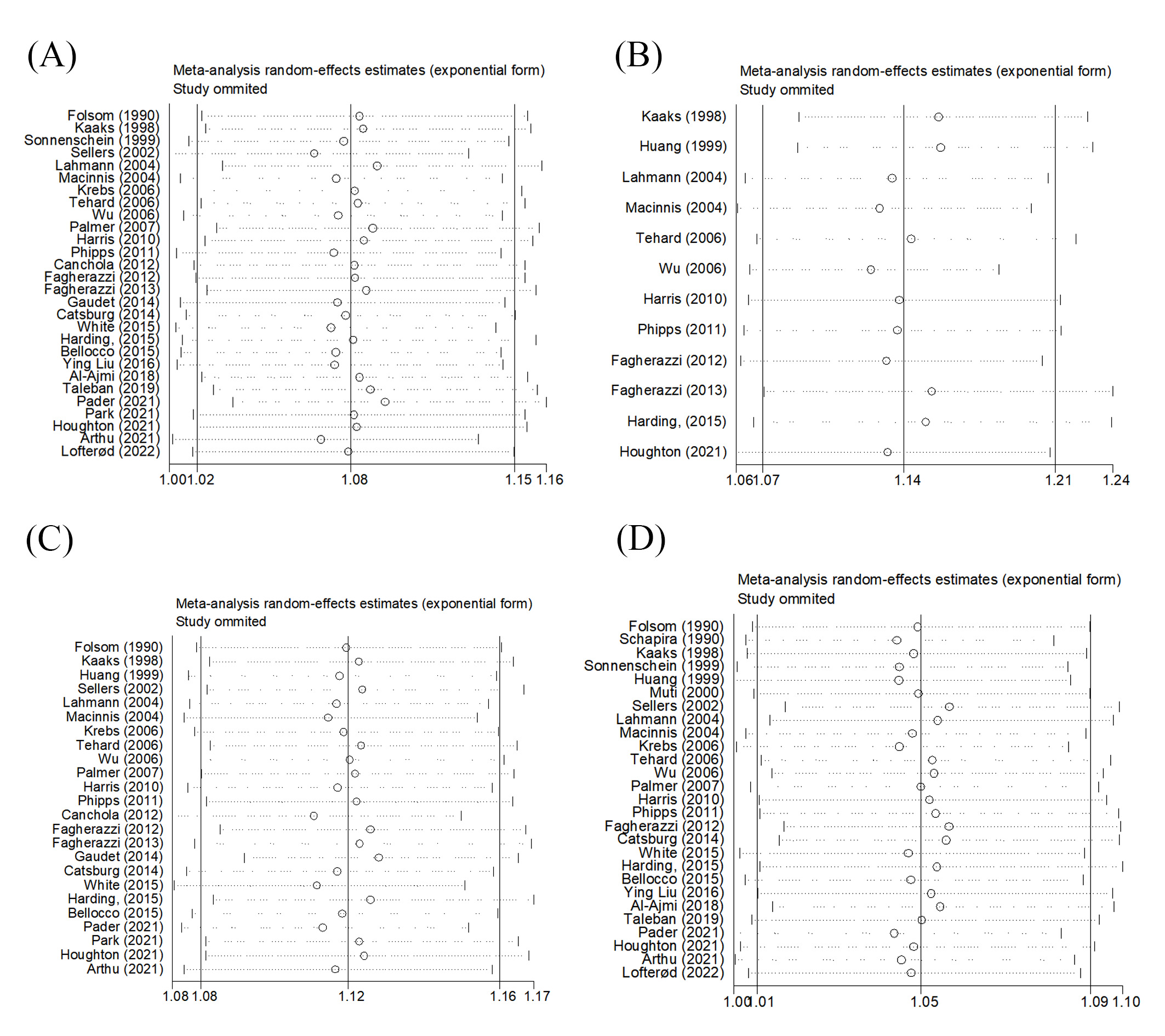

Supplement: Supplementary file 8 [file Image_2.jpg]
